# Proteomics in pancreatic cancer

**DOI:** 10.1186/s40364-025-00805-y

**Published:** 2025-07-06

**Authors:** Fei Cai, Yufan Gu, Yingying Ling, Guanhua Yi, Shengze Zang, Tao Su, Yueqiu Liu, Ang Li, Denian Wang, Wanjun Zhao, Xinfang Xie, Guisen Li, Lunzhi Dai, Meng Gong, Hao Yang, Yang Zhao, Yong Zhang

**Affiliations:** 1 Department of Pancreatic Surgery and Institutes for Systems Genetics, West China Hospital, Sichuan University, Chengdu, 610041 China; 2 Precision Medicine Center, Precision Medicine Key Laboratory of Sichuan Province, State Key Laboratory of Respiratory Health and Multimorbidity, West China Hospital, Sichuan University, Chengdu, 610041 China; 3 Division of Thyroid Surgery, Department of General Surgery, West China Hospital, Sichuan University, Chengdu, 610041 China; 4 Department of Nephrology, The First Affiliated Hospital of Xi’an Jiaotong University, Xi’an, 710061 China; 5 Renal Department, Institute of Nephrology, Sichuan Provincial People’s Hospital, Sichuan Clinical Research Center for Kidney Diseases, University of Electronic Science and Technology of China, Chengdu, 611731 China; 6 National Clinical Research Center for Geriatrics, State Key Laboratory of Biotherapy, West China Hospital, Sichuan University, Chengdu, 610041 China; 7 Transplant Center and NHC Key Lab of Transplant Engineering and Immunology, West China Hospital, Sichuan University, Chengdu, 610041 China; 8 Technology Innovation Center of Mass Spectrometry for State Market Regulation, Center for Advanced Measurement Science, National Institute of Metrology, Beijing, 100029 China

**Keywords:** Pancreatic cancer, Pancreatic ductal adenocarcinoma, Proteomics, Mass spectrometry, Biomarkers

## Abstract

Pancreatic cancer (PC), one of the most aggressive malignancies, is characterized by a dismal prognosis owing to its low early detection rates, rapid progression, frequent postoperative complications, and limited efficacy of conventional oncological therapies. The fact that most patients are diagnosed at advanced stages underscores the critical importance of early detection for the formulation of effective treatment strategies. Despite substantial research efforts, the medical community still lacks consistent and dependable biomarkers for the diagnosis, classification, and prognosis of PC, highlighting the urgent need for innovative and more efficient approaches to identify pancreatic abnormalities at early stages. For decades, mass spectrometry (MS)-based proteomics has been extensively applied in disease diagnostics, mechanistic investigations, and screening of potential drug targets. This review systematically synthesizes recent advancements in clinical proteomic techniques and applications, highlighting significant biomarker discoveries and signal transduction networks associated with PC. By integrating these findings, we provide novel insights into the molecular mechanisms underlying PC development and progression, which may facilitate the identification of new diagnostic biomarkers and therapeutic targets for this disease.

**Supplementary Information:**

The online version contains supplementary material available at 10.1186/s40364-025-00805-y.

## Introduction

Pancreatic cancer (PC) is the deadliest type of cancer in humans. Pancreatic ductal adenocarcinoma (PDAC) is the predominant subtype accounting for approximately 85% of cases and has a median overall survival time of approximately 12 months [1]. Despite advances in diagnosis and treatment over the past few decades, the 5-year survival rate is about 13%, with an average survival duration of 12 months after diagnosis [1, 2]. This is attributed to its increasing incidence, the lack of accurate early diagnostic biomarkers, the absence of preventive screening, and the scarcity of effective treatments.

The challenges in combating PC are multifaceted. Early detection is notoriously difficult because individuals rarely exhibit specific symptoms until the cancer has advanced. Currently, diagnosing PC relies primarily on computed tomography (CT) and/or magnetic resonance imaging (MRI), often in conjunction with magnetic resonance cholangiopancreatography (MRCP), biopsy, or fine-needle aspiration via endoscopic ultrasound (EUS). Owing to the lack of distinct symptoms and the limited effectiveness of current biomarkers (e.g., CA19–9) for early identification, about 80% of PDAC patients are diagnosed at the metastatic stage, when surgical removal is no longer possible. Even for patients eligible for surgical resection and adjuvant therapy, the risk of disease recurrence remains high [3]. Current therapeutic options, such as cytotoxic chemotherapy, offer only marginal survival benefits. The development of targeted therapies is further hindered by the complex heterogeneity of PC tumors, which often exhibit diverse molecular profiles and resistance mechanisms [4, 5]. However, developing reliable biomarkers with high sensitivity and specificity could enable curative resection of PC and ultimately reduce its high mortality rate. The next crucial step is to connect clinical proteomic research with practical applications in healthcare, paving the way for innovations that improve patient care.

Proteomics is a crucial complement to genetic research. Mass spectrometry (MS)-based proteomic analysis of clinical samples is a powerful approach for identifying biomarkers, pinpointing viable therapeutic targets, and studying signal transduction networks and regulatory mechanisms [6]. It allows for the quantitative analysis of proteins and their posttranslational modifications (PTMs). By facilitating the discovery of novel biomarkers, these methodologies improve diagnostic accuracy, prognostic evaluation, and therapeutic monitoring in clinical practice.

This review provides an in-depth look at the significant role that proteomics plays in PC research, particularly its potential to transform early detection and treatment strategies and clarify related molecular mechanisms. We begin with a concise overview of contemporary diagnostic and therapeutic approaches for PDAC, followed by a discussion of the historical evolution and principal technological methodologies in proteomics. We emphasize the challenges in managing PDAC and the urgent need for innovative diagnostic and therapeutic strategies. We then delved into the role of proteomics in identifying novel biomarkers and therapeutic targets while dissecting the molecular processes underlying PDAC oncogenesis, progression, and chemoresistance. By integrating recent advances in proteomics, we highlight key breakthroughs that could enable more precise and quicker diagnoses of PDAC. Finally, we discuss the translational potential of these findings, emphasizing how proteomics can connect laboratory research with clinical practice, creating a transformative framework that bridges PDAC biology to precision therapeutic development, ultimately improving outcomes for PDAC patients.

## Overview of pancreatic cancer

### Risk factors for PC

**Fig. 1:**
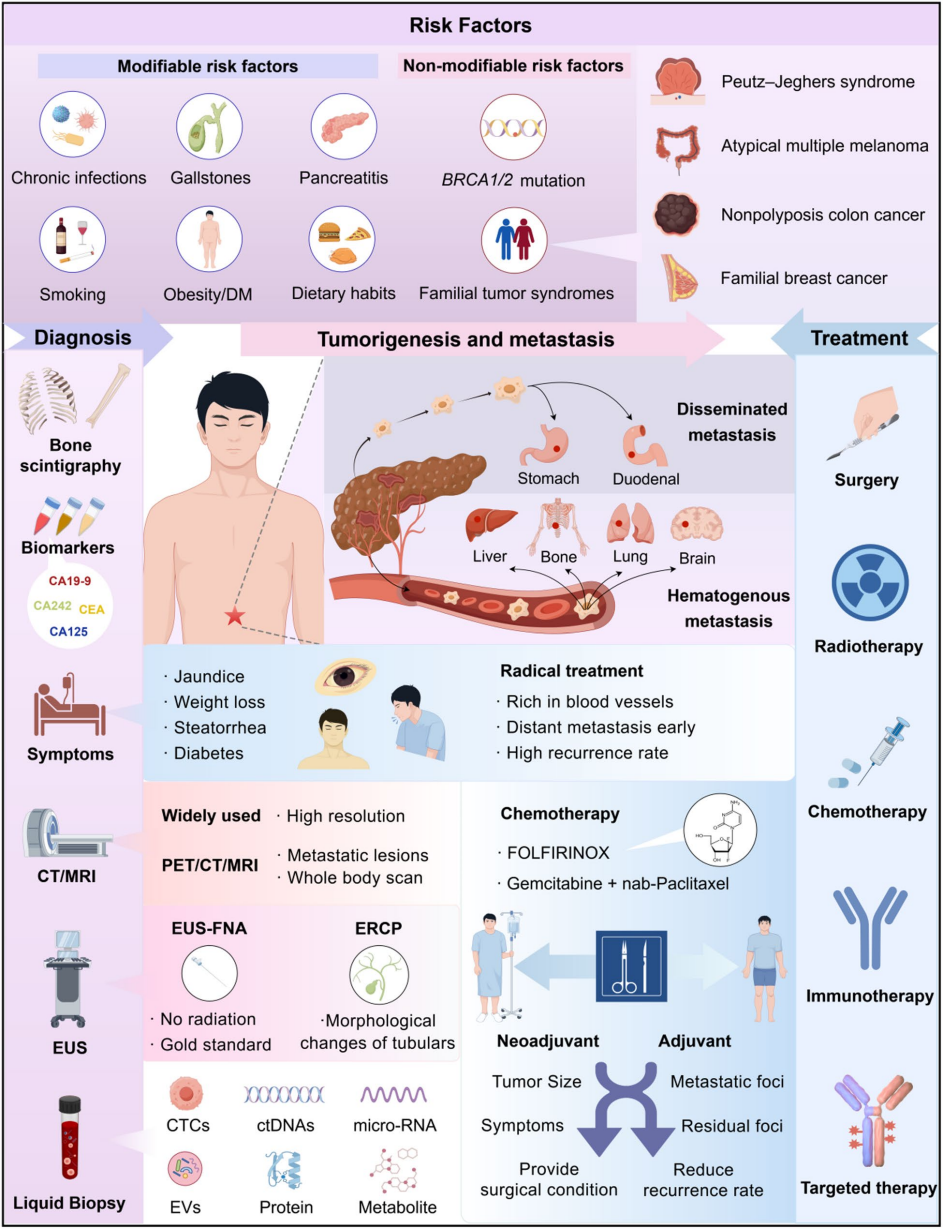
Comprehensive overview of PC diagnosis and treatment. In recent years, significant advancements have been made in diagnostic technologies for PC. In addition to traditional imaging techniques and pathological diagnosis methods, liquid biopsies and artificial intelligence methods are gradually being integrated into clinical practice. Treatment options for PC vary, with recent clinical studies highlighting the importance of individualized and precision treatments. Long-term follow-up management for PC patients is also essential, as it can influence therapeutic outcomes and enhance patients’ quality of life. Abbreviations: CT, computed tomography; MRI, magnetic resonance imaging; EUS, endoscopic ultrasonography; ERCP, endoscopic retrograde cholangiopancreatography; CTCs, circulating tumor cells; ctDNA, circulating tumor DNA; EVs, extracellular vesicles. The figure was created by Figdraw (www.figdraw.com)

The exact etiology of PC remains unclear. However, several risk factors have been associated with its development. Nonmodifiable risk factors include age, sex, ethnicity, ABO blood type, microbiota composition, diabetes mellitus (DM), family history and genetic predisposition. Modifiable risk factors include smoking, alcohol consumption, dietary patterns, pancreatitis, obesity, infections, and socioeconomic status or insurance coverage (Fig. 1) [7]. Genetic factors and environmental influences are key contributors to PC, with approximately 10% of cases being genetically linked. Certain familial tumor syndromes are associated with a greater risk of developing PC. Notably, loss-of-function mutations in the BRCA1 and/or BRCA2 genes are linked to an increased risk of ovarian, breast, and pancreatic cancers. Several nongenetic factors contribute to the risk of developing PC, including prolonged smoking, DM, pancreatitis, family history of certain cancers, adiposity, alcohol consumption, gallstones, dietary factors, and some chronic infections [8]. Among these factors, inflammation plays a crucial role in pancreatic carcinogenesis. Both acute pancreatitis (AP) and chronic pancreatitis (CP) significantly increase the risk of PC. In AP, particularly when caused by undiagnosed pancreatic tumors (e.g., tumor-induced obstructive pancreatitis), poses the highest PDAC risk within a year following the attack [9]. In CP, tissue damage can persist for decades, especially in cases of long-term, hereditary, and recurrent AP. This ongoing damage, along with inflammation, fibrosis, and cellular DNA damage, promotes tumor growth and progression [10]. Additionally, increasing evidence suggests that the pancreatic microbiome, which consists of bacteria, fungi, and other microorganisms, may contribute to the development and progression of PDAC [11]. These risk factors underscore the importance of follow-up imaging to identify early malignancies [12].

### Diagnosis of PC

PC is a highly malignant disease characterized by rapid progression and insidious onset, with early symptoms often being nonspecific. Consequently, most patients are diagnosed at advanced stages [13]. Common initial symptoms include jaundice, abdominal pain, weight loss, steatorrhea, and new-onset or worsening preexisting diabetes [14]. Imaging is an important tool for obtaining preliminary diagnoses and accurate staging of tumor diseases, and the scientific and rational use of various imaging methods plays an important role in standardizing diagnosis and treatment. According to the patient’s condition, choosing appropriate imaging technology is the premise of diagnosing pancreatic space-occupying lesions. Current diagnostic methods for this disease primarily involve imaging techniques, including CT scans and magnetic resonance imaging (MRI), which are frequently complemented by endoscopic ultrasonography (EUS), fine-needle aspiration (FNA), liquid biopsy, and artificial intelligence (Fig. 1) [15]. The strategic selection of imaging techniques tailored to the patient’s clinical presentation is essential for accurate diagnosis of pancreatic lesions and subsequent therapeutic planning. CT scans, known for their superior spatial and temporal resolution, are the best noninvasive imaging modality for evaluating the pancreas. However, their diagnostic accuracy is limited, highlighting the need for complementary methods [16]. MRI coupled with cholangiopancreatography (MRCP) offers a compelling alternative, with 84% sensitivity and a remarkable 97% specificity in detecting PC [17]. EUS can provide detailed views of the pancreas from a closer vantage point, significantly enhancing the likelihood of identifying small and localized lesions. EUS-FNA is a more precise technique for pinpointing and diagnosing PC. These imaging techniques are invaluable for visualizing pancreatic tumors, assessing vascular invasion, and determining precise clinical staging. While endoscopic retrograde cholangiopancreatography (ERCP) does not directly visualize tumors, it primarily involves evaluating morphological changes in the pancreatic duct and the common bile duct. Liquid biopsy, a noninvasive detection method, is more cost-effective and convenient than traditional diagnostic procedures are. By analyzing multiple components from a biofluid sample, such as circulating tumor cells (CTCs), circulating tumor DNA (ctDNA), cell-free DNA (cfDNA), microRNAs, long noncoding RNAs (lncRNAs), microRNAs (miRNAs), extracellular vesicles (EVs), tumor-educated platelets, tumor endothelial cells, proteins, and metabolites [18] liquid biopsy offers valuable insights into the molecular mechanisms of personalized tumorigenesis and progression. It also facilitates cancer diagnosis, real-time monitoring and prognosis [19]. Artificial intelligence (AI)-driven approaches offer promising new avenues for the early detection of PC. A groundbreaking multicenter study introduced an innovative AI method that integrates CT scans for large-scale early screening [20]. The model was trained via a single-center dataset involving 3,208 patients and validated across 10 centers with 6,239 patients, achieving exceptional diagnostic accuracy. This demonstrates its potential as a scalable screening tool for PC. AI algorithms are highly effective at detecting subtle pancreatic abnormalities by combining imaging data, biomarker profiles, and clinical information with remarkable accuracy [21]. However, several challenges persist, including limited access to comprehensive datasets, algorithmic bias during development, and concerns regarding data privacy and security, which hinder widespread implementation [22].

### Treatment of PC

Chemotherapy and surgery are the primary treatment options for PC (Fig. 1). However, only 15 to 20% of patients are eligible for surgical intervention [3]. Most PDAC patients are diagnosed with distant metastasis, making extensive surgery to remove the primary tumor unlikely to significantly improve the overall prognosis. In the clinical management of PDAC patients, systemic chemotherapy plays a crucial role irrespective of surgical eligibility. Over the past decade, two novel combination regimens have emerged as the preferred first-line therapies for advanced PDAC. The first regimen, known as FOLFIRINOX, combines 5-fluorouracil (5-FU), leucovorin, irinotecan, and oxaliplatin. The second regimen involves the combination of gemcitabine and an albumin-bound formulation of paclitaxel (nab-paclitaxel) [23]. For patients whose disease progresses on these regimens, alternative regimens or second-line options, such as liposomal irinotecan combined with 5-FU, may be considered if not previously administered [24]. However, the selection of second-line and subsequent regimens for treating PDAC lacks universal standards, with the treatment pathway often dictated by the patient’s performance status, the presence of actionable molecular targets, and the availability of suitable clinical trials. Supplementary Table 1 shows some recent clinical trials of palliative chemotherapy, immunotherapies and targeted therapy in patients with PC. Regrettably, even when surgical resection is feasible, nearly 75% of patients experience recurrence within 2 years, likely due to undetected micrometastases [25]. In support of this, preclinical studies have demonstrated that disseminated PDAC cells can be detected in the bloodstream before local invasion becomes evident in primary tumors. To increase the likelihood of eliminating PDAC cells that have disseminated beyond the primary tumor, patients who undergo surgery typically receive adjuvant chemotherapy after the operation [26]. Finally, one of the practices that has been increasingly adopted in the context of PDAC therapy over the past decade, albeit not uniformly around the globe, is systemic preoperative (neoadjuvant) therapy [27]. Neoadjuvant therapy has the potential to shrink “borderline” resectable PDAC and enable tumor resection. In fact, patients with PDAC who show a major or complete pathological response to neoadjuvant therapy show a significant improvement in overall survival (OS), although the biological underpinnings of this response remain to be discerned [28]. In recently completed randomized clinical trials, 18 neoadjuvant chemoradiations in conjunction with adjuvant chemotherapy resulted in significantly improved 5-year OS rates compared with patients who received upfront surgery with adjuvant therapy [29]. While surgery, radiotherapy, and postoperative adjuvant chemotherapy have improved the prognosis of patients with PC, the recurrence rate remains alarmingly high [30]. Therefore, it is extremely necessary to explore alternative treatment options. Immunotherapy, including vaccine therapy, is becoming increasingly popular in the management of recurrent, persistent, locally advanced, and metastatic diseases. However, at present, immunotherapy is not effective for most cases of PC. Various immunotherapy strategies have emerged, including specific and nonspecific immunotherapy, tumor vaccines, adoptive immunocyte therapy, and tumor factor therapy. Tumor vaccines, an important part of immunotherapy, prevent cancer or kill existing tumor cells by activating or restoring the body’s immune system. These vaccines include a range of types, including mRNA vaccines, cell-based vaccines, DNA vaccines, peptide vaccines, dendritic cell vaccines, nanovaccines, and viral vaccines [31]. Notably, tumor-specific neoantigens encoded by KRAS mutations have been identified as highly immunogenic targets for precision cancer vaccines to enhance antitumor immunity, with encouraging preclinical and clinical outcomes [32, 33]. Significant progress has been made in the development of PDAC vaccines over the past decade, demonstrating the safety and feasibility of personalized mRNA neoantigen vaccine production for treating resectable PDAC, as well as the combination of vaccines with other immunotherapies to induce more potent and durable T-cell responses [34]. By integrating various vaccine strategies with optimal sequencing of chemotherapy, surgery, radiotherapy, and other immunotherapies, we may further improve outcomes for patients with PC in the future.

Despite significant advancements in standard-of-care therapy for PDAC, achieving a cure remains challenging. Research indicates that early diagnosis and surgical resection of PDAC offer the best opportunity for long-term survival. Retrospective analyses of a national cancer surveillance database in the U.S. revealed that an impressive 80% of patients diagnosed with the earliest stages of PDAC survive for 5 years, whereas fewer than 10% of those with advanced disease servive [35]. Unfortunately, early-stage tumors are often detected incidentally, accounting for only 3–5% of cases. This has led to strong interest in the early diagnosis of PDAC [36]. In one large multicenter international trial involving high-risk groups, nearly three-fourths of patients diagnosed with PDAC during active surveillance presented with stage I disease. Among these patients, an equal proportion remain alive after 5 years, emphasizing that early detection can translate into better outcomes [37].

## Overview of proteomics

### History of proteomics

**Fig. 2:**
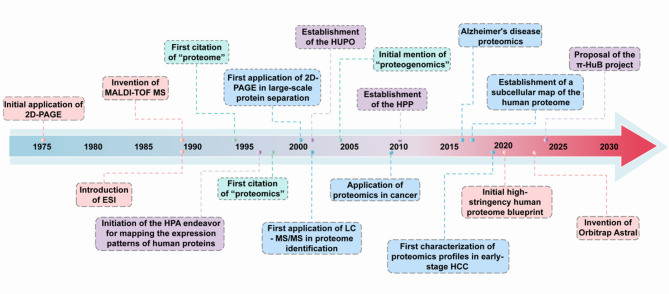
Significant milestones and breakthroughs in proteomics. Abbreviations: 2D-PAGE, two-dimensional polyacrylamide gel electrophoresis; ESI, electrospray ionization; MALDI-TOF-MS, matrix-assisted laser desorption/ionization time‒of‒flight mass spectrometry; HPA, human protein atlas; HUPO, human proteome organization; LC‒MS/MS, liquid chromatography‒tandem mass spectrometry; HPP, human proteome project; HCC, hepatocellular carcinoma

Proteomics is a vital discipline for investigating protein expression, structure, and function. Over the past few decades, it has become a cornerstone of biomedical research in the postgenomic era [38]. Key milestones and breakthroughs in the history of proteomics are illustrated in Fig. 2. Proteins, a class of biological molecules, were initially recognized in the 18th century. However, not until 1938 did Berzelius et al. formally proposed the concept of proteins, defining them as biological macromolecules composed of amino acids. A significant breakthrough occurred in 1949 when Sanger et al. successfully determined the amino acid sequence of bovine insulin, marking the first complete sequencing of a protein. This landmark achievement provided experimental confirmation that proteins are formed through the polymerization of amino acids, establishing a foundational paradigm in molecular biology. A major breakthrough in mass spectrometry occurred in 1989 with the introduction of matrix-assisted laser desorption/ionization time-of-flight mass spectrometry (MALDI-TOF-MS). This technology enables the ionization and analysis of proteins with relatively large molecular weights, fundamentally transforming the landscape of protein identification and research [39]. That same year, electrospray ionization (ESI) was also introduced, allowing for the generation of intact molecular ions from analytes in solution. These advancements facilitated the analysis of large biological molecules via MS [40]. By 1994, Wilkins et al. formally introduced the term ‘proteome’, defining it as “the entire set of proteins expressed by a genome, an organism, or a cell”, thereby establishing proteomics as a distinct scientific discipline [41]. Three years later, James et al. introduced the term “proteomics” for the first time, providing a systematic review of published research on the comprehensive analysis of protein species in living organisms and summarizing the advancements in this field up to that time. In 1996, Uhlén et al. launched the Human Protein Atlas (HPA), which leverages antibody technology and immunohistochemistry methods to visualize and assess protein expression across various human tissues and cells [42]. In 2001, the Human Proteome Organization (HUPO) was established, leading the charge of fostering global collaboration in the field of proteomics [43]. In 2010, the HUPO launched the international Human Proteome Project (HPP), which aims to integrate global resources for a comprehensive study of the human proteome. In 2017, Thul et al. established a comprehensive image-based subcellular protein distribution atlas known as the Cell Atlas [44]. In 2019, Chinese scientists performed the first comprehensive characterization of proteomic profiles in early-stage hepatocellular carcinoma (HCC) via quantitative proteomics. This study identified novel potential therapeutic targets for precision therapy in HCC, representing the beginning of the proteomics-driven precision medicine era [45]. Additionally, emerging technologies such as single-cell proteomics hold promise for addressing protein heterogeneity at the cellular level, thereby broadening the scope of the field [46]. Advancements in the speed, sensitivity, and coverage of MS are needed to significantly enhance proteomics research. For example, in 2023, Thermo Fisher Scientific’s Asymmetric Track Lossless Analyzer (Astral) achieved high resolution with MS/MS scanning speeds of up to 200 Hz [47]. Undoubtedly, these improvements in MS performance will allow researchers to directly access critical data from clinical samples [48]. In 2024, scientists led by He et al. proposed a “big science” initiative termed the human proteomic navigator (π-HuB) [49]. This project aims to establish a consortium of both Chinese and international researchers dedicated to generating extensive proteomic datasets from all major human tissues, organs, and cell types, followed by a comprehensive analysis of these data on an unprecedented scale. Its overarching goal is to develop an intelligent computational engine named the π-HuB navigator, which integrates multimodal proteomic datasets to transform our understanding of human biology, enhance disease risk assessment and diagnosis, identify new drug targets, optimize therapeutic strategies, and facilitate advanced healthcare solutions. This initiative marks the beginning of a new era in proteomics-driven “phronesis medicine”.

### Development of proteomic techniques

**Fig. 3:**
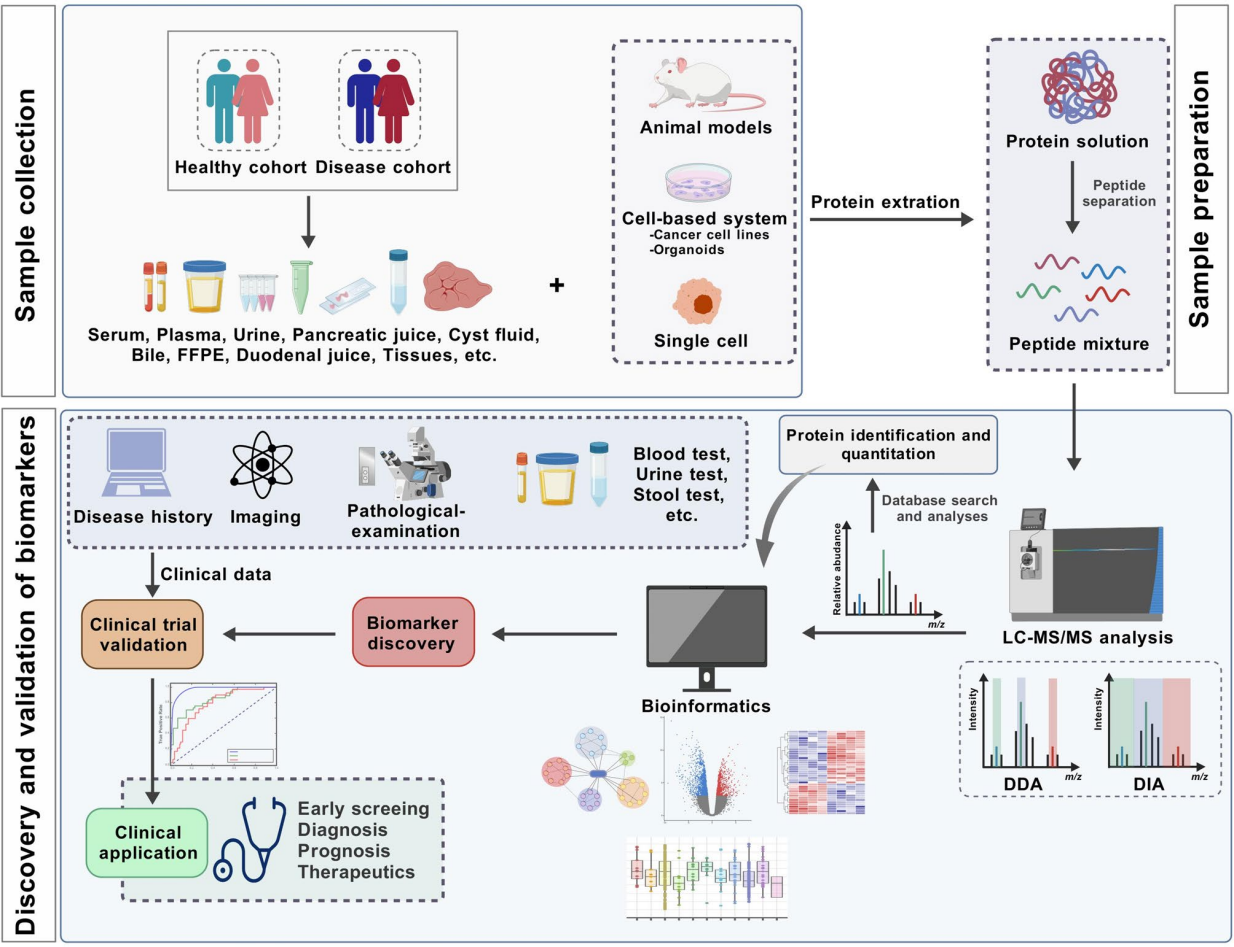
Schematic illustration of the PC proteomic workflow. Samples are first collected from both in vivo and in vitro sources. The samples subsequently undergo lysis to extract proteins, which are then enzymatically digested into peptide mixtures. These peptides are analyzed via LC‒MS/MS in either DDA or DIA acquisition mode. The acquired data are processed and visualized through database searching and bioinformatics analysis. Statistically significant proteins are then selected through rigorous statistical analysis. Finally, these candidate proteins are further validated in clinical populations via integration with clinical data for potential clinical translation. Abbreviations: FFPE, formalin-fixed paraffin-embedded; LC‒MS/MS, liquid chromatography‒tandem mass spectrometry; DDA, data-dependent acquisition; DIA, data-independent acquisition. The figure was created with BioGDP.com (https://BioGDP.com)

Conventional protein identification methods such as Western blotting, chemical sequencing, and gel electrophoresis are often time-consuming, low-throughput, and not well suited for large-scale proteomics studies [50]. However, MS has rapidly emerged as a lead technique in proteomics analysis [51]. It involves the ionization of analytes, followed by the separation of these ions according to their mass‒charge ratios (m/z) and subsequent detection of these ions to form a mass spectrum [52]. This powerful tool provides both qualitative and quantitative information, excelling in identifying unknown compounds, pinpointing the isotopic composition of molecules, and unraveling compound structures through fragment analysis [53]. In the field of proteomics, two main strategies stand out: the “bottom-up” approach and the “top-down” approach [54]. The “bottom-up” approach, which involves breaking proteins into peptides through enzymatic digestion before undergoing MS analysis, is predominantly utilized [55]. It is ideal for large-scale and high-throughput identification of proteins. Nevertheless, accurate determination of the full sequence, structure, PTMs, mutations, and isomers of the target protein is challenging [56]. In contrast, the “top-down” approach offers a comprehensive view of every protein variant and isoform generated by various genes. This method involves introducing intact proteins into high-resolution and high-sensitivity MS, fragmenting them, and then meticulously documenting the fragment masses [57]. However, this method has high requirements for sample preparation methods, purity requirements and data processing. Proteomic analysis workflows based on MS typically include many steps, such as sample preparation, chromatographic separation, mass spectrometry detection, and data analysis (Fig. 3) [58]. The specific methodology employed can vary depending on the biological question being addressed. Proteomic research has focused primarily on quantifying proteomes, studying PTMs, exploring protein‒protein interactions, and studying associations with disease [59]. Among these methods, quantitative proteomics stands at the heart of proteomics research, serving as a pivotal methodology for understanding protein complexities [60]. Quantitative proteomics methods primarily encompass chemical labeling and label-free analysis, offering a comprehensive toolkit for in-depth proteomic research [61]. Labeled quantification relies primarily on sophisticated techniques such as tandem mass tags (TMTs), isobaric tags for relative and absolute quantitation (iTRAQ), and stable isotope labeling with amino acids in cells (SILAC). Label-free quantification relies mainly on data-dependent acquisition (DDA) and data-independent acquisition (DIA) [62]. DIA is a comprehensive, high-throughput method performed by continuously scanning across a range of predefined m/z windows, ensuring coverage of all identified peptides. Its ability to gather extensive data, even for peptides of low abundance, has propelled DIA to the forefront of label-free proteomics [63]. In comparison, DDA focuses on peptides whose signal intensity surpasses a specific threshold for subsequent fragmentation analysis [64]. This approach proves invaluable for initial protein and PTM identification and quantification efforts [65].

Numerous proteomic strategies provide unique avenues for discovering and validating cancer-specific biomarkers. By analyzing various clinical samples simultaneously, researchers can gain a comprehensive and systematic understanding of patient variation, disease severity, and progression [66]. A broad range of clinical samples has been utilized in PC proteomics studies, including pancreatic tissues, blood (serum/plasma), pancreatic fluids, cystic fluid, urine, and bile, along with in vitro models such as PDAC cell lines and patient-derived xenograft (PDX) models (Fig. 3). Body fluid samples are commonly used to identify PC diagnostic biomarkers, whereas tissue samples, along with cell and animal models, are utilized to investigate its mechanisms. The specific findings can be found in the second half of this article. Recent advancements in MS techniques have expanded the methods available for PC proteomics [67]. These advancements allow for the quantification of disease-perturbing proteins. For example, Ansari et al. analyzed serum samples from patients with resectable PC, patients with benign pancreatic disease, and healthy controls, and identified specific serum protein alterations associated with early-stage PC [68]. Similarly, Samonig et al. emphasized the effectiveness of combining unbiased proteomics with targeted gene expression and functional studies to pinpoint new crucial regulators of tumor-initiating cells [69]. This strategy shows promise for future drug development efforts by identifying proteins and pathways of interest. By integrating large-scale protein profiling with functional validation, proteomics enables the systematic identification of dysregulated signaling pathways, tumor-specific biomarkers, and molecular subtypes, offering a transformative framework for deciphering PDAC biology and informing precision therapeutic development.

## Protein biomarkers for PC

### Clinical protein biomarkers

Identifying effective biomarkers for early-stage PDAC that can be detected via sensitive, specific, cost-effective and practical measurement methods remains a significant challenge. Several biomarkers are currently used in clinical settings for the diagnosis and monitoring of PC. Among these, CA19-9 is the most widely recognized and validated. CA19-9 is a modified Lewis (a) blood group antigen synthesized by exocrine epithelial cells that generally binds to the surface of erythrocytes and forms glycoproteins and mucins. It is the most commonly used serum tumor biomarker and is the only one approved by the U.S. Food and Drug Administration (FDA) for diagnosing PC in symptomatic patients, monitoring treatment efficacy, and surveilling for recurrence in patients with PDAC [70]. Additionally, American and European guidelines recommend checking CA19-9 levels in all patients prior to surgery or postresection treatment, as well as every 3 to 6 months for up to two years postsurgery [71]. However, CA19-9 has significant limitations: its sensitivity (50–75%) and specificity (about 83%) are suboptimal in asymptomatic populations, and elevated levels occur in benign conditions and other malignancies, such as gastrointestinal cancers, necessitating corroborative imaging (CT/MRI) for accurate diagnosis [72]. The utility of CA19-9 screening in asymptomatic individuals is therefore questionable [73]. Moreover, it is not informative for about 10% of the population who are Lewis antigen-negative, significantly limiting its use as a standalone screening tool [74]. Consequently, there is a need for biomarkers with increased sensitivity and specificity, and combining them with other markers may increase diagnostic accuracy and facilitate earlier detection of PDAC in the future [75]. To overcome these limitations, researchers have explored other biomarkers, such as carcinoembryonic antigen (CEA). Initially, recognized as a biomarker for colorectal cancer in the 1960s, CEA has since been found to be elevated in 30–60% of PDAC patients, particularly those with advanced disease, poor prognosis, and reduced overall survival [76]. While CEA alone is not sufficient for diagnosis, it improves prognostic stratification when used alongside CA19-9 and CA125, especially in predicting chemotherapy resistance and surgical outcomes [77, 78]. Importantly, CEA is a stronger independent predictor of advanced PDAC progression than CA19-9 is and provides a crucial alternative in cases where the Lewis antigen is negative, serving as a valuable diagnostic option when CA19-9 results are inconclusive [78]. The limitations of CA19-9 and CEA highlight the need for novel biomarkers with greater accuracy. Proteomic technologies provide a complementary approach to genomics by directly analyzing protein expression, offering insights into functional changes related to PDAC that may not be evident at the genomic level. While classical serum protein biomarkers (e.g., CA19-9 for diagnosis/monitoring, CEA for postoperative monitoring) are clinically established, non-protein biomarkers such as circulating tumor DNA (ctDNA) [79, 80] RNA signatures (e.g., miR-21) [81] metabolites [82, 83] and circulating tumor cells (CTCs) [84] show significant potential for early detection, prognostic evaluation, and treatment selection (Table 1). However, this review specifically focuses on proteomic biomarkers, and thus a detailed discussion of non-protein markers is beyond its scope. Advanced proteomic technologies can address the challenge of using nonspecific biomarkers for PDAC detection and enable the identification of PDAC-specific protein signatures by comparing proteomic profiles across clinical cohorts. These signatures, often linked to proliferation, invasion, and metastatic pathways, hold promise for early detection and personalized therapy. Unfortunately, the role of biomarkers in PDAC lags behind that in other cancers. For example, in breast cancer, hormone receptor status determines the need for endocrine therapy, and HER2 expression not only provides prognostic information but also influences treatment with anti-HER2 therapies [85]. In contrast, similar actionable biomarkers are lacking in PDAC. By combining proteomic insights with clinical validation, researchers aim to close this gap, developing biomarkers that can facilitate early diagnosis, predict treatment responses, and ultimately enhance patient outcomes.

**Table 1:** Clinically relevant biomarkers in pancreatic cancer

Types	Biomarkers	Samples	Key clinical utilities	References
Protein	CA19-9	Serum	Diagnosis, screening, monitoring, use as a prognostic marker post-resection, and application as a predictive marker for chemotherapy response	[70, 86]
	CEA	Serum	Monitoring recurrence, predict prognosis	[78, 87]
	CA125	Serum	Metastasis predictor	[88]
	CA242	Serum	Early diagnosis	[89]
DNA	KRAS^G12D/V/R^	ctDNA, tissue	Therapy selection	[79]
	SMAD4 mutation	Tissue	Predict prognosis, therapeutic target	[90, 91]
	CDKN2A/p16 loss	Tissue	Aggressive subtype	[92]
RNA	microRNA-21	Plasma exosomes	Early diagnosis	[93]
	microRNA-155	Serum exosomes	Gemcitabine response	[94]
	HOTTIP lncRNA	Tissue	OS prediction	[95]
Metabolite	Nine metabolites + CA19-9	Blood	Early diagnosis	[82]
	Creatine, inosine, beta-sitosterol, sphinganine and glycocholic acid	Plasma	Early diagnosis, progress monitoring	[96]
Cells	CTCs	Blood	Predict prognosis	[97]
	CSCs (CD44±/CD133±)	Tissue	Assess recurrence risk, predict tumor metastasis and prognosis	[98, 99]
Other	Exosomal GPC1	Serum	Early diagnosis	[100]
	ctDNA fraction	Plasma	Treatment response monitoring, predicts poor outcomes	[101, 102]

*ctDNA* circulating tumor DNA, *OS* overall survival, *CTCs* circulating tumor cells

### Candidate biomarkers for PC diagnosis

In this section, we summarize recent developments in identifying proteomic biomarkers that can differentiate PDAC patients from healthy individuals and those with other conditions. Supplementary Table 2 shows recently identified diagnostic biomarkers for PDAC discovered through proteomics techniques. For example, C4b-binding protein α-chain (C4BPA), a regulatory component of the classical pathway produced by liver cells and activated monocytes, has been recognized as a novel serum biomarker for early PDAC detection and for distinguishing PDAC from other gastroenterological cancers [103, 104]. In another study, neutrophil gelatinase-associated lipocalin (NGAL) in urine was shown to be a potential diagnostic biomarker for early PDAC detection [105]. In addition, Song et al. identified a group of pancreatic-specific proteins, revealing that the expression of carboxypeptidase was significantly downregulated in PDAC tumor tissues, suggesting that it may serve as a novel biomarker for PDAC [106]. Below, we further elaborate on several candidate biomarkers for PDAC diagnosis.

#### Annexins

Annexins are a multigene family of calcium- and phospholipid-binding proteins that play important roles in calcium signaling, cell motility, differentiation, and proliferation. A proteomic study utilized an immunostaining assay to assess annexin A10 (ANXA10) expression in 155 primary human tissue samples, including normal pancreas, CP, PDAC, pancreatic intraepithelial neoplasia (PanIN), and intraductal papillary mucinous neoplasm (IPMN). These findings indicate a strong correlation between ANXA10 expression and the progression of pancreatic precursor lesions toward PDAC [107]. Similarly, ANXA1 and ANXA10 have strong diagnostic potential for distinguishing between intrahepatic cholangiocellular carcinoma and metastatic liver tumors derived from PDAC [108].

#### GPC1

Glypican-1 (GPC1) is a membrane-anchored protein that has been reported to be abnormally expressed in various cancers, suggesting its potential role in tumorigenesis. As a result, it is considered a potential clinical biomarker in blood tests. Studies have shown that GPC1 is overexpressed in both PC cell lines and tissues. Its expression levels are closely related to perineural invasion, pathological grade, and the clinical stage of the cancer, with higher levels correlating with poorer patient prognosis. This highlights its significant diagnostic and prognostic implications [109]. Furthermore, Melo et al. reported that GPC1 is a specific marker of cancer exosomes [110]. Researchers have employed flow cytometry to identify GPC1^+^ exosomes in the serum of patients with PDAC or CP and healthy individuals. The results revealed that the levels of GPC1^+^ exosomes in the serum of all 190 patients were significantly greater than those in healthy controls, demonstrating nearly perfect diagnostic accuracy with approximately 100% sensitivity and specificity. Additionally, GPC1^+^ exosomes in the serum were identified as independent disease-specific prognostic markers, surpassing the prognostic value of CA19-9. Similarly, Qian et al. reported that patients with advanced-stage PC had significantly higher levels of GPC1^+^ EVs than healthy individuals did [111]. Despite these findings, the use of GPC1 as a biomarker remains contentious. First, it is suitable as a prognostic marker of PDAC rather than a diagnostic marker [112, 113]. Second, it is not a tissue-specific protein. Finally, it remains uncertain whether any of the various active clotting factors can cleave GPC1, potentially leading to inaccurate results. Therefore, additional validation is necessary to ascertain the reliability of serum GPC1 in diagnosing PDAC.

#### Tu M2-PK

Tumor M2 pyruvate kinase (Tu M2-PK) is a pyruvate kinase isoenzyme that plays a crucial role as a rate-limiting enzyme in glycolysis. Research by Ventrucci et al. has demonstrated that Tu M2-PK levels in serum can serve as a valuable diagnostic indicator, particularly in advanced PDAC and CP, where levels are significantly elevated compared with those in healthy individuals [114]. However, its utility for the early detection of PDAC is limited.

#### CEACAMs

Carcinoembryonic antigen-related cell adhesion molecules (CEACAMs) belong to the immunoglobulin superfamily and are involved in cell‒cell communication. They can influence various signaling events related to mitogenesis, survival, apoptosis, differentiation, migration, invasion, three-dimensional tissue structure arrangement, angiogenesis, and immune response modulation [115]. Among the CEACAM family members, CEACAM1 is the most extensively studied and is overexpressed in PDAC. Research has demonstrated that CEACAM1 offers superior diagnostic sensitivity and specificity compared with CA19-9 for differentiating PDAC patients from healthy controls. Additionally, the combination of CEACAM1 and CA19-9 significantly improves diagnostic accuracy [116]. Furthermore, an analysis of CEACAM1 expression in patients with PanIN revealed a marked difference in expression levels between normal pancreatic ducts and those from patients with various grades of PanIN [117]. These findings underscore the potential of CEACAM1 as a promising indicator for PC and precancerous lesions.

#### RTKs

Receptor tyrosine kinases (RTKs) are a class of membrane proteins that are frequently targeted in precision medicine, offering significant potential as therapeutic targets and circulating biomarkers for early disease detection. In PC, the overexpression of classical RTKs underscores their promise as diagnostic biomarkers. For example, insulin-like growth factor binding proteins (IGFBPs), which are RTKs, have shown diagnostic ability in differentiating early-stage PDAC patients from healthy individuals [118]. Similarly, vascular endothelial growth factor-A (VEGF-A) is overexpressed in both PDAC and serous cystic neoplasms (SCNs) [119]. Another RTK, epidermal growth factor receptor (EGFR), is frequently overexpressed in PDAC and is associated with tumor aggressiveness and postoperative recurrence. Therapeutic strategies targeting EGFR, such as the use of erlotinib, have been approved for the treatment of PC. Additionally, predictive biomarkers such as the EGFR ligand angiogenin (ANG) may enhance patient selection for therapies based on erlotinib [120].

#### OPN

Osteopontin (OPN) is a phosphorylated glycoprotein synthesized by osteoblasts, arterial smooth muscle cells, various epithelial cells, activated T cells, and macrophages. Once produced, it is released into most bodily fluids. Its presence has been shown to influence the invasiveness of PC cells, with increased expression correlating with lower survival rates in PC patients [121]. In a previous study, OPN demonstrated superior effectiveness compared with CA19-9 in differentiating patients with IPMN from those with CP [122, 123]. Elevated OPN levels (> 102 ng/ml) may serve as a potential diagnostic biomarker to distinguish PC from chronic pancreatitis [124]. Furthermore, a diagnostic panel that includes OPN, tissue inhibitor of metalloproteinases-1 (TIMP-1), and CA19-9 demonstrates improved sensitivity and specificity [125].

#### MIC-1

Macrophage inhibitory cytokine-1 (MIC-1) is a unique member of the transforming growth factor-β (TGF-β) superfamily of cytokines, and was initially identified in activated macrophages [126]. It is highly expressed across multiple malignancies, including PDAC. MIC-1 levels are significantly elevated in both tumor tissues and the serum of PDAC patients compared with healthy individuals and those with benign pancreatic conditions [127]. It exhibits superior sensitivity and lower specificity than CA19-9. Importantly, MIC-1 levels are significantly increased in most PDAC cases, including those who are CA19-9-negative and in early-stage tumors. This highlights its potential as a complementary diagnostic biomarker for the early detection and postoperative monitoring of PDAC [128]. A larger prospective study involving 446 PDAC patients demonstrated that elevated serum MIC-1 levels are correlated with increased overall mortality, underscoring its prognostic value [129]. Furthermore, serum MIC-1 levels are significantly decreased in PDAC patients following curative resection but increase again upon malignant relapse, suggesting that MIC-1 may be instrumental in assessing prognosis and monitoring treatment responses in patients with PDAC [130].

#### SPARC

Secreted protein acidic and rich in cysteine (SPARC), also known as osteonectin or basement membrane protein 40 (BM40), is an extracellular matrix glycoprotein that plays a crucial role in various biological processes, such as tissue remodeling, wound healing, morphogenesis, and cell differentiation. In PC, SPARC is often subject to abnormal methylation and is involved in tumor-stromal interactions. Importantly, the expression of SPARC in peritumoral fibroblasts within the stroma is a predictor of poor prognosis for PC patients, whereas SPARC derived from tumor cells does not have prognostic significance [131]. Furthermore, Han et al. reported that the overexpression of SPARC in peritumoral fibroblasts correlated with reduced long-term survival, further emphasizing its stromal-specific prognostic utility [132]. Endogenous SPARC performs tumor-suppressive functions in PDAC cells by inhibiting malignant phenotypes. Its expression appears to be regulated by FGFR1 [133]. Clinically, SPARC could serve as a predictive biomarker for Nab-paclitaxel therapy, as the high SPARC group demonstrated significantly longer median overall survival after albumin paclitaxel treatment than the low SPARC group did (17.8 months vs. 8.1 months), highlighting its potential to guide personalized chemotherapy strategies [134].

#### MYB

The ranscriptional activator Myb (MYB) is a cellular progenitor of the v-Myb oncogene and encode an oncogenic transcription factor that regulates various cellular functions by controlling the expression of a wide array of genes [135]. In PC, MYB is frequently overexpressed and plays crucial roles in tumor growth, metastasis, and desmoplasia as an oncogenic transcription factor [136]. A study on the secretome of two paired PC cell lines revealed that MYB was either exogenously overexpressed (in BxPC3) or silenced (in MiaPaCa) via RNA interference. These observations open novel avenues for further research to understand the broader pathobiological implications of MYB in PC and improve clinical management through biomarker development [137]. In a case-subcohort study involving over 600 PC patients, Kartsonaki et al. identified several circulating proteins associated with both short- and long-term risks of PC. They reported time-varying associations between specific proteins (e.g., matrix metalloproteinase-7 (MMP7), hepatocyte growth factor (HGF), and tumor necrosis factor receptor superfamily member 9 (TNFRSF9)) and the risk of PC, noting that elevated levels are linked to increased short-term risk [138].

#### KRAS mutation-associated proteins

GTPase KRas (*KRAS*) mutations represent the earliest genetic alterations in pancreatic carcinogenesis, are detectable in more than 90% of PDAC cases and are frequently observed in precursor lesions such as low-grade PanINs and IPMNs [139]. Low-grade PanINs are associated with mutations that lead to the constitutive activation of KRAS, which is the major oncogenic driver of PDAC. This activation is regarded as the primary genetic event that initiates the disease. The constitutive activation of *KRAS* and persistent stimulation of downstream signaling pathways drive tumor cell proliferation, migration, metastasis, and metabolic reprogramming while also enabling evasion of the antitumor immune response [140]. Genomic analyses have further revealed that the progression of PDAC involves sequential accumulation of mutations: oncogenic *KRAS* activation occurs early in the disease, followed by the later inactivation of tumor suppressor genes (e.g., *TP53*, *SMAD4*, and *CDKN2A*), particularly in advanced or invasive stages [141]. These genomic mutations can profoundly alter protein characteristics, functions, and interactions across various biological levels. However, changes in the genome and transcriptome do not always align with proteome alterations at the functional level [142]. Proteomic studies have sought to bridge this gap by exploring KRAS-driven biomarkers. For example, single extracellular vesicle (sEV) analysis of plasma from early-stage PDAC patients revealed that *KRAS*^mut^ and/or *P53*^mut^ were positive proteins in most stage I cases, highlighting their diagnostic potential [143]. Additional investigations have identified KRAS^G12V^-associated proteins in PDAC patients, revealing that the levels of laminin-C2 (LAMC2) and pentraxin-3 (PTX3) in systemic circulation are significantly elevated compared with those in healthy individuals. These proteins may serve as candidate biomarkers for PDAC and increase the diagnostic sensitivity of CA19-9 [144].

**Fig. 4:**
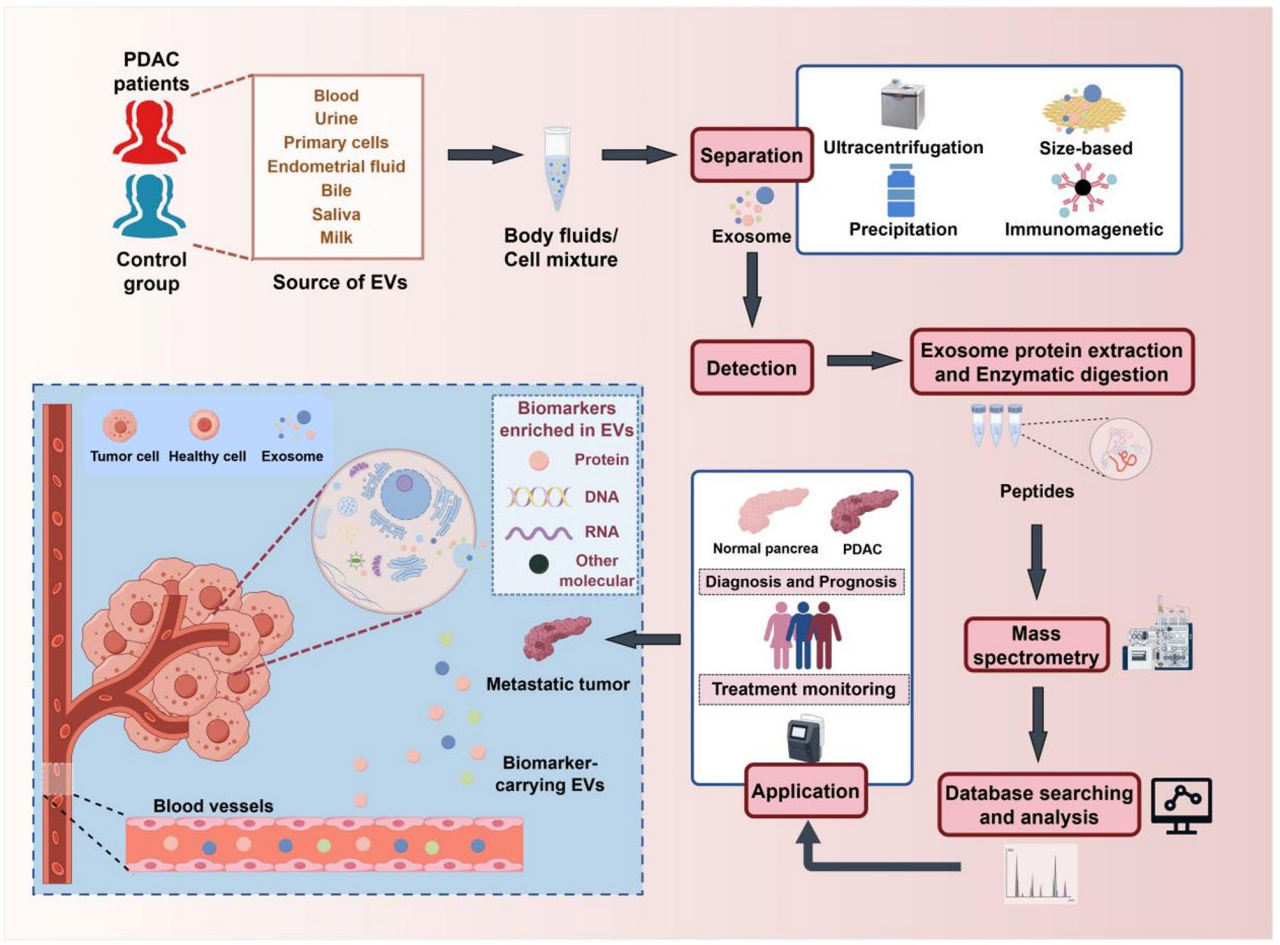
Mass spectrometry-based workflow for extracellular vesicle proteomics. PDAC-derived sEVs that circulate in the blood can be enriched by techniques such as ultracentrifugation. Molecular components, including proteins, can be analyzed to generate unique biomarkers for PDAC diagnosis/prognosis. Abbreviations: PDAC, pancreatic ductal adenocarcinoma; EVs, extracellular vesicles. The figure was created by Figdraw (www.figdraw.com)

### Extracellular vesicles in PC

Extracellular vesicles (EVs) are small membrane vesicles with a phospholipid bilayer structure that are typically disc shaped with diameters ranging from 30 to 5000 nm [145]. These vesicles can be broadly categorized into exosomes, microvesicles, and apoptotic bodies, each of which differ in origin, physical characteristics, and functional roles [146]. Among these, small extracellular vesicles (sEVs), particularly exosomes (30–150 nm), have attracted significant scientific interest over the past decade because of their critical involvement in various physiological and pathological processes. These processes include angiogenesis, apoptosis, inflammation, and immune regulation, which are influenced by the originating cells and their state at the time of sEVs production [147]. Exosomes, which are secreted by diverse cell types, serve as essential messengers in intercellular communication [148]. They play crucial roles in the formation of premetastatic niches within tumor cells, enhancing tumor aggressiveness and supporting the survival of metastatic cells. These findings position them as valuable resources for identifying biomarkers. They present significant advantages as diagnostic tools. They are widely found in biofluids, such as plasma and urine, allowing for noninvasive sampling that serves as an alternative to tissue biopsies. Notably, PDAC-derived sEVs contain many cancer-associated proteins linked to tumor progression, including those involved in mechanisms such as stromal infiltration, angiogenesis, and tumor microenvironment modulation. These features underscore their dual potential as both diagnostic biomarkers and therapeutic targets [149]. The sEVs secreted by PDAC cells can be easily collected from body fluids. Various isolation methods (e.g., ultracentrifugation, immunoaffinity separation, polymer precipitation separation, and size exclusion chromatography) can be used to enrich exosomes derived from PDAC samples. The molecular components (e.g., proteins, RNA, DNA, lipids, and glycans) can be analyzed via appropriate techniques (e.g., mass spectrometry, polymerase chain reaction, gel electrophoresis, and flow cytometry). These analyses are crucial for the diagnosis and prognosis of PDAC (Fig. 4). This review focuses on the proteins identified in sEVs associated with PDAC. sEVs secreted by PC cells have been implicated in initiating the malignant transformation of healthy cells. In contrast, sEVs derived from normal pancreatic cells are enriched with immune response proteins, which are significantly diminished in cancer-derived vesicles. These proteomic disparities between sEVs of malignant and nonmalignant origins may serve as valuable biomarkers for cancer detection [150]. Supplementary Table 3 provides a summary of EV-associated protein biomarkers that have diagnostic and prognostic relevance in PDAC. For example, proteomic profiling of EV-associated proteins combined with advanced purification methodologies has led to the discovery of novel biomarkers, such as G protein-coupled receptor class C group 5 member C (GPRC5C) and epidermal growth factor receptor pathway substrate 8 (EPS8), both of which show diagnostic potential for early-stage PC [151]. Additionally, exosomal ephrin type-A receptor 2 (EphA2) has been linked to chemoresistance and may serve as a predictive biomarker for the therapeutic response in patients with PC [152]. The pancreatic duct fluid (PDF) is in direct contact with both the tumor epithelium and stroma, making it an optimal medium for investigating PDAC. The protein composition of the exosomes in the PDFs was quantified via LC‒MS/MS to determine the feasibility of PDF exosome isolation and the clinical significance of measuring exosomal proteins. Notably, cell adhesion molecule 1/5 (CEACAM1/5) and tenascin (TNC) have emerged as potential biomarkers capable of distinguishing benign conditions from precancerous lesions [153]. Additionally, proteomic analysis of the exosomes revealed several PDAC-specific biomarker candidates. The surface proteins of sEVs are particularly intriguing, as specific surface markers unique to sEVs from particular cell types will facilitate their isolation. Therefore, the identification of surface markers is highly important for the development of diagnostic assays. A panel of PDAC-specific exosome surface proteins, referred to as the “surfaceome”, was identified through systematic screening. This panel includes LDN4, EPCAM, CD151, LGALS3BP, HIST2H2BE, and HIST2H2BF, which were subsequently utilized as immunocapture targets to enrich cancer-derived exosomes from liquid biopsies [154]. Capello et al. conducted comprehensive proteomic profiling of tumor-derived exosomes from PDAC cell lines and patient plasma, revealing diverse tumor-associated antigens on exosomal surfaces [155]. These antigens were found to inhibit complement-mediated cytotoxicity, facilitating tumor immune evasion. Notably, two exosomal membrane proteins (PKM2 and LGALSBP3) were significantly enriched in plasma-derived exosomes from early-stage PDAC patients compared with those from healthy controls, underscoring their diagnostic potential [155].

Some studies have compared the protein profiles of EVs derived from different tumors. One particular study compared the protein contents of EVs from three different malignancies: oral squamous cell carcinoma (OSCC), PDAC and melanoma brain metastasis cell lines. EVs isolated from each cancer cell line presented distinct protein profiles. For example, mucin 5AC (MUC5AC) was identified in PDAC, indicating its role in promoting tumor progression [156]. Furthermore, malignancy-specific biomolecular signatures found in EVs hold promise as potential diagnostic biomarkers and therapeutic targets. Galectin-3-binding protein (LGALS3BP), pyruvate kinase PKM (PKM2), heat shock cognate 71 kDa protein (HSPA8), cytokeratin-17 (KRT17), cytokeratin-16 (KRT16), cytokeratin-5 (KRT5), beta-actin (ACTB), and desmoplakin-3 (JUP) were found to be significantly enriched in the plasma EVs of PDAC patients compared with those in healthy individuals [155]. Furthermore, another study evaluated the prognostic significance of macrophage migration inhibitory factor (MIF) in exosomes derived from PC patients and revealed a notable increase in MIF levels among stage I PC patients who subsequently developed liver metastasis [157]. These investigations underscore the role of exosomes as prognostic biomarkers for predicting distant metastasis.

In recent years, EVs derived from dietary sources, particularly milk, have attracted considerable attention in oncology research. Milk exosomes, especially those originating from mammary glands, are being explored for their potential dual roles in cancer modulation due to their bioactive cargo [158]. Samuel et al. orally administered bovine milk-derived EVs to various mouse models and characterized them via electron microscopy, nanoparticle tracking analysis, western blotting, and quantitative proteomics [159]. These findings demonstrated that these bovine milk-derived EVs could be absorbed and were bioavailable in various organs, demonstrating their ability to modulate the proteomic profile of liver tissue. This finding supports the concept of cross-species communication [159]. Intriguingly, while bovine milk EVs reduce the primary tumor burden in colorectal and breast cancer models, they paradoxically accelerate metastatic progression in both breast cancer and PC. Specifically, oral administration of milk-derived EVs promoted the metastatic colonization of PC cells to the liver in PC mice [159]. Therefore, further research and clinical trials are essential before exosomes can be used as routine treatments and diagnostic tools for PC.

Overall, EVs hold significant promise as novel liquid biopsy biomarkers for the early diagnosis of PC. To date, research has focused on evaluating their diagnostic and prognostic utility in distinguishing PC patients from healthy individuals. However, their predictive role in therapeutic response and early diagnostic value across precancerous lesion stages (e.g., PanINs or IPMNs) remain underexplored, despite their critical importance for improving clinical outcomes. In terms of exosome utilization, further research should address whether other exosomal factors are resistant to PC or other cancer therapeutic agents and clarify the underlying mechanisms involved [154]. Importantly, while EVs offer unique advantages, such as their ubiquitous presence in biofluids and stability under long-term storage conditions, technical challenges in their isolation, purification, and standardization hinder their translation into clinical practice. Future research advancements are essential to overcome these obstacles and fully realize the potential of EVs in clinical settings.

### Multiplex biomarker panels in PC

Research has shown that relying on a single biomarker, such as CA19-9, is insufficient for providing a reliable diagnosis that is both sensitive and specific for PDAC. To overcome this limitation, the development of proteomics-driven multiplex biomarker panels has emerged as a promising approach to increase diagnostic accuracy, especially for early-stage disease [160, 161]. As highlighted in Supplementary Table 4, the combination of novel biomarkers with CA19-9 significantly improved the discriminatory power of PDAC detection. For example, a tripartite panel combining CA19-9, leucine-rich α-2 glycoprotein (LRG1) and metalloproteinase 1 (TIMP1) outperformed CA19-9 alone in distinguishing early-stage PDAC patients from healthy controls [162]. Further studies have identified additional biomarker panels that enhance diagnostic capabilities. Serum alpha-1-acid glycoprotein 1 (AGP1), validated through LC‒MS/MS and antibody-based assays, demonstrated improved diagnostic performance when combined with CA19-9 [163]. Similarly, thrombospondin 1 (THBS1), measured by MRM-MS in prediagnostic PDAC serum samples, decreased significantly 24 months before clinical diagnosis. The combination of THBS1 and CA19-9 achieved an AUC of 0.86, outperforming individual markers (AUC = 0.69 for THBS1; AUC = 0.77 for CA19-9; *p* < 0.01) [164] The integration of LRG1, transthyretin (TTR), and CA19-9 into a multimarker panel has demonstrated significant promise for early PDAC detection [165]. Combining CA19-9 with MUC5AC resulted in sensitivity and specificity values of 75% and 83%, respectively [75]. Another panel that includes CA19-9, apolipoprotein A-IV (APOA4), and TIMP1 demonstrated even greater accuracy in distinguishing early-stage PC from pancreatitis (AUC = 0.934; 86% sensitivity, 90% specificity) [166] Furthermore, a panel consisting of apolipoprotein E (APOE), interalpha-trypsin inhibitor heavy chain H3 (ITIH3), apolipoprotein A-I (APOA1), apolipoprotein L1 (APOL1), and CA19-9 significantly improved the diagnostic sensitivity (95%) and specificity (94.1%) of PC [167]. In addition to being used in combination with the traditional biomarker CA19-9 to form a biomarker panel, some panels that do not include CA19-9 demonstrate considerable diagnostic efficacy. A pioneering proteomic study identified a urinary biomarker panel comprising LYVE1, REG1A, and TFF1. It has potential for the early detection of PDAC [168, 169]. Further advancements in plasma-based biomarker discovery have led to the development of an eight-protein panel that includes S100 calcium-binding protein A11 (S100A11), PPY, proto-oncogene tyrosine-protein kinase receptor Ret (RET), 50-NT, integrin subunit beta 5 (ITGB5), receptor tyrosine-protein kinase erbB-3 (ERBB3), secretory carrier-associated membrane protein 3 (SCAMP3), and CEACAM1. This panel distinguished early-stage PDAC patients from healthy individuals with cross-validated AUCs of 0.85 (95% CI: 0.78–0.91) and 0.81 (95% CI: 0.70–0.92) in Swedish and Spanish cohorts, respectively [170]. Another 14-protein multimarker panel significantly outperformed CA19-9 alone across training and validation sets (AUC 0.977 vs. 0.872, *p* < 0.001), highlighting its clinical promise as a complementary tool [171].

Despite these promising results, several limitations persist. For example, multianalyte testing strategies inherently increase the risk of false-positive results, necessitating population-specific adjustments. Screening protocols for high-risk cohorts (e.g., individuals with hereditary PDAC predisposition, pancreatic cystic lesions, and new-onset diabetes) may require distinct positivity thresholds compared with those applied in the general population, balancing sensitivity and specificity to optimize clinical utility [172]. Furthermore, most multimarker panels are still confined primarily to exploratory studies and lack validation in large-scale, controlled clinical trials. Future research should prioritize rigorous validation of these combinations in diverse cohorts and standardized settings. Additionally, advancing methodologies to integrate diagnostic and prognostic biomarkers could further refine the sensitivity and specificity of PDAC detection strategies. Multivariate analyses have underscored the critical role of multimarker panels in advancing the early diagnosis of PDAC [173]. While these panels hold significant promise, the development of tissue biomarkers for MS-based proteomics, particularly those that can predict recurrence or chemotherapeutic sensitivity, remains in its nascent stages. To bridge this gap, PDAC proteomics must build upon the strengths and limitations of existing studies, employing sophisticated computational and experimental approaches to integrate diagnostic and prognostic signatures. Such integration is essential to increase the sensitivity and specificity of individual biomarkers while ensuring their clinical applicability. The ideal diagnostic biomarkers for PDAC should meet two criteria: (1) detectability via minimally invasive methods (e.g., blood or urine) and prioritize biomarkers detectable in blood or urine with high specificity to distinguish PDAC from benign conditions. (2) Robust discriminatory power to distinguish patients with PDAC from healthy individuals or those with benign pancreatic diseases. However, current multimarker panels face critical challenges. Most validation cohorts lack ethnic diversity, limiting generalizability. For clinical translation, panels must undergo rigorous external validation across multiethnic populations and genetic backgrounds and standardized laboratory protocols to ensure reproducibility [171].

### Biomarkers in CA19-9-negative PDAC

Approximately 5–10% of the general population carries the Lewis^a−b−^ phenotype, which prevents the synthesis of the CA19-9 antigen. This characteristic renders conventional CA19-9 testing ineffective for PC detection in these individuals [167]. To overcome this limitation, alternative protein biomarkers have been explored. Alongside previously mentioned markers such as CEA, MIC-1 and DTNBP1, emerging evidence highlights that apolipoprotein A-1 (APOA-1) and transferrin (TF) may serve as serum biomarkers for CA19-9-negative PDAC patients [174]. Another targeted proteomic study revealed that proline-hydroxylated α-fibrinogen may serve as a biomarker for detecting early-stage cancers among CA19-9-negative patients [175].

### Prognostic biomarkers

The prediction of PDAC prognosis is important for assessing the likely health outcomes of cancer patients (e.g., overall survival, disease recurrence). Identifying markers associated with PDAC tumor progression and aggressiveness can aid in selecting appropriate treatment strategies, enhancing our understanding of patient response to treatment, and in certain cases, managing the disease without treatment. A summary of recent proteomic studies focused on predicting PDAC prognosis is provided in Supplementary Table 5. Specifically, calreticulin (CRT) is an endoplasmic reticulum (ER)-resident protein involved in various cellular processes. It is highly expressed in PC stem-like cells and is associated with poorer survival rates in PC patients following radical resection [176, 177]. In another study, the protein tyrosine phosphatases PTPRM and PTPRB were found to be decreased in the plasma of PDAC patients with poor prognoses. Conversely, the expression of the proteasome subunit PSMD11 was increased in microparticle samples from these patients [178]. Additionally, cytokeratin-19 has emerged as a potential prognostic marker for advanced PC. The level of cytokeratin-19 prior to treatment was independently associated with performance status (*p* = 0.0399) and disease stage (*p* = 0.0001) [179] Other proteins, such as the SLC16A3, SLC16A13, and S100 proteins, have also been identified as potential biomarkers for the prognosis of PC [180, 181].

Circulating tumor cells (CTCs), characterized by their high metastatic potential and viability, are tumor cells shed into the peripheral blood from primary or metastatic lesions. In PDAC, CTCs demonstrate prognostic and therapeutic relevance. A study involving 55 PDAC patients who underwent longitudinal assessment of CTCs and RARRES1 protein expression both before treatment initiation and during follow-up revealed that RARRES1-positive patients presented elevated CTCs and poorer prognoses after curative surgery [182]. Molecular profiling of CTCs further underscores their clinical utility. Ankeny et al. separately analyzed KRAS mutations in CTCs and primary tumor tissues from five PDAC patients and reported complete consistency (100%) between these results [179]. However, the application of CTCs in PDAC presents several challenges. PDAC typically generates low numbers of CTCs, making the efficient isolation and enrichment of CTCs a challenging process. Additionally, variability in detection platforms limits clinical reproducibility.

### Biomarkers associated with treatment response monitoring

In recent years, proteomics technologies have revolutionized the systematic analysis of global protein expression dynamics in tumor cells. By identifying differentially expressed proteins between tumor and normal cells across different stages of PC (before treatment, during treatment, and after treatment), proteomic approaches can facilitate the discovery of novel therapeutic targets and enable the development of personalized cancer treatment strategies [183]. These methodologies also empower clinicians to refine prognostic predictions, optimize treatment selection, and mitigate adverse drug effects through biomarker-guided decision-making [184]. CA19-9 is widely used to monitor treatment response and predict the recurrence of PDAC. While CA19-9 remains the cornerstone biomarker for these purposes, several emerging protein biomarkers are enhancing clinical applications. For example, cytokeratin 19-fragments (CYFRA 21 − 1), a biomarker validated in several epithelial malignancies, has prognostic value in patient with PDAC. Elevated pretreatment CYFRA 21 − 1 levels are correlated with reduced OS and may predict chemotherapy resistance, making it a useful tool for assessing the prognosis of advanced PDAC patients [179]. Additionally, a recent cross-sectional study confirmed its relevance, showing that CYFRA 21 − 1 levels correlate strongly with PDAC tumor stage and remain unaffected by jaundice, unlike CA19-9 levels do [185]. A large-scale prospective observational study in China analyzed 191 treatment-naïve tumors alongside 90 paired tumor-adjacent tissues for comprehensive proteomic evaluation [186]. This study established a prognostic risk model for PDAC patients and identified two biomarkers (NDUFB8 and CEMIP2) that predict adjuvant chemotherapy sensitivity.

Neoadjuvant therapy is a standard treatment for rectal and gastroesophageal cancers. While early- or intermediate-stage rectal cancer patients typically proceed directly to surgery, those with locally advanced disease often receive neoadjuvant radiotherapy. This approach improves local control, although it does not increase the median OS [187]. In contrast, neoadjuvant chemotherapy (NAC) has demonstrated clear survival benefits for patients with locally advanced gastroesophageal cancers, with randomized trials reporting improved OS outcomes [188]. Emerging evidence highlights the systemic effects of NAC beyond tumor regression. Proteomic analyses of NAC-treated tumors revealed marked downregulation of proteins involved in metabolic pathways, suggesting that altered tumor energetics are a potential mechanism of the therapeutic response [189]. Additionally, patients who underwent NAC presented reduced serum lactate and high-density lipoprotein-cholesterol levels. In the context of PDAC, NAC is increasingly adopted for patients with borderline resectable and locally advanced disease. Notably, residual cancer cells surviving NAC presented elevated expression of cancer stem cell (CSC) markers both in vivo and in vitro. This finding underscores their significant role in therapeutic resistance and disease recurrence [190]. SWATH-MS analysis of NAC-treated PDAC tissues revealed 3,156 proteins, 19 of which were significantly differential expressed. Furthermore, GRP78, CADM1, PGES2, and RUXF demonstrated robust predictive potential for poor NAC response, with AUC values ≥ 0.92 [191] These findings support the development of proteome-guided prognostic frameworks and personalized treatment strategies.

Efforts to refine prognostic biomarkers now extend to immune profiling. Mandili et al. developed a proteomic method to isolate serum-derived IgG, IgM, and IgA circulating immune complexes, suggesting its utility in predicting the response of PDAC patients to chemotherapy [192]. Similarly, a comparative plasma proteomic analysis of PDAC patients stratified by therapeutic response revealed a biomarker panel comprising protein Z (PZ), sex hormone-binding globulin (SHBG), von Willebrand factor (VWF), and CA19-9. This panel effectively distinguishes between patients with a positive therapeutic response and those with longer OS (good responders) versus those who do not respond and who have shorter survival times (limited responders) [193]. Recent studies have provided mechanistic insights into chemoresistance. SILAC-based proteomics identified 107 differentially expressed proteins in oxaliplatin-resistant PDAC cells, pinpointing MARCKS and WLS as key regulators of Wnt/β-catenin signaling-driven resistance [194]. Furthermore, proteomic characterization of PSCs revealed distinct protein signatures associated with gemcitabine resistance, highlighting their role in shaping the chemoresistant tumor microenvironment [195].

In summary, these findings highlight the transformative role of proteomic signatures in predicting therapeutic responses and clinical outcomes in PC patients. Thesestudies have also revealed disease-specific molecular mechanisms related to tumorigenesis, progression, and therapeutic resistance. Through proteomic analyses, prognostic and predictive tools based on actionable protein biomarkers have been developed. These advancements not only enhance our understanding of the underlying mechanisms but also open new avenues for precision oncology strategies, ultimately improving patient stratification, therapeutic targeting, and overall clinical management of this challenging disease.

## Therapeutic targets

### Candidate therapeutic targets for PC

Gemcitabine is known to be the first-line chemotherapeutic agent for the treatment of PC. However, owing to the challenges of resistance and metastasis associated with PC, continuous exploration of new therapeutic targets and agents is essential. Additionally, enhancing the sensitivity of cancerous tissues to treatment pathways, including those involving gemcitabine, is vital for improving patient outcomes. Recent proteomic approaches have revealed promising therapeutic targets for treating PDAC. A study using PDAC cell lines revealed that the mRNA binding protein cold shock domain containing E1 (CSDE1) was overexpressed in PC cell lines but absent in normal pancreatic epithelial cells. Further functional verification experiments revealed that CSDE1 could be a promising therapeutic target to inhibit PC cell invasion [196]. Cai et al. reported that MICAL1 is overexpressed in PDAC tissues and cell lines. Knocking down MICAL1 inhibited proliferation, invasion, and metastasis both in vitro and in vivo. Additionally, RNA sequencing analysis suggested a strong correlation between MICAL1 and the WNT signaling pathway [197]. Bone morphogenetic protein type II receptor (BMPR2) is a cell surface receptor that plays a crucial role in the BMP signaling pathway. It is overexpressed in PDAC tissues, with higher levels correlating with poorer overall survival rates. It has been demonstrated to inhibit PDAC growth by regulating the GRB2/PI3K/AKT axis, resulting in reduced cell proliferation and G2/M cell cycle arrest [198]. Phosphoglycerate mutase 1 (PGAM1) is a key glycolytic enzyme that has been found to be overexpressed in PDAC tissues associated with clinical metastasis, as well as in highly metastatic PC cell lines [199]. Silencing PGAM1 significantly reduces PDAC cell migration and invasion, underscoring its potential as a therapeutic target to inhibit metastatic progression [199]. NAD-dependent protein deacylase sirtuin-5 (SIRT5) exerts a pleiotropic effect on regulating cancer cell metabolism, possibly through mediating posttranslational modifications (PTMs) of its target substrates [200]. Hu et al. demonstrated that the deletion of SIRT5 enhances tumorigenesis by disrupting glutamine metabolism via aspartate aminotransferase (GOT1). Loss of SIRT5 increases the aggressiveness of PDAC, indicating potential therapeutic strategies aimed at restoring its expression or activity in SIRT5-deficient tumors [201]. Carcinoembryonic antigen-related cell adhesion molecule 6 (CEACAM6) is emerging as a crucial determinant of the malignant phenotype in a range of cancers, including PC [202]. Research indicates that negative CEACAM6 expression is correlated with the absence of lymph node metastases and improved postoperative survival rates [203]. Integrative analysis has shown that CEACAM6 affects several hallmarks of PDAC, including fibrotic reactions, immune regulation, and energy metabolism, making it a promising therapeutic target [204]. To address chemoresistance, Jiang et al. demonstrated that ubiquitin-conjugating enzyme E2 T (UBE2T) promotes pyrimidine biosynthesis and alleviates replication stress, leading to gemcitabine resistance by catalyzing the ubiquitination-dependent degradation of p53 and alleviating the transcriptional repression of RRM1 and RRM2 [205] Combining gemcitabine with a UBE2T inhibitor not only significantly improved long-term survival in spontaneous PC mouse models but also markedly reduced tumor growth in humanized models. Notably, this combination treatment can also inhibit the development of drug-tolerant persisters, increasing the durability and effectiveness of therapy. Together, these findings highlight the multifaceted potential of targeting PGAM1, SIRT5, CEACAM6, and UBE2T in PDAC treatment. However, rigorous validation across diverse cohorts and developmental stages remains essential to translate these targets into clinical practice.

### Therapeutic agents for PC

**Fig. 5:**
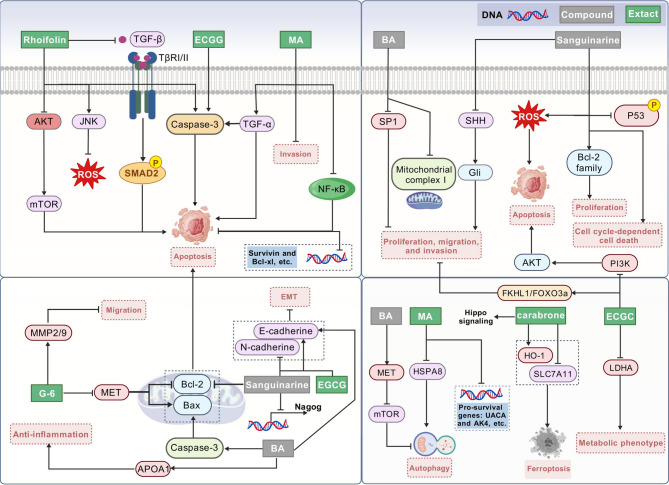
Underlying mechanisms of several potential PC therapeutics via proteomic approaches. On the one hand, for compounds with established antitumor activity but undefined mechanisms in PC, deep-coverage proteomics enables systematic screening of differential protein signatures. This approach facilitates the precise identification of molecular targets and associated mechanistic pathways. On the other hand, for candidate compounds with therapeutic potential in PC with unknown targets, proteomic profiling provides a powerful platform to delineate pharmacodynamic biomarkers and elucidate the underlying mode of action. Some potential drugs and their related mechanisms of action are currently being explored in the context of PDAC and are shown in the figure. “↑” indicates activation, stimulation or promotion, whereas “⊥” indicates inhibition, suppression or decrease. The figure was created with BioGDP.com (https://BioGDP.com)

The development of safer and more effective therapies for PC remains a critical unmet need in oncology. Alongside biomarker discovery, proteomics has emerged as a powerful tool for identifying novel drug candidates and clarifying the mechanisms of action of therapeutic agents in PC. This approach not only accelerates drug development but also provides mechanistic insights to refine existing treatments. Plant-derived compounds, renowned for their low toxicity and multitarget effects, have gained traction in cancer research. Recent advances in quantitative proteomics have enabled researchers to systematically validate the anticancer properties of natural products in PC. By mapping protein interactions and signaling pathways modulated by these compounds, proteomics has revealed novel therapeutic targets and mechanisms, positioning plant-based agents as promising candidates for PDAC drug development. Several natural products have emerged as potential candidates for the development of new drugs to treat PC. We detail their potential mechanisms and emphasize the key molecular targets involved enhancing the therapeutic effect against PC (Fig. 5). By integrating proteomic insights with functional validation, these compounds offer a strategic avenue to overcome drug resistance and improve outcomes in PC patients.

#### Rhoifolin

Rhoifolin is one of the principal components with potent antioxidant and anti-inflammatory activities in total flavonoids derived from Plumula nelumbinis. Moreover, Rhoifolin has potent antiproliferative effects on cancer cell lines [206]. Recent studies have highlighted its anticancer potential in PC cell lines (PANC-1 and ASPC-1), where it inhibits cell proliferation and induces apoptosis by activating the JNK signaling pathway while suppressing the AKT signaling pathway. This compound also downregulates transforming growth factor beta 2 (TGF-β2) and phosphorylated SMAD2. Furthermore, Rhoifolin suppresses cell migration and invasion while enhancing their antioxidant capacity [207]. These findings underscore its role in disrupting critical survival mechanisms in PDAC patients.

#### MA

Maslinic acid (MA) is a dietary pentacyclic triterpene widely distributed in common foods that exhibits broad-spectrum anticancer activity. In PC, MA enhances TNF-α–mediated antitumor effects and suppresses tumor growth by activating caspase-dependent apoptosis and blocking NF-κB signaling. Additionally, MA inhibited the expression of NF-κB-regulated antiapoptotic genes, such as Survivin and Bcl-xl [208]. Furthermore, it downregulates the heat shock protein HSPA8 in Panc-28 cells to inhibit their proliferation and induce autophagy [209]. A proteomic study revealed that MA can inhibit the proliferation, migration, and invasion of PANC-1 and Patu-8988 cells and induce their apoptosis [210]. Mechanistically, MA suppresses the expression of prosurvival genes such as UACA and AK4, highlighting its potential as a multitargeted PDAC therapeutic [210].

#### Sanguinarine

Sanguinarine is a bioactive benzophenanthridine alkaloid found in plants of the Papaveraceae family, such as the bloodroot plant Sanguinaria. This plant alkaloid exhibits strong antibacterial, anti-inflammatory and antioxidant activities [211]. It has also been shown to induce cell death via apoptosis [212]. Specifically, sanguinarine exerts anti-proliferative effects on PC cells (AsPC-1 and BxPC-3) by modulating Bcl-2 family proteins, resulting in G1-phase cell cycle arrest in these cells [213]. Mechanistic studies suggest that sanguinarine exerts its effects through the coordinated regulation of multiple signaling pathways, with potential mediators such as IL33, CUL5, GPS1, and DUSP4 [214] These targets collectively modulate critical cellular processes in pancreatic carcinogenesis. Additionally, sanguinarine treatment downregulates HIF1α and PCNA while increasing the cleavage of PARP and CASP7. Moreover, sanguinarine regulated the EMT of pancreatic CSCs by increasing E-cadherin levels and inhibiting N-cadherin expression [215].

#### G-6

Nardoguaianone L (G-6) is a novel compound derived from the natural product Nardostachys jatamans. Sang et al. compared protein expression in SW1990 cells treated with or without G-6 via quantitative proteomics and revealed that G-6 inhibits colony formation and cell migration while inducing apoptosis, suggesting its therapeutic potential via regulation of the MET/PTEN/TGF-β pathway [216].

#### Carabrone

Carabrone is a natural compound from Carpesium cernuum. Zheng et al. evaluated the cytotoxicity of carabrone in four PC cell lines (SW1990, Capan-2, CFPAC-1, and PANC-1). Proteomic analysis revealed that carabrone inhibits SW1990 cell proliferation and migration by upregulating CSNK1E and downregulating WWTR1, thereby activating the Hippo signaling pathway [217]. Notably, carabrone also induces ferroptosis through the downregulation of SLC7A11 and the upregulation of HO-1 [217]. 

#### BA

Betulinic acid (BA), a pentacyclic triterpene, possesses a wide spectrum of biological and pharmacological properties, particularly its antitumor effects [218]. Chiu et al. reported that BA inhibited the survival of PDAC cells and reduced their migration ability [219]. Proteomic analysis suggested that these effects may be attributed to alterations in mitochondrial function caused by BA, including reduced activity and oxidative phosphorylation of mitochondrial complex I and downregulation of POLRMT and TACO1 expression. Additionally, BA-induced upregulation of APOA1 and downregulation of NLRC4 may explain its anti-inflammatory and antimetastatic properties [219]. In another study, BA suppressed PC in a dose-dependent manner both in vitro and in vivo and inhibited PC by specifically targeting the mTOR-Caspases/Bcl2/Bax apoptotic signaling pathway [220]. Moreover, the combination of BA with mithramycin A can enhance therapeutic efficacy by inhibiting cell proliferation, invasion, and angiogenesis in human PC through the downregulation of SP1 [221]. Similar to sanguinarine, BA can inhibit EMT by increasing the expression of E-cadherin and decreasing the expression of vimentin, thereby suppressing the migration of PDAC cells. A previous study confirmed that BA significantly downregulates lamin B1 in both in vitro cultures and xenograft models of PC [222].

#### Phospholysine

Phosphohistidine inorganic pyrophosphate phosphatase (LHPP) is a protein histidine phosphatase and tumor suppressor, suggesting an oncogenic role for dysregulated histidine phosphorylation [223]. Some studies have revealed that LHPP can inhibit the growth of various cancers [224]. The expression level of LHPP was lower in PDAC tumor tissues than in adjacent nontumor controls. Furthermore, LHPP overexpression significantly inhibited the viability, migration and proliferation of BxPC-3 and PANC-1 cells [223]. Moreover, LHPP overexpression inhibited xenograft tumor growth in vivo. Therefore, LHPP might be considered an important target for delaying the progression of PDA [225].

#### EGCG

Epigallocatechin gallate (EGCG), the primary bioactive polyphenol found in green tea, has emerged as a promising therapeutic agent for PC. Its mechanism primarily involves targeting lactate dehydrogenase A (LDHA), a key enzyme in tumor metabolism that catalyzes the conversion of pyruvate to lactate while regenerating NAD^+ ^ [226]. LDHA is often overexpressed in many cancers and acts as a biomarker for tumor progression and prognosis [227]. Lu et al. demonstrated that EGCG disrupts metabolic flux by downregulating LDHA in HPAF-II cells, which alters the cellular metabolic phenotype and suppresses glycolytic activity, a hallmark of cancer cell survival [228]. Furthermore, the antitumor efficacy of EGCG extends beyond metabolic modulation. In pancreatic xenograft models, EGCG significantly reduces tumor volume, proliferation, angiogenesis, and metastasis [229]. Mechanistically, EGCG inhibits EMT by upregulating E-cadherin and suppressing N-cadherin and Zeb1, thereby reducing metastatic potential. Additionally, it can induce apoptosis via the activation of caspase-3 and exerts a dual regulatory influence on signaling pathways, inhibiting the PI3K/AKT and ERK signaling pathways while simultaneously activating the FKHRL1/FOXO3a pathway [230]. Consequently, EGCG has emerged as a promising potential therapeutic agent against PC.

#### SOM230

SOM230 is a somatostatin analog that has been proposed as an antimetastatic therapy for PDAC. Its ability to remodel the fibrotic and immunosuppressive myeloid stroma may synergize with chemotherapy to improve clinical outcomes, although further validation in clinical trials is warranted [231]. Suzuki et al. used a proteomics method to identify proteins in lymph node metastasis-positive and -negative patients who underwent pancreatectomy. The results revealed that elevated hemopexin expression was correlated with aggressive pathological features, including UICC N2 classification, the lymph node ratio, venous invasion, and lymphatic invasion [232]. While hemopexin has potential as a therapeutic target or diagnostic marker, further mechanistic studies are necessary to clarify its role in tumor invasion and metastasis.

#### Nitroxoline

Nitroxoline, a quinoline derivative, has potent anticancer effects on PC. Veschi et al. demonstrated that nitroxoline reduces PC cell line survival in a dose-dependent manner, affects the cell cycle, and decreases the expression of cell cycle-related proteins, severely affecting the clonal activity of PC cells [233]. Its efficacy has been validated in various animal cancer models [233]. Additionally, proteomic analyses revealed that nitroxoline downregulates the Na/KATPase pump and β-catenin, disrupts cell membrane iron homeostasis, and affects cotranslational membrane targeting, all of which contribute to its anticancer effects [234].

#### Immunotherapy

LOAd703 is a genetically engineered oncolytic adenovirus armed with trimerized CD40L and 4-1BB ligand (4-1BBL). It activates dual immunostimulatory pathways (CD40 and 4-1BB) to enhance antitumor immunity [235]. In PDAC cell lines, LOAd703 demonstrated potent cytotoxicity against cell lines upon infection with oncolytic vectors, surpassing the efficacy of conventional gemcitabine therapy. In xenograft models, this construct markedly reduced the established tumor volume and exhibited potential synergy with gemcitabine. This multifunctional immune activator further modulates stromal components to amplify antitumor immunity [235]. Patient-derived and murine PDAC models revealed the upregulation of NCOA4 and ferritinophagy in PC, which critically support iron metabolism and maintain the stability of iron‒sulfur (Fe‒S) cluster-containing proteins [236]. Targeting NCOA4 delayed tumor growth and prolonged survival, although compensatory iron acquisition pathways developed. Increased ferritinophagy accelerated PDAC tumorigenesis, with a high ferritinophagy expression signature predicting poor prognosis in PDAC patients. These findings emphasize the critical role of iron homeostasis in PDAC autophagy and highlight NCOA4-mediated ferritinophagy as a potential therapeutic target. In another study, Nelson et al. identified USP25 as a key mediator in maintaining PDAC and survival through the regulation of HIF-1α protein stability and metabolic rewiring [237]. Targeting USP25 may be a promising therapeutic strategy for treating PDAC [237].

#### Exosome-based therapeutic strategies

Exosomes can be used as therapeutic agents because of two characteristics. First, exosomes are immunogenic, featuring many tumor-associated antigens (TAAs) on their surface that can trigger an antitumor immune response, leading to immunogenic cell death in tumor cells [238]. For example, previous studies have demonstrated that downregulating the exosomal surface glycoprotein GPC1 can inhibit tumor angiogenesis and metastasis [239]. Moreover, exosomes isolated from the serum of PDAC patients, as well as from KRAS-transformed fibroblasts and PC cells, are highly enriched with the cell survival protein survivin, which effectively suppresses the malignant properties of PC [240]. Modified exosomes, particularly those engineered through genetic recombination, have the potential to act as immunotherapeutic agents. Jang et al. demonstrated that tumor-derived exosomes can activate antitumor immune responses through dendritic cell maturation [241]. Additionally, Zhou et al. developed a drug delivery system based on BM-MSC-derived exosomes [242]. This strategy improves DC maturation, reverses immunosuppression, increases the infiltration of antitumor cytotoxic T lymphocytes, and induces effective innate and adaptive immune responses against PC. Second, certain exosome cargos can inhibit the malignant behaviors of PC cells. Studies have revealed that inhibiting exosome secretion prevents the transformation of PSCs into PC cells [243] restores NK cell activation levels [244] and ameliorates GEM resistance induced by exosomal cargo [245]. Targeting exosome biogenesis or cargo delivery represents a dual strategy to mitigate tumor progression and enhance treatment efficacy.

#### Combined treatment

Zhu et al. utilized proteomic analysis to explore the effects of combining gemcitabine and birinapant in PC cells. This study not only offers insights into drug effects and interactions in various signaling pathways but also provides a foundation for systems mathematical modeling to understand the combined effects of these treatments on PC cells [246]. PP2A is the major serine/threonine phosphatase in cells and maintains a cellular balance by counteracting kinase-driven signaling pathways [247]. In one study, researchers screened PDA cell lines for kinase inhibitors and reported that PP2A was suppressed. They reported that inhibiting mTOR signaling led to increased apoptosis and reduced oncogenic phenotypes both in vitro and in vivo [248]. Therefore, combining PP2A inhibition with chemotherapeutic regimens may enhance the therapeutic effects against PC.

Overall, these sections of the review have comprehensively explored the transformative role of proteomics in PC research, spanning the discovery of diagnostic, prognostic, and predictive biomarkers, therapeutic target identification, and molecular subtyping. Table 2 summarizes key proteomic applications of clinical relevance discussed in this review. Each entry exemplifies how proteomics redefines PDAC management, offering a curated reference for translating omics discoveries into clinical practice. Future efforts should focus on evaluating the clinical value and biological functions of these biomarkers across larger patient cohorts through multi-institutional collaboration and standardized techniques.

**Table 2:** Representative proteomics applications in pancreatic cancer

Potential utilities	Molecules	Samples	References
Early Diagnosis	GPRC5C and EPS8	Serum EVs	[151]
	TIMP1 and LRG1	Plasma	[162]
	AGP1	Serum, tissue	[163]
	GPC1	Serum exosomes serum	[249]
	KIF5B and SFRP2	EVs, TMA	[250]
Differential diagnosis	CA19-9, ApoA-I and TIMP1	Serum	[166]
	LIF	Tissue, plasma	[251]
	ARG2	Pancreatic juice	[252]
Molecular subtyping	3,960-protein signature	Tissue	[253]
	2,311-protein signature	Tissue	[254]
Prognostic assessment	MIF	Plasma exosomes	[157]
	S100A4	Mouse pancreatic organoids	[255]
	YAP1	Tissue	[256]
	24-protein panel	Tissue	[257]
Treatment response prediction	EphA2	Exosomes	[152]
	NDUFB8 and CEMIP2	Tissue	[186]
	PZ, SHBG, VWF and CA19-9	Plasma	[193]
Therapeutic target discovery	MICAL1	Tissue, PC cell lines	[197]
	SIRT5	Tissue, organoids, patient-derived xenografts	[201]
	UBE2T	Organoids, tissue	[205]

*EVs* extracellular vesicles, *TMA* tissue microarray, *PC* pancrreatic cancer

## Pathological mechanism of PC

The pathological mechanisms underlying PC remain only partially understood. Emerging evidence highlights gene mutations, the interplay of immune and metabolic dysregulation within the TME, and aberrant cell signaling pathways as central drivers of PC pathogenesis. Together, these factors drive the transformation of normal pancreatic tissue into malignant tissue, which is characterized by a series of molecular and cellular alterations. Notably, numerous inflammatory molecules involved in the inflammatory response also play important roles in the pathological process of PC, with the interleukin family being particularly critical. As pancreatic tissues and cells transition from normal to cancerous, they exhibit a range of protein abnormalities. Proteomics has significantly advanced our understanding of biological processes. Understanding the structure, function, and interactions of proteins in specific spatiotemporal contexts is crucial for deciphering potential biomarkers, elucidating various signaling pathways, gaining an in-depth understanding of tissue pathology, and designing targeted therapies.

### Carcinogenesis and progression of PC

**Fig. 6:**
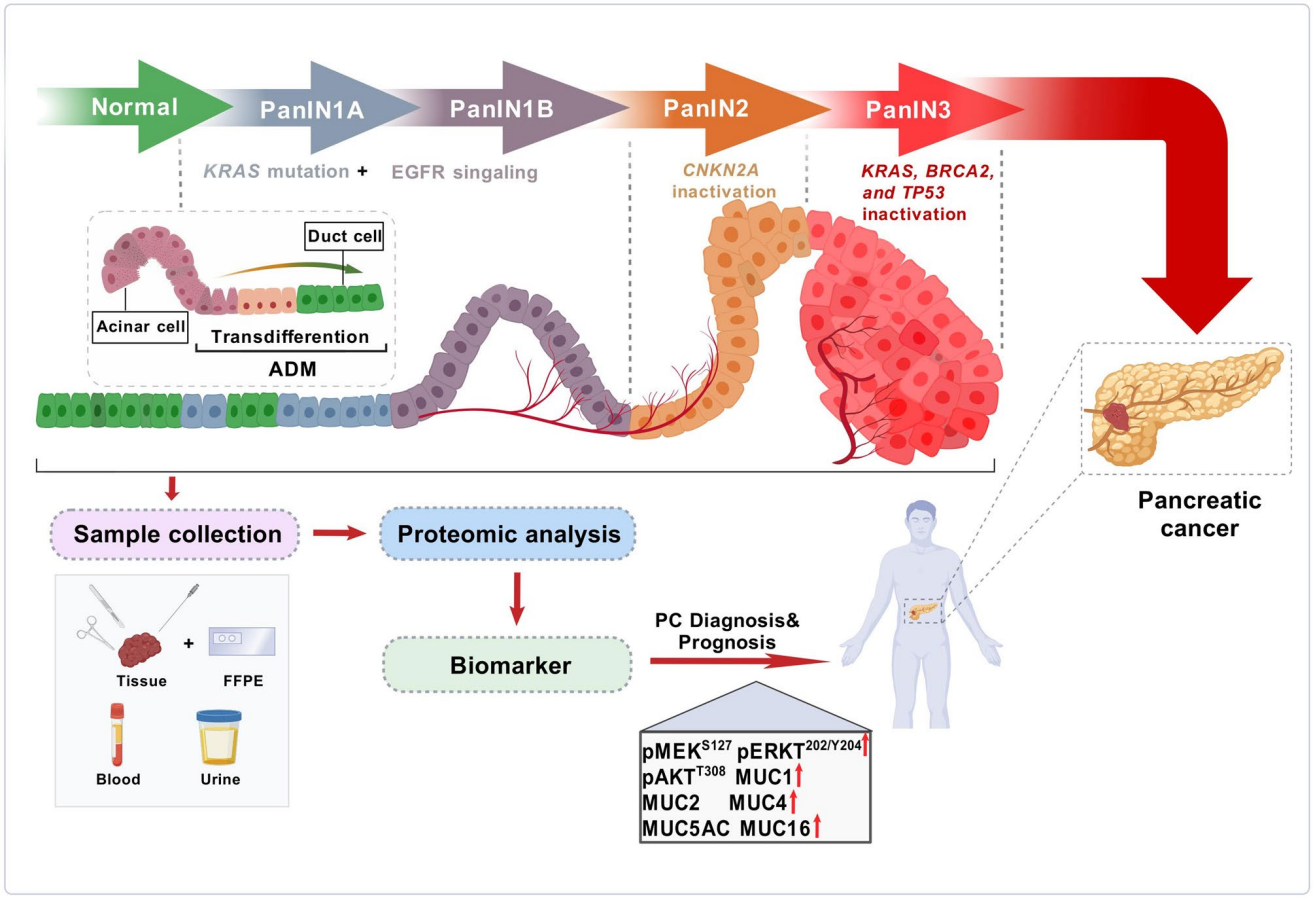
Mechanisms for initiating PanINs and their progression to PDAC. Oncogenic KRAS induces neoplastic transformation of pancreatic acinar cells through acinar-to-ductal metaplasia (ADM), an actin-based morphogenetic process, and drives PDAC. During the progression from low-grade to high-grade precursor lesions, inactivation of the tumor suppressor gene CDKN2A is observed. As these precursor lesions progress to PDAC, concomitant loss or inactivation mutations in the critical tumor suppressor genes BRCA2 or TP53 occur. Longitudinal collection of stage-stratified patient samples (e.g., tissue or liquid biopsies) during this progression, coupled with proteomic approaches, may enable systematic screening of biomarkers possessing dual early diagnostic and prognostic potential, thereby enhancing prognostic stratification capabilities in PDAC management. FFPE, formalin-fixed paraffin-embedded. The figure was created with BioGDP.com (https://BioGDP.com)

PDAC originates from noninvasive precursor lesions, with three main types (pancreatic intraepithelial neoplasms (PanINs), intraductal papillary mucinous neoplasms (IPMNs), and mucinous cystic neoplasms (MCNs)) progressing to PC [258]. It is believed that intraductal precursor cells undergo a progressive neoplastic evolution from low-grade to high-grade lesions, characterized by progressively increasing cellular morphological abnormalities and genetic aberrations, ultimately developing into invasive adenocarcinoma. Specifically, PanINs are associated with the accumulation of genetic alterations that drive histologic progression from PanIN-1 (hyperplasia) to PanIN-2 (atypia), PanIN-3 (carcinoma-in situ) and invasive ductal adenocarcinoma (Fig. 6). One of the key components of the pancreas’s response to injury and repair is the transient transdifferentiation of acinar cells into an embryonic ductal state. This process reduces the demand for protein production while promoting cell proliferation. As these duct-like cells prepare to fill the necrotic acini, they restore exocrine function upon redifferentiation. Failure to redifferentiate back into acinar cells is considered to represent the first stage of the tumor cascade, forming PanIN, and ultimately leading to invasive cancer [259]. Based on current findings, characterizing of the dynamic protein molecular changes across normal pancreatic tissue, benign precursor lesions, and various stages of PC is crucial for enhancing the clinical management of pancreatic diseases.

PanINs are the most common precursor lesions for PDAC (approximately 85-90%) and have been extensively characterized, while the remaining 10–15% are derived from cystic precursor lesions [36]. Clinically, PanINs are extremely minuscule and usually asymptomatic, making them difficult to detect with current imaging technologies. Unfortunately, they are usually identified only in the later stages of the disease, when a diagnosis can finally be established. The incidence of PanINs is greater among males and older individuals [260]. The stepwise progression of PDAC involves sequential alterations in key driver genes. Oncogenic KRAS mutations initiate the malignant transformation of normal ductal epithelium and achieve near-universal prevalence in low-grade PanINs. As the condition progresses to high-grade PanINs, tumor suppressor genes become inactivated, including the dysregulation of *CDKN2A* and the loss of *TP53* and *SMAD4* [261] These findings establish definitive molecular signatures of invasive malignancy (Fig. 6). Different protein molecules involved in various signaling pathways play important roles in pathological mechanisms at this stage. Analysis of human samples revealed that Wnt3a as a significant protein biomarker for the canonical Wnt/β-catenin signaling pathway, which is necessary for the transition from early to advanced PanINs [262]. Protein kinase D1 (PKD1) operates downstream of TGFα and Kras, facilitating the formation of ductal structures by activating the Notch pathway. It is sufficient to drive the reprogramming process toward a ductal phenotype and progression to PanINs [263]. In a mouse model of PC, the NLRP3-regulated IL-18 signaling pathway was activated in cells, leading to an accumulation of eosinophils that contributed to the development of PanIN. Key proteins include NLRP3, IL18, pNLRP3, Casp1, IL1β, MRC1, and CD11b [264]. A study involving serum samples from 187 PDAC patients, 93 individuals with benign pancreatic conditions, and 169 healthy subjects confirmed that TIMP1 (AUC = 0.949) and LRG1 (AUC = 0.887) are promising biomarkers for the early detection of PDAC [265]. The integration of multiomics approaches can deepen our understanding of PC development and progression. Zhou et al. utilized single-cell/nucleus RNA sequencing, bulk-proteogenomic, spatial transcriptomic and cellular imaging to examine 83 spatial samples from 31 patients. These findings revealed that subsets of tumor cells are characterized by proliferation, KRAS signaling, cellular stress, and epithelial–stromal transition, shedding light on the progression from normal cells to precancerous cells, and eventually to PDAC. Gaining insights into these cellular details, especially the molecular pathways involved in tumor progression, could help identify potential therapeutic targets and support the development of innovative therapies for PC [266].

IPMNs represent another type of precursor lesion to PC. In the case of IPMNs, alongside KRAS mutations, researchers have identified activating mutations in the gene encoding the G protein alpha subunit gas (GNAS), as well as the loss of function of the tumor suppressor gene RING-type E3 ubiquitin ligase (RNF43) as contributing factors [267]. A proteomics study revealed 11 protein biomarkers associated with IPMNs with high confidence in 184 plasma samples [268]. Another study reported that 364 proteins were differentially expressed in IPMNs in pancreatic cyst fluid [269]. TFF2, a member of the trefoil factor family (TFF), is primarily co-secreted with mucin-6 by gastric mucous neck cells, pyloric gland cells, and duodenal Brunner’s glands. Research suggests that the monomeric form of TFF2 may exert receptor-mediated protective effects on the pancreatic ductal epithelium. Znalesniak et al. revealed heterogeneous molecular forms of TFF2 through proteomics methods and demonstrated that a deficiency in TFF2 is associated with the accelerated development of PIMNs. This finding highlights its potential tumor-suppressive role in maintaining ductal homeostasis [270]. A Finnish study employed label-free proteomics to distinguish between various stages of IPMNs and healthy pancreas serum. Research has identified a panel of protein biomarkers associated with the progression of IPMNs from low-grade to invasive pancreatic mucinous carcinoma (IPMC). Key biomarkers include kininogen-1, apolipoproteins, complement proteins, and retinol-binding protein-4 [271].

MCNs are among the rare precursor lesions of PC. Recent advancements in preoperative imaging techniques have facilitated their detection. However, there has been limited research on MCNs utilizing proteomic methods. Previous studies have indicated that the mucins MUC5AC and MUC2 are present in noninvasive MCNs, while the expression of MUC1 is linked to the development of invasive characteristics [272]. Additionally, recent findings have shown that the progesterone receptor (PR) is abnormally expressed in MCNs containing ovarian-type stroma. These discoveries suggest that the PR may serve as a valuable diagnostic protein biomarker for MCNs [273].

### Tumorigenesis, invasion and metastasis of PC

Tumor cell proliferation and apoptosis occur throughout the entire process of tumorigenesis and development, which provides key insights into the pathogenesis of tumors, as well as their invasion and metastasis. Additionally, the proteins involved in regulating cell proliferation and apoptosis can serve as important targets for cancer treatment. When these relevant proteins are altered, they can either promote or inhibit tumor formation.

**Fig. 7:**
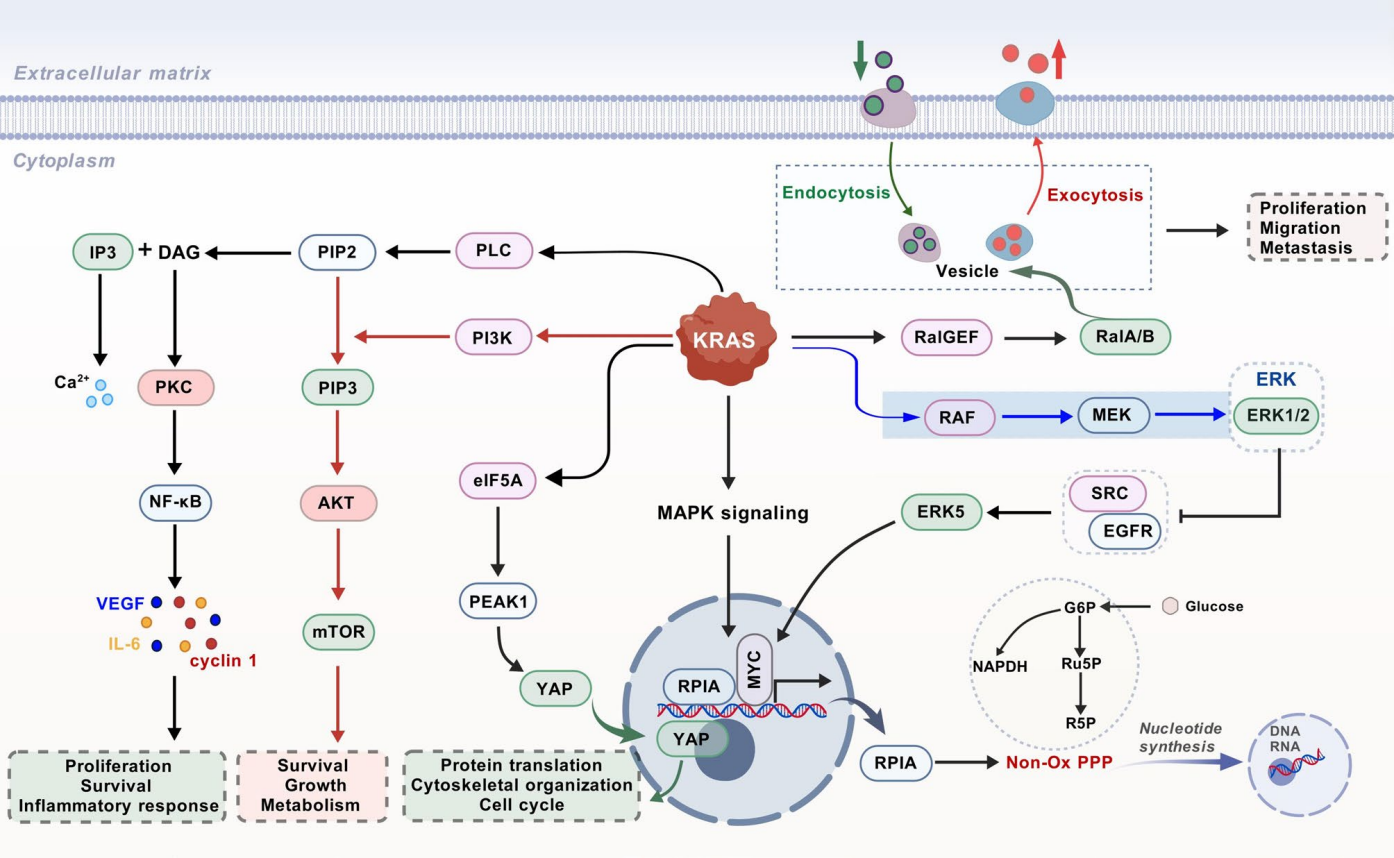
Some KRAS signaling pathways mediating PC mentioned in this review. KRAS is activated through the binding of ligands and receptors and transmits signals to multiple downstream signaling pathways. On the one hand, it affects endocytosis, exocytosis and the distribution of receptors on the cell membrane. On the other hand, it regulates the expression of various genes in the nucleus, playing a role in multiple physiological processes in tumor cells. “↑” indicates activation, stimulation or promotion, whereas “⊥” indicates inhibition, suppression or decrease. Abbreviations: PLC, phospholipase C; PI3K, phosphoinositide 3-kinase; PIP3, phosphatidylinositol 3,4,5-trisphosphate; NF-κB, nuclear factor kappa-B; mTOR, mammalian target of rapamycin; eIF5A, eukaryotic translation initiation factor 5 A; PEAK1, pseudopodium enriched atypical kinase 1; YAP, myelocytomatosis oncogene; MEK, mitogen-activated protein kinase/extracellular signal-regulated kinase; RAF, Proto-Oncogene Serine/Threonine-Protein Kinase; ERK, extracellular regulated kinase; RPIA, ribose 5-phosphate isomerase A; Non-Ox PPP, the non-oxidative oxidative branch of the pentose phosphate pathway; Ru5P, ribulose 5-phosphate; G6P, glucose-6-phosphatase G-6-pase. The figure was created with BioGDP.com (https://BioGDP.com)

#### Gene mutation and signaling pathways

**Fig. 8:**
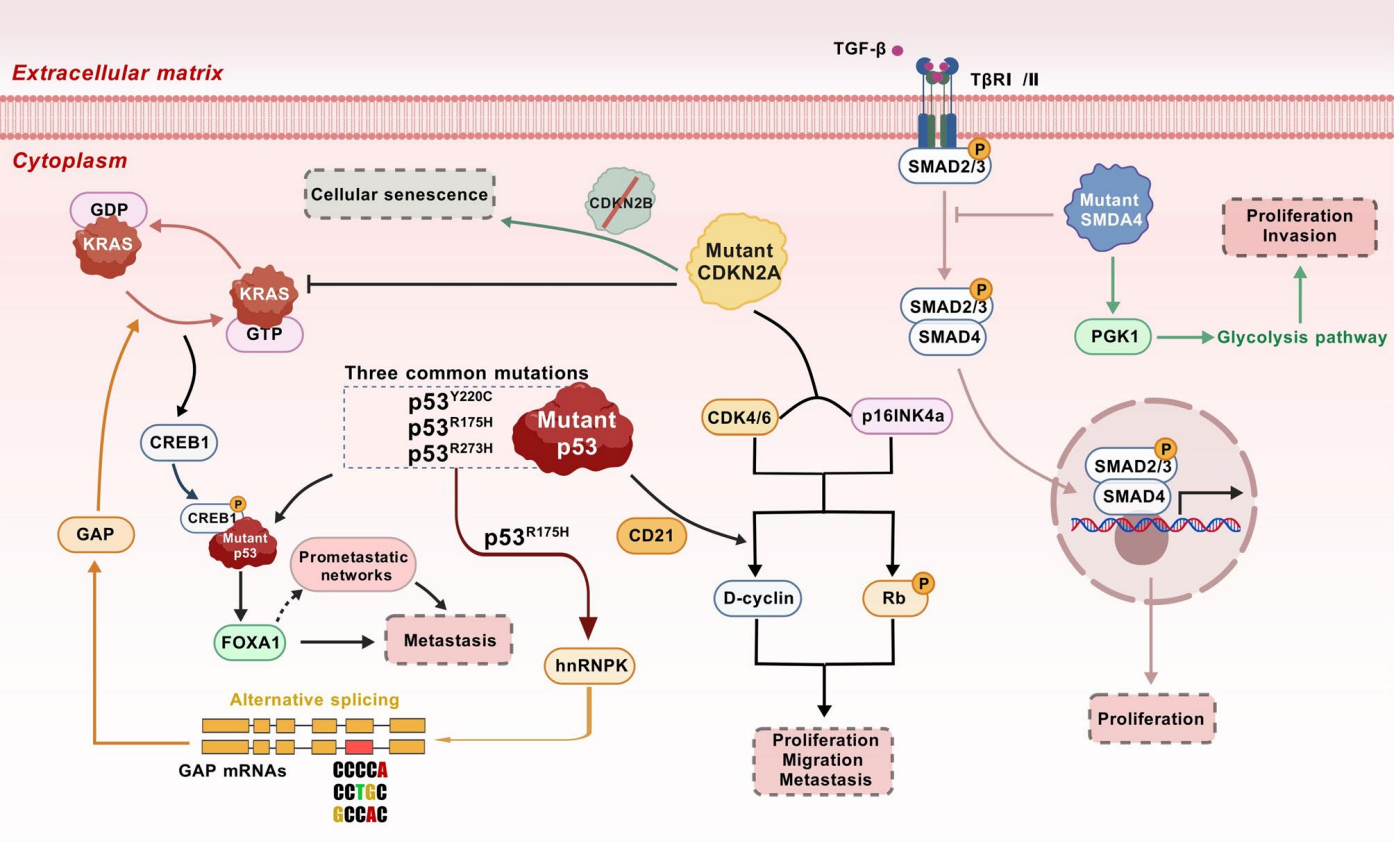
Some p53, CDKN2A, and SMAD4 signaling pathways mediating PC mentioned in this review. P53 is a crucial tumor suppressor gene. The figure illustrates its three most common mutation forms. It influences the metabolism, metastasis, and proliferation of tumor cells through downstream signaling molecules. It interacts with the KRAS signaling pathway through hnRNPK and CREB and with CDKN2A via CD21. Mutated CDKN2A generates the p16 biomarker, which affects genomic stability and cell proliferation through cyclins and Rb. SMAD4 affects cell proliferation by regulating the expression of genes related to TGF-β in the nucleus. In the cytoplasm, it impacts the cell glycolysis process via PGK1 and influences cell proliferation and tumor growth. “↑” indicates activation, stimulation or promotion, whereas “⊥” indicates inhibition, suppression or decrease. Abbreviations: TGF-β, transforming growth factor β; SMAD, small mothers against decapentaplegic homolog; PGK1, phosphoglycerate kinase 1; CDKN2A, cyclin-dependent kinase inhibitor 2 A; CDK, cyclin-dependent kinase; CREB, cAMP response element binding protein; FOXA1, forkhead box A1; hnRNPK, heterogeneous nuclear ribonucleoprotein K; Rb, retinoblastoma protein. The figure was created with BioGDP.com (https://BioGDP.com)

The malignant progression of PDAC, from premalignant lesions to invasive and metastatic disease, is driven by the activation of proto-oncogenes and inactivation of tumor suppressor genes. Tumor genetics studies have revealed that somatic mutations in *KRAS* (> 90% of cases), *TP53* (60-70%), *SMAD4* (30-40%), and *CDKN2A* (30-40%) are characteristic molecular genetic features of the vast majority of PC [274]. Whole-genome analyses further revealed novel candidate drivers of PC, including *KDM6A*, *PREX2*, DNA maintenance genes (*BRCA1*, *BRCA2*, and *PALB2*), and druggable oncogenes (*ERBB*2, *MET*, *FGFR1*, *CDK6*, *PIK3R3*, and *PIK3CA*) [275]. These genetic alterations are central to PDAC pathogenesis and drive the dysregulation of tumor-related proteins and signaling pathways. Therefore, gene mutations are the root cause of disease occurrence, making them a fundamental focus in medical research. In recent years, proteomics research has revealed the complex alterations in signaling pathways triggered by these genetic mutations, providing new perspectives for the molecular mechanisms underlying PDAC. For example, the oncogenic *KRAS* mutation is a pivotal early event in PDAC, that occurs in the early stages of pancreatic carcinogenesis and is found in common precursor lesions such as PanINs and IPMNs. Some studies have shown that increased *Kras*^*G12D*^ gene expression drives constitutive Kras activation, functions as a molecular switch to activate various intracellular signaling pathways and transcription factors, and promotes proliferation, migration, and survival (Fig. 7) [276]. *KRAS* critically regulates the optimal mitogen-activated protein kinase (MAPK) signaling pathway [277]. Mutations in *KRAS* activate this pathway, resulting in the upregulation of MYC and the transcription of RPIA. This process increases flux into the non-oxidative branch of the pentose phosphate pathway (PPP) and further increases nucleotide biosynthesis and promotes tumor progression [278]. Moreover, *KRAS* mutations can lead to persistent activation of RalA/B through the RalGEF/Ral signaling pathway, regulating endocytosis and exocytosis and affecting the distribution of cell-surface receptors and growth factors, ultimately promoting the proliferation, migration, and metastasis of PC cells [279]. Conventional theories posit that the PI3K-AKT-mTORC1 signaling pathway governs *KRAS* mutation-driven PC growth, which regulates the survival, growth, and metabolism of tumorous cells in PDAC [280]. KARS recruits and activates PLC and hydrolyzes PIP2 to generate DAG and IP3. The latter leads to an increase in intracellular Ca^2+^ and the activation of protein kinase C and downstream NF-κB. NF-κB then transcribes a series of proteins (cyclin 1, VEGF, and IL-6). This process affects cell proliferation, survival, and the inflammatory response, which in turn promotes tumorigenesis and tumor development [281]. Gene expression can be influenced by various risk factors for PC. Clement et al. evaluated the combined effects of alcohol exposure and *KRAS* mutation status on the transcriptome and proteome of human pancreatic nestin-expressing (HPNE) cell models and determined that alcohol preferentially induces and increases the proliferative potential of HPNE cells expressing mutant *KRAS* [282]. Previous studies reported that mutant *KRAS* regulates MYC via ERK1/2-dependent mechanisms in PDAC. ERK1/2 blockade activates a compensatory EGFR-SRC-ERK5 cascade that stabilizes MYC, and combined ERK1/2 and ERK5 inhibition promotes synergistic loss of MYC and suppresses PDAC growth [283]. Recent research presents a distinct perspective suggesting that the oncogenicity of *KRAS* mutations relies primarily on the sustained activation of the RAF-MEK-ERK MAPK signaling pathway [284]. J.A. Klomp et al. established a *KRAS*-regulated gene transcriptome in *KRAS*-mutant PC. They reported that the *KRAS* mutant transcriptome is regulated primarily through the activation of the ERK mitogen-activated protein kinase cascade, which serves as a key factor contributing to *KRAS* inhibitor resistance. They discovered that in cancers driven by *KRAS* mutations, approximately 80% of protein expression changes are regulated at the transcriptional level, alongside other post-transcriptional mechanisms, such as the phosphorylation of ERK proteins. Additionally, ERK proteins can influence the expression levels of hundreds of genes. The identified *KRAS*-ERK gene signature includes the upregulation of 278 cell cycle-related genes, which can help predict the therapeutic response of patients to *KRAS* inhibitors. Another study emphasized the protein phosphorylation signature driven by *KRAS* mutations, identifying a total of 2,123 ERK-dependent phosphorylated proteins, 67% of which were previously thought to be unrelated to ERK. This study constructed a protein kinase signaling network centered on ERK-dependent phosphoproteomics [284]. Integrating of data from the Cancer Dependency Map revealed that 17% of the ERK phosphoproteome is critical for PDAC growth, confirming that ERK regulates the functions of cyclin-dependent kinases and the RAS homolog guanosine triphosphatase (RHO GTPase). Moreover, in PDAC, mutant *KRAS* upregulates the translation initiation factor eukaryotic translation initiation factor 5 A (eIF5A) and the focal adhesion kinase PEAK1. This eIF5A-PEAK1 signaling pathway promotes the expression of YAP, which mediates integrin and growth factor signaling in TME [285]. Strnadel et al. reported that eIF5A-PEAK1-YAP signaling contributes to PDAC development by regulating an STF program that is associated with increased tumorigenicity. The major pathways controlled by PEAK1 include protein translation, cytoskeletal organization, and cell cycle regulation [286]. Moreover, beyond KRAS, key mutations in TP53, SMAD4, and CDKN2A cooperatively drive PDAC tumorigenesis, metabolic reprogramming, metastasis, and therapy resistance (Fig. 8). *TP53* mutations represent another highly prevalent genetic alteration in PDAC [287]. Tong et al. demonstrated that *TP53* mutations are associated a poor prognosis in young PDAC patients because of increased CDK4-mediated cell proliferation [288]. The p53 tumor suppressor protein, a master regulator of cell cycle arrest, senescence and apoptosis, is frequently mutated in PDAC, with the p53^Y220C^ variant occurring at a high frequency (> 100,000 annual cancer cases) [289]. In a recent study, Lakshay et al. reported that upon curcumin treatment, almost 227 proteins were dysregulated, with the majority of them being transcriptional targets of *TP53*. Label-free proteomics analysis indicated that curcumin can rescue mutant p53^Y220C^ and mediate apoptosis in PC cells [290]. Polireddy et al. reported that the mutant *TP53*^R175H^ stabilizes HSP70 to initiate tumorigenesis, whereas the mutant p53-driven secretome promote EMT and the expression of potential PC biomarkers [291]. Critically, mutant p53 reinforces its oncogenic impact by upregulating the splicing regulator hnRNPK [292]. This promotes the inclusion of polyC-rich exons within GTPase-activating proteins (GAPs), which are negative regulators of the RAS. As a result, the mutant p53-enriched GAP isoforms lose membrane association, failing to inactivate RAS and thereby stabilizing the GTP-bound KRAS^G12D^. Oncogenic *KRAS* effectors subsequently activate CREB1. Phosphorylated CREB1 interacts with mutant p53, leading to the upregulation of the transcription factor FOXA1. Its overexpression promotes metastasis and hyperactivates several prometastatic networks, ultimately driving PDAC metastasis [293]. SMAD Family Member 4 (*SMAD4*) mutations are found in advanced pancreatic intraepithelial neoplasia and invasive PDAC. It is a critical mediator of TGF-β signaling [294]. Typically, the TGF-β/SMAD4 signaling pathway regulates cell growth by promoting cell cycle arrest and apoptosis. However, phosphorylated SMAD4 protein can attenuate this tumor-suppressive function and accelerate pancreatic tumorigenesis [295]. Specifically, ALK can phosphorylate SMAD4 at Tyr95 to suppress pancreatic tumor responses [296]. Besides, *SMAD4* deficiency induces the upregulation of the glycolytic enzyme PGK1 in PDAC, which enhances tumor glycolytic activity and influences tumor progression and invasion [297]. In murine models of PDAC with *Kras* and *Tp53* mutations, *Smad4* functionally interacts with *Tp53* and *Kras*. Its inactivation reduces metastatic potential but increases cancer cell proliferation compared to the heterozygous deletion of *Smad4* alone [298]. Moreover, combined *Tp53*/*Smad4* loss in *Kras* wild-type murine models can induce spontaneous pancreatic tumorigenesis independent of oncogenic *Kras* mutations, yielding tumors with distinct histopathology [299]. The mutational loss of *CDKN2A* tumor suppressor function complements *KRAS* activation by decreasing p16INK4a binding to CDK4/6, which leads to increased D-cyclin activation and retinoblastoma protein (Rb) phosphorylation, thus promoting PC occurrence and malignant growth [300]. In PDAC, the CDKN2A protein facilitates genomic alterations that mediate tropism and metastasis, whereas *CDKN2B* deletion works alongside *CDKN2A*, induces cellular senescence and protects against *KRAS*-mediated transformation [301, 302]. Clinically, monitoring relevant protein expression levels in CDKN2A/p16 pathogenic variant carriers enabled earlier case detection and improved resection rates and survival compared with those of PDAC patients without surveillance [303]. Additionally, the loss of *CDKN2A* results in a CDK4/6 inhibitor that bypasses RB-mediated cell cycle suppression when combined with mTOR signaling pathway proteins, suggesting potential treatment options for PDAC [304]. Lysine-specific demethylase 6 A (KDM6A/UTX), a frequently mutated tumor suppressor and H3K27me2/3 demethylase in PC, influences PDAC cell identity, tumor sphere formation, migration, and invasion across multiple cell lines [305, 306]. In SPNs of the pancreas, reduced KDM2A expression is notably present [307]. KDM2A acts as an epigenetic regulator of mTORC1 signaling, and KDM2A-deficient pancreatic tumors show increased sensitivity to mTORC1 inhibition [308].

Recent studies have identified additional mutations and signaling alterations that are critical for the progression of PC. One notable mutation is in phosphatidylinositol-3,4,5-triphosphate-dependent rac exchange factor 2 (PREX2), a protein that acts as a guanine nucleotide exchanger for Rac1. This protein is significantly mutated in PDAC, leading to increased PREX2 activity. This heightened activity activates the PI3K/Akt signaling pathway and decreases the number of negative regulators of the cell cycle, which in turn promotes cell proliferation and contributes to the carcinogenesis of pancreatic tissue [309]. Additionally, the breast cancer susceptibility gene (*BRCA*) crucially mediates DNA double-strand break repair via homologous recombination (HR) [310]. Mutations in *BRCA* remodel the PC stromal microenvironment by activating HSF1 signaling-associated proteins, which drive fibroblast transcription into cancer-associated fibroblasts [311]. Another gene involved in HR is serine/threonine kinase (*ATM*), which is the most frequently mutated DNA damage response gene in PDAC and plays essential roles in DNA double-strand break repair, cell cycle control, and apoptosis [312]. Although mismatch-repair protein mutL protein homolog 1 (*MLH1*) mutations are rare in PC, recent studies have linked MLH1 expression levels to patient survival [313]. Among the various signaling pathways influenced by genetic variants, proteomics has emerged as a comprehensive method for detecting associated changes at the proteome level.

#### Epithelial‒mesenchymal transition

Epithelial‒mesenchymal transition (EMT) is associated with three steps in the development of PC: invasion, dissemination, and metastasis [314]. A variety of protein biomarkers have been used to assess EMT [315]. Recently, Swietlik et al. used proteomics and secretomics to uncover aberrant signaling in PDAC, identifying over 10,000 cancer cell-associated proteins that are linked to macrophage polarization and the tumor stromal composition [316]. Additionally, Low et al. identified the EMT state in PC, revealing that the S100 family is crucial in shaping the morphology of solid organoids associated with exogenous TGFβ1 [255] In PDAC, epithelial cells lose their polarity and acquire fibroblast-like mesenchymal traits during EMT. This transition is marked by a switch in cadherin subtypes from E-cadherin to N-cadherin [317]. E-cadherin is an important protein in many signaling pathways, with other proteins influencing PC through E-cadherin-associated signaling pathways. For example, the MIST1 protein has been shown to reverse EMT by regulating the Snail/E-cadherin pathway, thereby reducing the tumorigenicity of PC cells [318]. Additionally, HIF-2α promotes EMT by regulating TWIST2 binding to E-cadherin in PC [319]. Over the past two decades, the EMT and transcription factor families have been considered to include the ZEB, SNAIL and TWIST families, which are involved in cellular adhesion, polarity and cytoskeleton organization [320]. Zinc finger E-box-binding protein (ZEB) is a necessary transcription factor during embryonic development and is related to biological processes such as tumor invasion and metastasis, tumor stem cell stemness maintenance, and tumor angiogenesis. ZEB1 accelerates cell carcinogenesis by promoting inflammation and inhibiting DNA repair glycosylases in epithelial cells [321]. It is a key factor for cell plasticity and enhances metastasis in PC [322]. ZEB2 always binds to different RNA molecules and plays an important role in the progression of pancreatic tumors. Gao et al. reported that the lncRNA ZEB2-AS1 can promote pancreatic cell carcinogenesis and tumor invasion through regulating the miR-204/HMGB1 axis [323]. A recent cell experiment involving MIA PaCa-2 cells transfected with a miR-192-5p mimic revealed the downregulation of ZEB2 expression. These findings suggest that miR-192-5p may function as a tumor suppressor and has high potential as a diagnostic and prognostic biomarker for PDAC [324]. As a member of the zinc finger protein superfamily, SNAIL promotes EMT and the degradation of E-cadherin. It is highly expressed in various tumor tissues and contributes to tumor invasion and metastasis. In PC, low levels of the polarity protein AF6 lead to increased expression of the SNAIL gene, making the SNAIL-associated signaling axis a promising therapeutic target [325]. Furthermore, SNAIL recruits Ring1B to mediate transcriptional repression and cell migration in PC cells [326]. Additionally, SNAIL bypasses senescence and regulates cell cycle progression, promoting EMT during pancreatic carcinogenesis [327]. SLUG is another landmark protein involved in EMT in PC and has been identified as a key factor in both resistance to MEK1/2 inhibition and increased metastasis [328]. Relevant studies have also investigated the role of TWIST in regulating E-cadherin transcription and p16 expression, revealing that HIF-TWIST-polycomb signaling participates in the development and metastasis of PC [329]. Above all, EMT-TF-associated proteins are closely related to the process of epithelial and stromal transformation in PC. Further analysis via proteomic technology will enhance our understanding of the pathological mechanisms underlying PC.

#### Invasion and metastasis of PC

PC is recognized as a highly aggressive malignancy with a strong propensity for metastasis. Most patients are diagnosed at advanced metastatic stages, which contributes to their poor prognosis. A deeper understanding of the mechanisms underlying metastatic progression is critical for developing more effective therapeutic strategies [330]. PC often metastasizes early and extensively to sites such as the liver, peritoneum, pelvic region, bones, lungs, and brain, along with corresponding lymph node metastases. Proteomic technologies have emerged as powerful tools for identifying proteins linked to tumor invasion and metastasis. Lumican, a member of the class II small leucine-rich proteoglycan family, regulates collagen fiber assembly and diameter in the extracellular matrix of various tissues [331]. In PDAC, the expression of lumican in the stroma is linked to tumor invasion and a poor prognosis [332]. Yamamoto et al. conducted a proteomic analysis to examine how lumican regulates cell growth and invasion in PDAC, and 24 candidate proteins were associated with cell apoptosis, invasion, and growth [333]. Tung et al. reported that galectin-1 (Gal1) is linked to PC progression and lymph node metastasis, suggesting its potential as a prognostic marker for PC [334]. Gal1 is expressed primarily in activated pancreatic stellate cells and drives cancer cell invasion and metastasis via the NF-kB signaling pathway [335]. These findings position Gal1 as a promoter of Hedgehog signaling in PDAC and a target for microenvironment-based therapies [336]. Additionally, the Ras GTPase family member Ran plays critical roles in cancer cellular processes. Functional studies have shown that Ran is regulated by the androgen receptor (AR) and CXCR4, influencing invasion and metastasis in PC [337]. Proteomic analyses have further identified the Rab43 GTPase-activating protein as a downstream target of Akt signaling in PC [338]. A membrane proteomic study using LC‒MS/MS compared metastatic AsPC-1 cells with primary BxPC-3 cells, revealing the presence of cadherins (protocadherin-16, protocadherin alpha-12) and alpha-2 catenin in BxPC-3 cells, but not in AsPC-1 cells. These findings suggest that metastatic cells have impaired cell adhesion [339]. Another proteomic analysis compared the secretome of Panc-1 cancer stem-like cells (Panc1 CSCs) with that of their parental cell lines, identifying proteins from various physiological pathways as well as CSC-secreted proteins involved in the metastasis and invasion of PC [340]. These studies underscore the pivotal role of MS-based proteomics in identifying key proteins that drive the invasion and metastasis of PC.

#### Metabolic alterations in PC

Metabolic reprogramming is a hallmark of cancer and is driven by mutations in oncogenes and tumor suppressor genes [341]. In many cancer cells, glycolysis is highly elevated to meet their increased energy, biosynthetic, and redox requirements [341]. Proteomics has emerged as a powerful tool for uncovering metabolic alterations in PC, offering insights into its invasion and progression. Additionally, proteomics has identified various protein biomarkers associated with PC metabolism. One notable example is redox factor-1 (Ref-1), a redox signaling protein that regulates the oxidation‒reduction balance of transcription factors such as HIF-1α, STAT3, and NF-κB, thereby increasing their DNA-binding activity [342]. Proteomic results have revealed that inhibiting Ref-1 and its associated signaling pathways disrupts PC cell metabolism by causing TCA cycle dysfunction [343]. Markus et al. identified a novel ATR activator, Ewing’s tumor-associated antigen 1 (ETAA1), which is indispensable for maintaining genome integrity [344]. Dysregulation of ETAA1 may contribute to the development of PC [345]. Additionally, mitochondrial uncoupling protein 2 (UCP2) has been shown to promote a metabolic shift in PC cells from mitochondrial oxidative phosphorylation (mtOXPHOS) to glycolysis [346]. This study also revealed novel mechanisms by which UCP2 enhances cancer cell proliferation while promoting this metabolic transition [347]. Furthermore, UCP2 mediates aspartic acid transport, participates in glutamine metabolism regulated by *KRAS*, and supports the growth of PC cells [348]. A quantitative metabolomics study revealed that pancreatic stellate cells (PSCs) critically contribute to PDAC metabolic reprogramming by supporting the cancer cell tricarboxylic acid (TCA) cycle and lipid biosynthesis through autophagy-dependent alanine secretion [349]. However, the regulatory role of amino acid transporters in this intercellular metabolic crosstalk remains elusive. To address this, isotopic tracer-based metabolic flux analysis has demonstrated that PSCs preferentially secrete alanine, whereas pancreatic cancer cells (PCCs) exhibit marked alanine uptake [350]. Additionally, TMT-labeled quantitative proteomic profiling revealed selective overexpression of the neutral amino acid transporter SLC1A4 in PSCs (secretory side) and SLC38A2 in PCCs (absorptive side). Mechanistically, SLC1A4 cooperates with passive transporters in PSCs to maintain microenvironmental alanine pools, whereas PCCs upregulate SLC38A2 for metabolic hijacking. Functional validation revealed that SLC38A2 inhibition suppressed tumor progression in subcutaneous and orthotopic models. To delineate CD9-mediated protumorigenic mechanisms in PDAC, Wang et al. identified the glutamine transporter ASCT2 (SLC1A5) as the top CD9 interactor [351]. CD9 enhances glutamine uptake in PCCs by promoting ASCT2 plasma membrane localization. CD9 depletion synergized with ASCT2 inhibition to suppress PDAC organoid growth and prolong survival in KPC mice, highlighting the therapeutic potential of targeting the CD9‒ASCT2 axis. In summary, proteomics has identified numerous proteins associated with metabolic alterations in PC, offering valuable insights for disease diagnosis and treatment strategies.

#### VEGF signaling pathway and intratumoral angiogenesis

The generation and maintenance of a tumor vascular network are essential for tumor growth, as it serves as a pathway for tumor cell metastasis. Tumor angiogenesis is an important condition for tumor development, invasion and metastasis. Hornerin, an S100 fusion protein, is highly expressed in the endothelium of PC independent of vascular endothelial growth factor (VEGF) [352]. Gutknecht et al. noted that endothelial cell hormones influence vessel characteristics typical of the tumor vasculature, but they did not explore the role of hornerin in other cell types within the tumor microenvironment [353]. In PDAC, targeting angiogenesis remains a viable therapeutic strategy, as high levels of VEGF, PDGF, FGF, and their receptors are present. These factors mediate signaling through VEGFR1/2/3, FGFR1/2/3 and PDGFRɑ/β, which is associated with poor outcomes in PDAC patients [354]. However, recent animal experiments indicate that antiangiogenic therapies offer only short-term benefits and often lead to tumor recurrence within months. The efficacy of angiogenesis inhibitors that target the VEGF/VEGFR pathway relies on the promotion of vascular growth and the immunostimulatory chemokine CXCL14, as demonstrated in a mouse model of pancreatic neuroendocrine tumors [355]. Tumors initiate angiogenesis and immunosuppression by activating PI3K signaling in all CD11b + cells, which makes them resistant to VEGF/VEGFR inhibition. Adaptive resistance is associated with an increase in Gr1 + CD11b + cells; however, simply targeting Gr1 + cells does not increase sensitivity to angiogenic blockade [356]. Nectin-4, a member of the nectin family, is expressed in the embryo and placenta. Nishiwada et al. reported that nectin-4 is associated with tumor proliferation, angiogenesis, and immunity in PC [357]. Both human studies and animal experiments indicate that VEFs and intratumoral angiogenesis are important for PC progression, suggesting that these factors can be further optimized as drug therapeutic targets.

#### Tumor microenvironment

The tumor microenvironment (TME) comprises surrounding blood vessels, cancer-associated fibroblasts (CAFs), tumor cells, stromal cells, immune cells, and various secreted factors and signaling molecules [358]. These components interact in complex ways, influencing the aggressiveness of PC and its response to treatment. PDAC is characterized by an atypical and highly heterogeneous TME, which significantly contributes to its poor prognosis. Different protein metabolism pathways can affect the composition of the TME in PC. The metabolic environment within the TME poses a barrier to tumor-infiltrating immune cells, impacting the efficacy of clinical immunotherapy. Metabolic communication between cancer cells and immune cells significantly influences the nature and magnitude of the immune response, highlighting the crucial role of substance metabolism in immune surveillance and evasion [359]. Aberrant expression of CD73, a critical enzyme in ATP metabolism, upregulates CCL5 levels through tumor cell-autocrine adenosine–Adora2a signaling, which activates the p38–STAT1 axis. This process leads to the recruitment of regulatory T cells (Tregs) to pancreatic tumors, creating an immunosuppressive microenvironment [360]. Additionally, arginine metabolism facilitates the internalization and degradation of MRC1-dependent collagen, alters macrophage phenotypes, and promotes intratumoral fibrosis in PC [361]. L-arginine also increases antitumor activity by regulating T-cell metabolism. Furthermore, researchers have identified proteins that undergo modifications in response to elevated L-arginine levels [362]. The metabolism of key proteins in the tumor microenvironment significantly influences the progression of PC. The glycolytic inhibitor 2-deoxy-D-glucose (2DG) induces energy starvation, which impacts the viability of various cancer cell lines [363]. Kousuke et al. performed a proteomic analysis of a PC cell line following 2DG treatment and confirmed that 2DG reduces N-glycosylation of proteins, which is associated with increased phosphorylation of GFAT1. This process ultimately inhibits cell growth through ER stress in PC cells [364]. Additionally, Tian et al. systematically analyzed intercellular signaling networks within the tumor microenvironment of PDAC. Their study revealed dysregulated communication mechanisms between cancer cells and stromal cells. Furthermore, prior study indicated that the regulation of key signaling axes (such as PDGF), and membrane protein shedding (such as AXL shedding), significantly contribute to the progression of PDAC [365]. This finding adds a new layer to our understanding of intercellular signaling regulation within the PC microenvironment. Notably, AXL shedding levels may be correlated with lymph node metastasis, suggesting its potential as an effective biomarker for PC diagnosis. Overall, these findings not only delineate microenvironmental communication networks but also provide translational targets for PDAC diagnosis and therapeutic development [365]. The most common way to study the tumor immune microenvironment in PC involves creating sections of fresh cancerous pancreatic tissue via precision instruments. Important immune cells that constitute the tumor microenvironment, such as macrophages (cell surface protein markers CD68C, CD163C, and HLA-DRC) and T cells (cell surface protein markers CD3C, CD8C, and FOXP3C), as well as stromal myofibroblasts (aSMAC), were present throughout the culture period [364]. The proteomic results revealed that the expression of antigenic proteins on the surface of immune cells in PC patients was greater than that in normal controls. Immunophenotyping in the tumor microenvironment is a key clinical indicator for monitoring disease progression [366]. Macrophages are among the most abundant cell types in the PC tissue stroma and play a key role in driving the progression of PC [367]. Liu et al. revealed the critical role of macrophages in the PC microenvironment in the initiation and progression of PC-associated cachexia [368]. Specifically, macrophages activate TNF-like weak inducer of apoptosis (TWEAK) through the CCL5/TRAF6/NF-κB pathway, leading to muscle atrophy. Moreover, tumor cells recruit and reprogram macrophages via the CCL2/CCR2 axis, creating a reciprocal interaction between PC cells and macrophages [368]. Disruption of the interaction between tumor cells and macrophages reduces muscle wasting, demonstrating that macrophages may serve as both predictive biomarkers for cachexia development and promising therapeutic targets for treating PC-associated cachexia. Researchers have further proposed the concept of a subtumor microenvironment. The subtumor microenvironment mostly expresses proteins involved in the cellular stress response, including growth factors with CAF-activating and immunomodulatory functions, cytokines, and cellular immunity [369]. Thus, the differential expression of proteins in the subtumor microenvironment of human PC patients suggests that these patients differ significantly from normal individuals in terms of matrix effector functions. Clinically, the diagnosis and prediction of PC can be based on variations in the expression of specific cellular proteins in the tumor microenvironment [370]. Recent proteomics analyses have indicated that integrin beta 3 (ITGB3) and TGFB1/2 could be key players in increasing fibroblast movement and inhibiting apoptosis, followed by the activation of mothers against decapentaplegic homolog 3 (SMAD3), signal transducer and activator of transcription 3 (STAT3), and Bcl2-associated athanogene 3 (BAG3), which are vital for chemotaxis, cell adhesion, and actin cytoskeleton signaling [371].

#### Inflammation in PC

Inflammation plays a pivotal role in PDAC pathogenesis, with CP and familial pancreatitis patients exhibiting a 50-fold elevated risk of PDAC compared with the general population [372]. Emerging research highlights the profound impact of innate inflammatory responses on PDAC progression, with the inflammatory microenvironment facilitating tumor growth by activating oncogenic signaling pathways that promote cancer cell survival and proliferation [373]. Acinar-to-ductal-metaplasia (ADM), a fundamental repair mechanism upon pancreatic injury, is widely recognized as a critical precancerous lesion. McAllister et al. demonstrated that IL-17-producing immune cells within the tumor microenvironment accelerate PanIN progression [374]. Notably, while neutralization of IL-17 did not affect ADM initiation, it potently inhibited the malignant transformation of ADM into early neoplasia. Similarly, IL-6 knockout in KPC mice delayed tumor progression without impacting ADM formation [375]. These findings establish a causal link between innate inflammation and PDAC progression but leave unresolved the direct role of cytokine signaling in ADM initiation—a critical gap in understanding inflammation-driven carcinogenesis. A previous study identified IL-22 as an essential mediator of ADM formation during pancreatitis, providing mechanistic insight into epithelium-immune crosstalk in pancreatic oncogenesis [376]. Del Poggetto et al. reported that recurrent inflammatory insults induce long-term “epithelial memory” in acinar cells, particularly in the context of *KRAS* mutation [377]. Transient inflammation triggers adaptive epigenetic reprogramming that initially confers cytoprotection but, when combined with oncogenic *KRAS* signaling, paradoxically promotes malignant transformation. This epigenetic memory modulates cellular plasticity: while reducing metaplastic responses to secondary injury, it enhances tumorigenic potential by amplifying *KRAS*-driven oncogenic signaling. Subsequent studies confirmed that this epigenetic priming, in the presence of oncogenic *Kras*, drives enhanced tumorigenesis despite attenuated metaplastic responses to repeated injury [378]. Notably, in *Kras*^G12D^ murine models, inflammatory stimuli accelerate early pancreatic neoplastic transformation through the activation of mTORC1 and mTORC2 complexes, which promote Arp2/3 complex-mediated actin reorganization to synergize with *Kras*^G12D^-driven ADM progression in vivo and in vitro [379]. The interleukin (IL) family of cytokines comprises 38 members, including IL-1, IL-2, IL-4, IL-6, IL-12, and IL-22, with diverse roles in mediating cellular immunity, resistance to microbial infection, and antitumor activity. In PDAC, IL-1 is a key mediator of inflammatory signaling. It contributes to PC progression via multiple pathways. (1) IL-1 antagonizes the JAK/STAT signaling pathway, resulting in CAF heterogeneity in PC. (2) IL-1 receptor antagonists suppress PC growth by blocking NF-κB activation [380]. (3) The IL-1/IL-1 receptor axis and the ASC inflammasome in tumor cells regulate thymic stromal lymphopoietin (TSLP) secretion by CAFs [381]. (4) IL-1β promotes desmoplasia and immunosuppression in PC [382, 383]. In addition to IL-1, IL-6 drives tumor progression, and combined blockade of IL-6 and PD-L1 synergistically inhibits tumor growth in murine models [384]. IL-4 and IL-13, which are frequently detected in the PDAC microenvironment, play context-dependent roles [385]. Collectively, the interleukin family orchestrates multifaceted inflammatory networks in PDAC, with roles ranging from immune modulation to stromal reprogramming.

### PTMs in PC

PTMs, which involve more than 400 chemical alterations at protein termini or amino acid residues (e.g., acetylation, phosphorylation, glycosylation, ubiquitination, and methylation), critically regulate protein function, localization, and turnover. Dysregulation of these PTMs contributes to oncogenesis and immune evasion, positioning PTMs as pivotal targets for cancer diagnostics and therapy [386]. In PDAC, PTMs drive stage-specific carcinogenesis processes, with global proteomic studies increasingly revealing their therapeutic potential in precision oncology. Histones, chromosomal proteins rich in arginine (Arg) and lysine (Lys), form the nucleosomal core and are dynamically modified by lysine acetyltransferases (KATs) and deacetylases (KDACs) [387]. Histone acetylation alters chromatin accessibility and gene expression, with dysregulation implicated in PDAC initiation and progression [388]. In Kras-mutant acinar cells, acetylation imbalance is an early molecular event in pancreatic carcinogenesis [389]. In PDAC, elevated histone acetylation is correlated with high stromal content and poor prognosis [390]. Coculture with pancreatic stellate cells induces histone acetylation reprogramming, while metastatic PDAC lesions display global histone hyperacetylation linked to glucose metabolic reprogramming [391, 392]. Notably, the AKT pathway governs histone acetylation: AKT inhibition reduces H3/H4 acetylation marks and suppresses ADM in Kras-mutant cells, with Kras driving acetylation via AKT signaling [389]. Targeting histone acetylation with BET inhibitors or HDAC inhibitors (e.g., romidepsin) suppresses tumor growth by modulating FOXM1 expression and reactive oxygen species (ROS) accumulation [393]. Proteomic studies have also revealed acetylation in thousands of nonhistone proteins [394]. For example, STAT3 acetylation remodels the immune microenvironment via circPTPN22-dependent SIRT1 suppression, while the acetylation of lactate dehydrogenase A (LDH-A) enhances its stability and metabolic reprogramming [395, 396].

Phosphorylation is a reversible PTM that regulates cellular signal networks through kinases and phosphatases. A murine proteomic/phosphoproteomic atlas identified 50,000 phosphorylation sites, linking KRAS signaling to druggable kinases (e.g., AKT, CDK7, SRC) [397]. These findings establish phosphorylation networks as central to *KRAS* oncogenicity and potential hubs for combination therapy. Glycosylation, particularly *N/O*-glycosylation, remains underexplored in PDAC but is linked to tumor progression. *N*-glycosylation, catalyzed by oligosaccharyltransferases in the ER, is altered in inflammatory and metastatic contexts [398]. Li et al. quantified 703 glycoproteins and identified 426 *N*-glycosites in PDAC-associated inflammatory processes, while integrative glycoproteomics revealed synergistic effects of aspirin and gemcitabine [399]. ST6 β-galactoside α2,6 sialyltransferase 1 (ST6GAL1), which mediates the α2,6-sialylation of *N*-glycans, is upregulated in early-stage PDAC and accelerates ADM and tumorigenesis in *Kras*^G12D^ mice [400, 401]. Mechanistically, ST6GAL1 enhances the activation of EGFR, a driver of ADM, through sialylation-dependent signaling. *O*-glycosylation, which is catalyzed by Golgi-resident glycosyltransferases, promotes PDAC invasiveness [402]. Truncated *O*-glycans in T3M4 cells increase proliferation and EMT, whereas Wnt signaling regulates *O*-glycosylation of α-dystroglycan (α-DG) in pancreatic progenitor cells [403]. These findings highlight glycosylation as a modulator of early neoplastic transformation. Additionally, emerging PTMs related to PC pathology have been reported. Lipidation is a common PTM. Lipid metabolism reprogramming, including mTORC1-LIPIN1-YME1L axis activation, supports PDAC growth, with lipid transfer proteins identified as therapeutic targets [404]. Methylation can affect different signaling pathways to promote the progression of carcinogenesis. LKB1 loss links serine metabolism to DNA methylation, whereas METTL13-driven eEF1AK55 methylation promotes tumorigenesis [405]. Some studies have shown that FBW7 ubiquitination of oncoproteins and STAT3 S-nitrosylation regulate PDAC progression, with NOS inhibitors modulating these pathways [406]. Collectively, investigations into the proteomic landscape of PDAC across diverse PTMs have deepened our understanding of its oncobiology, unmasking functionally critical proteins as actionable therapeutic targets and providing a robust framework for validating additional vulnerabilities.

### Drug resistance mechanisms of PC

PC is highly resistant to current chemotherapy treatments because of its desmoplastic characteristics and limited blood supply, which hinder effective drug delivery to the tumor. Moreover, PDAC is one of the most stroma-rich cancers, with stromal tissue accounting for 50–80% of the tumor volume [407]. This stroma not only supports tumor growth and metastasis but also serves as a physical barrier to drug delivery, contributing to resistance against conventional therapies. Cells that survive cancer treatment with anticancer drugs exhibit specific protein expression and molecular mechanisms, which are associated with low survival rates in patients [408]. Proteomic methods can identify the characteristics of drug-resistant cancer cells and identify targets to overcome resistance during anticancer treatment. A study performed global quantitative proteomic profiling to compare the proteins expressed in gemcitabine-sensitive parental cells with those expressed in gemcitabine-resistant (GemR) cells. GemR cells adapt to multiple pathways in response to gemcitabine-induced stress. Specifically, the gemcitabine metabolic pathway has emerged as a predominant contributor to chemoresistance, with altered expression of critical proteins directly influencing gemcitabine responsiveness. These findings suggest that therapeutic strategies targeting the gemcitabine metabolic axis through novel drug combinations could enhance gemcitabine efficacy in resistant phenotypes [409]. Kim et al. analyzed proteomes from the PANC-1, BxPC-3, and HPDE cell lines to identify protein expression patterns associated with gemcitabine resistance [410]. These findings indicate that proteins associated with EMT and glutathione metabolism play crucial roles in this resistance. Baron et al. developed gemcitabine-resistant (KLM1-R) and growth factor-limited survival (KLM1-S) cell lines to investigate both direct and indirect mechanisms of chemoresistance [411]. Both cell lines presented similar levels of resistance, which was influenced by proteins including transitional endoplasmic reticulum ATPase (VCP), LIM/homeobox protein (LHX1), prelamin A/C (LMNA), heat shock protein 60 (HSP60), and alpha-enolase (ENO1). Large et al. reported that MAP2 and ankyrin-3 are significantly upregulated and hyperphosphorylated in gemcitabine-resistant cell lines, suggesting their potential role in drug resistance [408]. Zhou et al. reported a threefold increase in inflammatory cancer-associated fibroblasts in chemoresistant patients with PDAC who were treated with gemcitabine and nab-paclitaxel [266]. Metallothionein proteins are associated with resistance to various chemotherapeutic agents and may indicate a mechanism of chemoresistance [412]. Pancreatic stellate cells (PSCs) are essential components of the tumor stroma that interact dynamically with PCCs to create a signaling network centered around extracellular matrix (ECM) proteins, which collectively promote the progression of PDAC. For example, a proteomic analysis of conditioned media from 10 primary human PSC cultures demonstrated that PSC-secreted fibronectin induces GemR resistance in PCCs by activating the ERK1/2 phosphorylation pathway [413]. In a parallel study, comparative proteomics of exosomes isolated from three PC cell lines with varying sensitivities to gemcitabine resistance was performed. Importantly, the expression level of ephrin type-A receptor 2 (EphA2) may serve as a minimally invasive predictive biomarker for therapeutic response in PDAC patients [152]. In addition to gemcitabine, oxaliplatin is a commonly used chemotherapy drug for PC treatment. Quantitative proteomic analysis of both oxaliplatin-resistant and oxaliplatin-sensitive PANC-1 cells revealed that the AKT and β-catenin signaling pathways are activated in resistant cells. Moreover, siRNA-mediated inhibition of MARCKS in animal models demonstrated that targeting the Axl receptor tyrosine kinase can promote apoptosis and inhibit 14-3-3ζ/Axl signaling. Although the combination of gemcitabine and nab-paclitaxel (n-PTX) modestly improved survival, sustained c-MYC induction was found to drive n-PTX resistance in primary PDAC cells [414]. Overall, these studies elucidate the molecular mechanisms underlying drug resistance and suggest synergistic therapeutic strategies to overcome treatment challenges in PC.

## Future perspectives

Recent advancements in single-cell and high-dimensional analytical technologies have profoundly improved our understanding of PDAC, revealing that its intratumoral heterogeneity is far more complex than previously appreciated. This complexity, which is evident across malignant cell subpopulations, stromal cells, and immune cell compartments, underscores the need for spatially and temporally resolved proteomic approaches to inform clinical diagnosis and therapeutic strategies. Traditional proteomics still needs innovations in microfluidics and chip-based technologies to achieve high-throughput resolution. Single-cell proteomics by mass spectrometry (SCoPE-MS) has emerged as a powerful method for the quantitative analysis of multiplexed single-cell proteomes. This approach employs isotope labeling and carrier proteomics to enable detailed proteomic analysis at the individual cell level [415]. Recent advancements in trapped ion mobility time-of-flight mass spectrometry have significantly improved our ability to quantify approximately 953 proteins per cell [416]. While its sensitivity is still lower than that of genomic-scale analyses (~ 20,000 genes), this approach is effective in evaluating inhibitors that target specific KRAS mutations in PDAC. Deep learning frameworks have been proposed to address batch effects and data noise, although they still require real-world validation. Additionally, multimodal integration has successfully mapped the spatial proteomic heterogeneity of 14 distinct cell types within individual tumors at single-cell resolution [417]. A hallmark of PDAC is its intricate stromal microenvironment, which necessitates high-resolution spatial proteomics. To overcome technical hurdles, such as obtaining nanogram-scale protein yields from laser-captured microdissected (LCM) tissues, optimized workflows such as the SISPROT platform have been developed to increase sensitivity [418]. Additionally, innovative approaches now combine immunohistochemistry-guided processing for formalin-fixed samples with specialized glycoproteomic techniques to map spatial protein modifications [419]. Furthermore, AI-powered image analysis systems facilitate automated cell-type recognition and large-scale data processing [420]. Secreted and membrane-bound proteins (S-PM proteins) play essential roles in regulating intercellular signaling and serve as crucial biomarkers and therapeutic targets. In their study of PDAC, Tian et al. conducted a five-dimensional analysis of S‒PM proteins using a limited number of tumor samples. These multidimensional datasets offer critical insights for systematically mapping intercellular signaling networks and provide a valuable theoretical basis for identifying molecular targets in the diagnosis and treatment of PC [365]. While single-cell RNA sequencing and spatial transcriptomics have shed light on transcriptional heterogeneity in the PDAC stroma, parallel proteomic studies at single-cell resolution are critical for directly analyzing functional protein networks and identifying molecular signatures that differentiate malignant and stromal lineages within the tumor microenvironment [421]. In the future, proteomics will be a rapidly advancing field that has the potential to transform our understanding of PC biology and pave the way for more effective diagnostic and treatment strategies. By using cutting-edge techniques such as mass spectrometry and bioinformatics, researchers can identify and measure protein expression patterns linked to the progression of PC, treatment response, and patient prognosis.

## Conclusion

Proteomics, a multidisciplinary strategy that focuses on protein expression, modification, and interaction networks in living organisms, has emerged as a cornerstone of cancer research, particularly in the realm of liquid biopsy. By analyzing proteins in biofluids or tissues, proteomic technologies enable the discovery of cancer-associated biomarkers that inform tumor typing, progression staging, and treatment response prediction. In PC, MS-based proteomics was used to establish causal links between risk factors (e.g., chronic inflammation, KRAS mutation) and oncogenesis, identify diagnostic/prognostic biomarkers (e.g., IL-17, ST6GAL1) and therapeutic targets involved in immune-stromal crosstalk and PTM networks. The integration of proteomics with other methodologies holds immense promise for precision oncology. For example, single-cell proteomics and spatial proteomics have begun to unravel the intratumoral heterogeneity of PC, dissecting malignant-stromal-immune cell interactions with unprecedented resolution. Such insights are critical for developing personalized treatment strategies, such as PTM-targeted therapies or combinatorial regimens that address both oncogenic signaling and microenvironmental complexity. However, several challenges must be addressed to translate proteomic discoveries into clinical practice. Standardization of sample collection, preparation, and MS-based detection protocols remains essential for ensuring biomarker reproducibility across cohorts. The computational analysis of large-scale proteomic and multiomics datasets also demands more sophisticated AI-driven algorithms to mitigate batch effects, enhance data integration, and identify robust molecular signatures. Moreover, the intrinsic heterogeneity of PC necessitates longitudinal studies with large, diverse patient cohorts to validate biomarker utility and treatment efficacy. In the future, advancements in high-dimensional proteomics, including enhanced single-cell sensitivity, spatial mapping of PTMs, and AI-powered data analytics, will deepen our mechanistic understanding of PC and facilitate the development of early detection tools and targeted therapies. By addressing technical bottlenecks and embracing interdisciplinary collaboration, proteomics is poised to transform PC management, offering hope for improved outcomes in this notoriously refractory disease.

## Electronic supplementary material

Below is the link to the electronic supplementary material.

Supplementary Material 1

## Data Availability

No datasets were generated or analysed during the current study.
