# Supplementary material for: Proteomics in pancreatic cancer
Source: Biomark Res. 2025 Jul 6;13:93. doi: 10.1186/s40364-025-00805-y (PMC12232871; doi:10.1186/s40364-025-00805-y)
Supplement: Supplementary file 1 — Supplementary Material 1 [file 40364_2025_805_MOESM1_ESM.doc]

**Supplementary Information**

**Proteomics in pancreatic cancer**

Fei Cai^1 #^, Yufan Gu^1 #^, Yingying Ling^1^, Guanghua Yi^1^, Shengze Zang^1^, Tao Su^1^, Yueqiu Liu^1^, Ang Li^1^, Denian Wang^2^, Wanjun Zhao^3^, Xinfang Xie^4^, Guisen Li^5^, Lunzhi Dai^6^, Meng Gong^1^, Hao Yang^7^, Yang Zhao^8 *^, Yong Zhang^1 *^

^1^Department of Pancreatic Surgery and Institutes for Systems Genetics, West China Hospital, Sichuan University, Chengdu 610041, China;

^2^Precision Medicine Center, Precision Medicine Key Laboratory of Sichuan Province, State Key Laboratory of Respiratory Health and Multimorbidity, West China Hospital, Sichuan University, Chengdu 610041, China;

^3^Division of Thyroid Surgery, Department of General Surgery, West China Hospital, Sichuan University, Chengdu 610041, China;

^4^Department of Nephrology, The First Affiliated Hospital of Xi'an Jiaotong University, Xi’an 710061, China;

^5^Renal Department and Institute of Nephrology, Sichuan Provincial People's Hospital, University of Electronic Science and Technology of China, Sichuan Clinical Research Center for Kidney Diseases, Chengdu 611731, China;

^6^National Clinical Research Center for Geriatrics, State Key Laboratory of Biotherapy, West China Hospital, Sichuan University, Chengdu 610041, China;

^7^Transplant Center and NHC Key Lab of Transplant Engineering and Immunology, West China Hospital, Sichuan University, Chengdu 610041, China;

^8^Technology Innovation Center of Mass Spectrometry for State Market Regulation, Center for Advanced Measurement Science, National Institute of Metrology, Beijing 100029, China.

^#^These authors contributed equally to this work.

*Correspondence: Yong Zhang (nankai1989@foxmail.com); Yang Zhao (zhaoy@nim.ac.cn)

**Table of Contents**

**Supplementary Table 1.** Some clinical trials of palliative chemotherapy, immunotherapies and targeted therapy in patients with pancreatic cancer.

**Supplementary Table 2.** Diagnostic protein biomarkers in pancreatic cancer identified with proteomic approaches.

**Supplementary Table 3.** Protein biomarkers of sEV used for diagnosis/prognosis in PDAC with proteomics approaches.

**Supplementary Table 4.** Diagnostic biomarker panel mentioned in this paper that combine with CA19-9.

**Supplementary Table 5.** Some prognosis protein biomarkers in pancreatic cancer identified with proteomics approaches.

| **Supplementary Table 1.** Some clinical trials of palliative chemotherapy, immunotherapies and targeted therapy in patients with pancreatic cancer | | | | | | | | |  |
| --- | --- | --- | --- | --- | --- | --- | --- | --- | --- |
| **Treatment method/Target** | **Drugs** | **NCT** | **Phase** | **No. of patients** | **mOS** | **mPFS** | **ORR** | **Ref.** | |
| **Chemotherapy** | | | | | | | | |  |
| *First-line treatment* | | | | | | | | |  |
| Chemotherapy | FOLFIRINOX vs. GEM | NCT00112658 | Phase III | 342 | 11.1 vs 6.8 months (HR 0.57, *p* < 0.001) | 6.4 vs. 3.3 months | 31.6% vs. 9.4%, *p* < 0.001 | [^1^](#_ENREF_1) | |
|  | GEM + NabP vs. GEM | NCT00844649 | Phase III | 861 | 8.5 vs. 6.7 months (HR 0.72, *p* < 0.001) | 5.5 vs. 3.7 months (HR 0.69, *p* < 0.001) | 23% vs. 7%, *p* < 0.001 | [^2^](#_ENREF_2) | |
|  | NALIRIFOX vs. Gem+NabP | NCT04083235 | Phase III | 770 | 11.1 vs 9.2 months (HR 0.83, *p* = 0.036) | 7.4 vs. 5.6 months (HR 0.7, *p* < 0.0001） | NA | [^3^](#_ENREF_3) | |
|  | Elraglusib + GEM/NabP vs. GEM/NabP | NCT03678883 | Phase II | 233 | 9.3 vs. 7.2 months (HR 0.63, *p*= 0.016) | 5.6 vs. 4.9 months (HR 0.91, *p*= NS) | 27.7% vs. 20.5% | [^4^](#_ENREF_4) | |
| *Second-line treatment* | | | | | | | | |  |
| Chemotherapy | Liposomal irinotecan HR070803 +5-FU/LV vs. Placebo+5-FU/LV | NCT05074589 | Phase III | 298 | 7.4 vs. 5.0 months (HR 0.63, *p* = 0.0019) | 4.2 vs. 1.5 months (HR 0.36, *p* < 0.001) | 12.8% vs. 0.7% | [^5^](#_ENREF_5) | |
|  | Paclitaxel + GEM vs. GEM | NCT03943667 | Phase III | 211 | 6.4 vs. 5.9 months (HR 0.87, *p* = 0.4095) | 3.1 vs. 2.0 months (HR 0.64, *p* = 0.0067) | 17.1% vs. 4.2%, *p* = 0.008 | [^6^](#_ENREF_6) | |
| Adjuvant chemotherapy treatment | mFOLFIRINOX vs. GEM | NCT01526135 | Phase III | 493 | 53.5 s vs. 35.5 months (HR 0.68, *p* = 0.001) | 21.4 vs 12.8 months (HR 0.58, *p* < 0.001) | NA | [^7^](#_ENREF_7) | |
| **Targeted Therapies** | | | | | | | | |  |
| *First-line treatment* | | | | | | | | |  |
| Tyrosine kinase inhibitors | Ibrutinib + GEM/NabP vs. Placebo + GEM/NabP | NCT02436668 | Phase III | 424 | 9.7 vs. 10.8 months; *p* = 0.3225 | 5.3 vs. 6.0 months; *p* < 0.0001 | 29% vs. 42%, *p*= 0.0058 | [^8^](#_ENREF_8) | |
| Extracellular matrix targeting | GEM/NabP + PEGPH20 vs. GEM/NabP + placebo | NCT02715804 | Phase III | 494 | 11.2 vs. 11.5 months (HR 1.00, *p* = 0.97 | 7.1 vs. 7.1 months (HR 0.97) | 47% vs. 36% (ORR ratio, 1.29) | [^9^](#_ENREF_9) | |
| *Second-line treatment* | | | | | | | | |  |
| BRCA1/2 Germline Mutation | Olaparib vs. Placebo | NCT02184195 | Phase III | 154 | 18.9 vs. 18.1 months (HR 0.91, *p* = 0.68) | 7.4 vs. 3.8 months (HR 0.53, *p* = 0.004) | NA | [^10^](#_ENREF_10) | |
| KRAS^G12C^ | Sotorasib (AMG 510) | NCT03600883 | Phase II | 38 | 6.9 months (95% CI, 5.0–9.1) | 4.0 months (95% CI, 2.8–5.6) | 21% | [^11^](#_ENREF_11) | |
| **Immunotherapies** | | | | | | | | |  |
| *First-line treatment* | | | | | | | | |  |
| Chemotherapy+Immunotherapies | Penpulimab+anlotinib and GEM/NabP | NCT05493995 | Phase II | 66 | 13.7 months (95% CI, 12.4-not reached) | 8.8 months (95% CI, 8.1–11.3) | 50% | [^12^](#_ENREF_12) | |
| PD-1 + Chemotherapy | Nivo + GEM/NabP vs. Soti+GEM/NabP vs. Nivo+Soti+GEM/NabP | NCT03214250 | Phase II | 105 | 5.2 vs. 5.1 vs. 4.7 months | 6.4 vs. 7.3 vs. 6.7 months | 50% vs. 33% vs. 31% | [^13^](#_ENREF_13) | |
| Mitochondrial tricarboxylic acid | Devimistat+mFOLFIRINOX vs. mFOLFIRINOX | NCT03504423 | Phase III | 528 | 11.10 vs. 11.73 months (HR 0.95, *p* = 0.655) | 7.8 vs. 8.0 months (HR 0.99, *p* = 0.94) | 39.1% vs. 34.4% | [^14^](#_ENREF_14) | |
| Cancer vaccine + Chemotherapy | HAPa+FOLFIRINOX +GEM/NabP vs. FOLFIRINOX+ GEM/NabP | NCT01836432 | Phase III | 303 | 14.3 vs. 14.9 months (HR 1.02, *p* = 0.98) | 12.4 vs. 13.4 months (HR 1.33, *p* = 0.59) | NA | [^15^](#_ENREF_15) | |
| *Second-line treatment* | | | | | | | | |  |
| CAR T cell therapy | Claudin18.2-redirected CAR T cells: CT041 | NCT03874897 | Phase I | 5 | 10.0 months | 3.3 mouths | 16.7% | [^16^](#_ENREF_16) | |
| **Abbreviations:** mOS, median overall survival; mPFS, medium progression-free survival; ORR, objective response rate; FOLFIRINOX= a combination of leucovorin, irinotecan, oxaliplatin and 5-fluorouracil; GEM, gemcitabine; HR, hazard ratio; NabP, nab-Paclitaxel; GEM/NabP, gemcitabine/nab-paclitaxel; 5-FU, 5-fluorouracil; LV, leucovorin; NALIRIFOX=a combination of liposomal irinotecan, oxaliplatin, leucovorin and 5-fluorouracil; ORR, objective response rate; median disease-free survival ; (m)FOLFIRINOX=a (modified) combination of 5-fluorouracil with leucovorin, irinotecan, and oxaliplatin; Nivo, nivolumab; Soti, Sotigalimab; NA, not available; mKRAS, mutant KRAS. | | | | | | | | |  |

| **Supplementary Table 2.** Diagnostic protein biomarkers in pancreatic cancer identified with proteomic approaches. | | | | | |
| --- | --- | --- | --- | --- | --- |
| **Biomarkers** | **Sample types** | **Proteomic quantification methods** | **Validation techniques** | **Significance** | **Ref.** |
| C4BPA | Serum | TMT labelling and LC-MS/MS | ELISA | C4BPA in serum can effectively distinguish PDAC from CP and other gastrointestinal cancers (*p* < 0.001) with an AUC of 0.86. | [^17^](#_ENREF_17) |
| NGAL | Urine | None | ELISA | Urinary NGAL (cutoff 27 ng/mL) accurately differentiated CP from PDAC (81% sensitivity, 80% specificity). | [^18^](#_ENREF_18) |
| ANXA1 and ANXA10 | Tissues | LFQ | IHC | ANXA1/ANXA10 effectively discriminated PDAC from CCC in liver metastases (AUC=0.907/0.829; *p* < 0.001). | [^19^](#_ENREF_19) |
| GPC1 | Exosomes isolated from CM and plasma | UPLC–MS | ELISA and Flow cytometry | GPC-1 in sEVs showed higher level in PDAC patients than healthy donors with *p*<0.0001. GPC1+ crExos are a prognostic marker superior to CA 19-9. | [^20^](#_ENREF_20) |
| Glypican-1 and CD63 | Blood | ACE Microarray Chips | None | Combined Glypican-1/CD63 exosomal model achieves 99% sensitivity, 82% specificity in PDAC detection (no pretreatment). | [^21^](#_ENREF_21) |
| Tu M2-PK | Plasma | ELISA | None | Tu M2-PK (cutoff 27 U/mL) exhibited modest diagnostic value for periampullary cancer (AUC=0.623) yet strongly predicted poor prognosis (levels >27 U/mL linked to metastasis and poor differentiation). | [^22^](#_ENREF_22) |
| Tu M2-PK | Plasma | ELISA | None | Serum Tu M2-PK demonstrated limited diagnostic utility in advanced PDAC (55% sensitivity, 52% specificity) but maintained independence from Lewis phenotype/cholestasis. | [^23^](#_ENREF_23) |
| CEACAM1 | Serum | IHC and ELISA | RT-PCR | CEACAM1 demonstrated significantly higher expression in PDAC than noncancerous pancreas (P<0.0001), with an AUC of 0.936 surpassing CA19-9 (AUC=0.948 combined). | [^24^](#_ENREF_24) |
| CA19-9, IGFBP2 and IGFBP3 | Plasma | RPPA and LC-MS/MS | MRM-MS | A multivariate model combining CA19-9, IGFBP2, and IGFBP3 demonstrated superior diagnostic accuracy for PDAC (AUC=0.90, 95%CI:0.837-0.962) versus CA19-9 alone. | [^25^](#_ENREF_25) |
| IGFBP2 and MSLN | Serum | ELISA | None | IGFBP2/MSLN complement CA19-9 diagnostic limitations, with IGFBP2 levels positively correlating with tumor progression (*p* < 0.0001). | [^26^](#_ENREF_26) |
| Osteopontin | Serum | ELISA | None | Compared to healthy controls, elevated OPN of had sensitivity of 80% and specificity of 97% for pancreatic cancer. | [^27^](#_ENREF_27) |
| MIC-1 | Serum | NR | Meta-analysis | MIC-1 showed comparable diagnostic performance to CA19-9 (80% sensitivity, 85% specificity; AUC=0.895, DOR=24.57). | [^28^](#_ENREF_28) |
| Dysbindin (DTNBP1) | Serum | RPLC and MALDI-TOF- MS | ELISA | Serum dysbindin (cutoff 699.16 pg/ml) showed superior diagnostic efficacy (AUC=0.85) versus CA19-9, particularly in CA19-9-negative PDAC. | [^29^](#_ENREF_29) |
| AGP1 | Tissues and serum | LC-MS/MS | PRM and IHC | Overexpression of AGP1 independently predicted poor prognosis in PDAC, with serum levels distinguishing resectable cases from controls (AUC=0.837; AUC=0.963 combined with CA19-9). | [^30^](#_ENREF_30) |
| CFB | Plasma | LC−MS/MS | ELISA | Plasma CFB (AUC=0.958) showed twice the expression in PC versus controls (*p* < 0.0001), achieving 90.1% sensitivity with 97.2% specificity when combined with CA19-9. | [^31^](#_ENREF_31) |
| LIF | CM, tissues and plasma | Dimethyl labeling and LFQ | ELISA and PRM-MS | Stroma-derived LIF correlated with tumor differentiation and prognosis, demonstrating superior therapeutic monitoring capability over CA19-9. | [^32^](#_ENREF_32) |
| IL-11 | Plasma | ELISA | None | Elevated baseline IL-11p levels in PC patients versus healthy controls (P<0.001) demonstrated diagnostic potential (AUC=0.901, 97.7% sensitivity). Higher IL-11p (≥43.2 pg/mL) correlated with prolonged survival, particularly at ≥50 pg/mL. | [^33^](#_ENREF_33) |
| MIC-1 | Serum | ELISA | RT-PCR | MIC-1 showed elevated expression in PDAC tissues/serum (65.8% sensitivity), detecting 63.1% CA19-9-negative early-stage cases, with serum levels dynamically reflecting treatment response and recurrence. | [^34^](#_ENREF_34) |
| ARG2 | Pancreatic juice | None | ELISA | AGR2 exhibited progressive elevation in pancreatic juice (AUC=0.729 for PanIN3-PDAC transition) and increased plasma levels in PDAC patients. | [^35^](#_ENREF_35) |
| sLR11 | Bile | ELISA and RT-PCR | IHC | Biliary sLR11 (cutoff 7.50 ng/ml, AUC=0.89) outperformed CEA/CA19-9, achieving 100% sensitivity when combined with serum markers. | [^36^](#_ENREF_36) |
| **Abbreviations:** LC-MS/MS, **l**iquid chromatography-tandem mass spectrometry; MS/MS, tandem mass spectrometry; ELISA, electrochemiluminescence immunoassay; LFQ, label free quantitation; IHC, Immunohistochemistry; AUC, area under curve; PDAC, pancreatic ductal adenocarcinomas; CP, chronic pancreatitis; CCC, cholangiocellular carcinoma; UPLC-MS, ultra-performance liquid chromatography-mass spectrometry; CTCs, circulating tumor cells; RT-PCR, reverse transcription polymerase chain reaction; HPLC-TOF MS, high performance liquid chromatography-time of flight mass spectrometry; PRM, parallel reaction monitoring; RPLC, reversed-phase liquid chromatography; MALDI-TOF-MS, matrix-assisted laser desorption/ionization time of flight mass spectrometry; NanoLC-MS/MS, Nanoscale liquid chromatography coupled to tandem mass spectrometry; MRM-MS, multiple reaction monitoring mass spectrometry; PRM-MS, parallel reaction monitoring mass spectrometry. | | | | | |

| **Supplementary Table 3.** Protein biomarkers of sEV used for diagnosis/prognosis in PDAC with proteomics approaches. | | | | | | | |
| --- | --- | --- | --- | --- | --- | --- | --- |
| **Biomarkers** | **Sample types** | **Exosome isolation** | **Proteomic quantification methods** | **Validation techniques** | **Significance** | **Ref.** |  |
| GPC-1 | Serum | Ultracentrifugation | UPLC-MS | ELISA | GPC-1 in sEVs showed higher level in PDAC patients than healthy donors with *p* <0.0001. GPC1+ crExos are a prognostic marker superior to CA 19-9. | [^37^](#_ENREF_37) |  |
| EGFR, EpCAM, MUC1, GPC1, WNT2 | Plasma | Ultracentrifugation | Nanoplasmonic sensors measurement | ELISA | The five-marker signature yielded a more accurate diagnosis of PDAC than CA19-9 and a single sEV biomarker with sensitivity of 86% (CI, 65-97%) and a specificity of 81% (CI, 58-95%) in prospective cohort | [^38^](#_ENREF_38) |  |
| CEACAM1/5, tenascin C | PDF | Ultracentrifugation | LC-MS/MS | IHC | The median expression level of CEACAM1/5 and tenascin C among patients with PDAC was significantly higher than that of the benign patient. | [^39^](#_ENREF_39) |  |
| GPRC5C, EPS8 | Serum | ultracentrifugation and blood protein affinity columns | LC-MS/MS | WB | EV-associated GPRC5C and EPS8 are suitable for early-stage PDAC patient discrimination with an AUC of 0.922. | [^40^](#_ENREF_40) |  |
| KIF5B, SFRP2 | Cell lines | magnetic EVtrap beads | LC-MS/MS | IHC | KIF5B and SFRP2 show promise as early biomarkers that are highly expressed in progressive stages of PC. | [^41^](#_ENREF_41) |  |
| ZIP4 | Serum | SBI ExoQuick-TC Kit | LC-MS/MS | WB and IHC | The level of ZIP4 in sEVs showed promising diagnostic efficacy between PDAC and control group with AUC of 0.893. | [^42^](#_ENREF_42) |  |
| MIF | Plasma | ultracentrifugation | LC-MS/MS | WB and IHC | MIF was highly expressed in sEVs from PDAC patients (PDAC patients without liver metastasis vs. healthy controls *p* < 0.01). | [^43^](#_ENREF_43) |  |
| EphA2 | Plasma | No separation is required | nPES platform | ELISA | EphA2 in sEVs could distinguish pancreatic cancer patients from pancreatitis patients and healthy subjects with AUC of 0.93–0.96. | [^44^](#_ENREF_44) |  |
| GPC1, CD63 | Plasma,  serum | ultracentrifugation | AC electrokinetic microarray chip | IF | modelincorporating both GPC1 and CD63PDAC patient could discriminate the PDAC patients from healthy controls with 99% sensitivity and 82% specificity | [^21^](#_ENREF_21) |  |
| EpCAM | Plasma | No separation is required | ELISA | None | The level of EpCAM in sEVs in the plasma of PDAC patients was elevated, and the level of EpCAM increase was associated with better prognosis during palliative chemotherapy treatment. | [^45^](#_ENREF_45) |  |
| EphA2 | Serum | ExoQuick Exosome Precipitation Solution | ELISA | None | Serum Exo-EphA2 levels in PC patients were significantly higher than that in benign pancreatic disease and healthy control patients. | [^46^](#_ENREF_46) |  |
| GPC1, EpCAM, CD44V6 | Plasma | ultracentrifugation | surface-enhanced Raman scattering assay | None | The PDAC EV signature of the three protein biomarkers had high accuracy for PDAC diagnosis with AUC of 1.0 (95% CI: 84.6–100%) and showed strong correlation with cancer stages | [^47^](#_ENREF_47) |  |
| c-Met | Serum | Invitrogen Total Exosome Isolation Reagent | Flow cytometry | None | Diagnostic test based on c-Met in sEVs resulted in a sensitivity of 70%, a specificity of 85% | [^48^](#_ENREF_48) |  |
| CKAP4 | Serum | ultracentrifugation | ELISA | IHC/WB | The CKAP4 levels in sEVs were higher in patients with PDAC than healthy control individuals | [^49^](#_ENREF_49) |  |
| ANXA6 | Serum | Kit PS ultracentrifugation | rbitrap-MS and LTQ-MS/MS | WB/IHC/IF | ANXA6 level in sEVs could be used to diagnose PDAC patients with AUC of 0.979 and improved sensitivity and specificity | [^50^](#_ENREF_50) |  |
| ADAM8 | Serum | ultracentrifugation | FACS Analyses | WB | ADAM8 in EVs from PDAC patients or precursor lesions had significantly higher expression when compared to healthy individuals with *p*<0.0001or *p*=0.0139, respectively. And it gradually increased with increasing tumor staging. | [^51^](#_ENREF_51) |  |
| CD41, CD61,  CD63 | Serum | MagCaptureTM Exosome Isolation Kit PS | ELISA | None | The levels of CD41, CD61 and CD63 in sEVs increased in PDAC patients then healthy controls with AUC of 0.678, 0.652 and 0.846, respectively | [^52^](#_ENREF_52) |  |
| CD44v6,  C1QBP | Serum | Total Exosome Isolation kit ultracentrifugation | WB | IHC/IF | High expression of CD44v6 and C1QBP in sEvs predicts liver metastasis and poor survival in patients with PDAC | [^53^](#_ENREF_53) |  |
| LRG-1, GPC-1 | Plasma and serum | ultracentrifugation magnetic beads | LFQ | WB | Combination of LRG-1 and GPC-1 positive sEVs could improve the diagnostic accuracy of PDAC with AUC of 0.95, even for the early stage PDAC. | [^54^](#_ENREF_54) |  |
| Integrin α6 | Plasma | ultracentrifugation | MALDI-TOF-MS | 2D gel electrophoresis, WB and q-PCR | The expression of Integrin α6 in sEVs from blood of PDAC patients significantly decreased after surgery and increased before that of CA19-9 and other markers several months before clinical recurrence | [^55^](#_ENREF_55) |  |
| Multiple differentially expressed proteins | Plasma | miRCURY® Exosome Serum/Plasma Kit | NanoLC-MS/MS | None | Various differentially expressed plasmatic exosome-derived proteins among healthy controls, IPMN, and PDAC patients, which may represent alternative biomarkers for diagnosis and prognostic assessment. | [^56^](#_ENREF_56) |  |
| Mucin-4,  Mucin-5AC,  Mucin-6,  Mucin-16,  etc. | PDF, blood, and cell lines | Ultracentrifugation magnetic beads | UPLC | ELISA | The unique proteins detected in sEVs derived from pancreatic juice may serve as potential biomarkers for PDAC. | [^57^](#_ENREF_57) |  |
| Combination of 35  proteins | PDF | ultracentrifugation | LC-MS/MS (LFQ) | IHC | Exosome proteins in PDF were potential biomarkers of patients with different pancreatic diagnoses | [^39^](#_ENREF_39) |  |
| **Abbreviations:** UPLC-MS, ultra performance liquid chromatography mass spectrometry; ELISA, electrochemiluminescence immunoassay; sEVs, small extracellular vesicles; PDF, pancreatic duct fluid; PDAC, pancreatic ductal adenocarcinomas; LC-MS/MS, **l**iquid chromatography-tandem mass spectrometry; IHC,immunohistochemistry; WB, western blot; AUC, area under curve; IF, immunofluorescence; PC, pancreatic cancer; LFQ, label-free quantitative proteomics; MALDI-TOF-MS, matrix-assisted laser desorption/ ionization time of flight mass spectrometry; q-PCR, quantitative real-time PCR; NanoLC-MS/MS, nanoscale liquid chromatography coupled to tandem mass spectrometry; UPLC, ultra performance liquid chromatography; IPMN, intraductal papillary mucinous neoplasia. | | | | | | | |

| **Supplementary Table 4.** Diagnostic biomarker panel mentioned in this paper that combine with CA19-9 | | | | | |
| --- | --- | --- | --- | --- | --- |
| **Biomarker panels** | **Sample types** | **Proteomic quantification methods** | **Validation techniques** | **Significance** | **Ref.** |
| CA19-9, TIMP-1, and LRG1 | Serum | LFQ | ELISA | The model achieved AUCs of 0.949 (validation set) and 0.887 (test set) for early-stage PDAC vs healthy controls (sensitivity 0.849/0.667 at 95% specificity), significantly outperforming CA19-9 alone (p < 0.001 and p=0.008, respectively). | [^58^](#_ENREF_58) |
| CA19-9, IGFBP2 and IGFBP3 | Plasma | RPPA and LC-MS/MS | MRM-MS | This model discriminated well between controls and patients in the early-stage set (AUC, 0.900; 95% CI, 0.837–0.962). | [^25^](#_ENREF_25) |
| CFB and CA 19-9 | Plasma and cell lines | LC−MS/MS | ELISA, IP and qRT-PCR | The combined use of complement factor B (CFB) and CA19-9 significantly improves diagnostic performance for pancreatic cancer, achieving 90.1% sensitivity and 97.2% specificity. | [^31^](#_ENREF_31) |
| CA 19-9, APOA1, APOE, APOL1 and ITIH3 | Serum | iTRAQ labelling and LC-MS/MS | SID-MRM-MS | The biomarker panel combining APOE, ITIH3, APOA1, APOL1, and CA19-9 demonstrates significantly higher sensitivity (95%) and specificity (94.1%) for pancreatic cancer diagnosis compared to CA19-9 alone. | [^59^](#_ENREF_59) |
| CA19-9, APOA4, TIMP1 | Serum | SID-MRM-MS | IHC | This panel including CA 19-9, APOA4 and TIMP1 showed better performance for distinguishing early pancreatic cancer from pancreatitis (AUC = 0.934, 86% sensitivity at fixed 90% specificity) than CA 19-9 alone (71% sensitivity). | [^60^](#_ENREF_60) |
| CA19-9, LRG1 and TTR | Plasma | MRM-MS | ELISA and Immunoassay | The panel including LRG1, TTR, and CA19-9 had a sensitivity of 82.5% and a specificity of 92.1%, and the diagnostic performance AUC of 0.931 outperform CA 19-9 in differentiated PDAC from normal, benign diseases and other cancers. | [^61^](#_ENREF_61) |
| CA19-9, LRG1 and TTR | Plasma | ELISA | None | The triple-marker automated ELISA panel provided reliable prediction results with a positive predictive value of 92.05%, negative predictive value of 90.69%, specificity of 90.69%, and sensitivity of 92.05%, which all simultaneously exceed 90% cutoff value. | [^62^](#_ENREF_62) |
| CA19-9, LRG1 and TTR | Plasma | ELISA | None | The multi-marker panel demonstrated robust diagnostic accuracy in differentiating PDAC from normal/benign pancreatic conditions and other cancers, achieving positive and negative predictive value, sensitivity, and specificity were 94.12, 90.40, 93.81, and 90.86, respectively. | [^63^](#_ENREF_63) |
| CA19-9, LRG1 | Plasma | ELISA | None | CA19-9 alone shows AUC of 0.88 (95% CI: 0.83–0.94). The addition of LRG1 significantly increases diagnostic performance, elevating the AUC to 0.92 (95% CI: 0.88–0.96; p = 0.02). | [^64^](#_ENREF_64) |
| CA19-9, TIMP1 | Plasma | ELISA | None | CA19-9 alone achieves an AUC of 0.88 (95% CI: 0.83–0.94). With the addition of tissue inhibitor of TIMP1, the AUC increases to 0.92 (95% CI: 0.88–0.96; p = 0.06). | [^64^](#_ENREF_64) |
| THBS2 and CA 19-9 | Plasma | LC-MS/MS | ELISA | The combination of THBS2 and CA19-9 enhances the ability to differentiate PDAC from pancreatitis, achieving 87% sensitivity and 98% specificity. | [^65^](#_ENREF_65) |
| THBS1 and CA 19-9 | Serum | MRM-MS | MRM-MS | Circulating THBS1 levels decrease significantly up to 24 months prior to PDAC diagnosis and enhance the diagnostic performance of CA19-9. The combination of THBS1 and CA19-9 achieves an AUC of 0.86, outperforming either biomarker alone (AUC = 0.69 for THBS1, AUC = 0.77 for CA19-9; p < 0.01). | [^66^](#_ENREF_66) |
| THBS2 and CA19-9 | Plasma | LC-MS/MS | ELISA | The combined plasma expression of THBS2 and CA19-9 accurately diagnoses PDAC with an area AUC of 0.952. This biomarker combination demonstrates comparable diagnostic efficacy for both early-stage (AUC = 0.971) and advanced-stage disease (AUC = 0.911). | [^67^](#_ENREF_67) |
| AGP1 and CA19-9 | Tissues and serum | NanoLC-MS/MS | PRM and IHC | AGP1 demonstrates upregulated expression in pancreatic cancer tissues. The combination of AGP1 and CA 19-9 significantly enhances diagnostic efficacy for pancreatic cancer detection, achieving an area AUC of 0.963. | [^30^](#_ENREF_30) |
| CA19-9 and MUC5AC | Serum | ELISA | None | MUC5AC effectively distinguishes early-stage PC from healthy controls with 83% sensitivity and 80% specificity. The combined use of MUC5AC and CA19-9 significantly improves diagnostic accuracy in differentiating resectable cases from controls (p < 0.001). | [^68^](#_ENREF_68) |
| CA19-9, TFPI, and TNC-FNIII-C | Serum | ELISA | None | The biomarker panel comprising TFPI, TNC-FN III-C, and CA19-9 consistently enhances CA19-9's diagnostic performance across all early-stage PDAC cohorts. | [^69^](#_ENREF_69) |
| CA19-9, PROZ, and TNFRSF6B | Serum | LC-MS/MS | ELISA | The AUCs range from 0.816 to 0.971 for PROZ, TNFRSF6B, and CA 19-9, either individually or in combination, in PC versus HC+BC, and from 0.711 to 0.932 in PC Stage I versus HC+BC. | [^70^](#_ENREF_70) |
| AACT, THBS1, HPT, and CA 19−9 | Serum | TMT LC-MS/MS | ELISA | The biomarker panel comprising AACT, THBS1, HPT, and CA19-9 demonstrates strong diagnostic potential for distinguishing PC from normal controls, with an area AUC of 0.99. | [^71^](#_ENREF_71) |
| AGR2, OLFM4, PIGR, COL6A1, SYCN, and CA19–9 | Pancreatic juice, plasma, cell lines | 2D LC-MS/MS | ELISA | The biomarkers AGR2, OLFM4, SYCN, COL6A1, and PIGR are significantly elevated in pancreatic cancer patients. The combination of AGR2, OLFM4, PIGR, SYCN, and COL6A1 with CA19-9 achieves an AUC of 0.98 (95% CI: 0.94–1.00), markedly outperforming CA19-9 alone. | [^72^](#_ENREF_72) |
| CA 19-9, IL.17E, B7.1 and DR6 | Serum | Glycosylation antibody array | None | The panel including CA 19-9, IL.17E, B7.1 and DR6 gave an AUC of 0.988 at 100% sensitivity at 90% specificity for the discrimination of stage 1 PC and healthy controls. | [^73^](#_ENREF_73) |
| CA19-9, TFF1, TFF2, and TFF3 | Serum | ELISA | IHC | Elevated TFF1, TFF2 and TFF3 in PanINs/PC tissues, with TFFs+CA19-9 effectively distinguishing early PC from benign controls (AUC=0.93) and chronic pancreatitis (AUC=0.93). | [^74^](#_ENREF_74) |
| **Abbreviations:** LFQ, label-free quantitative proteomics; ELISA, enzyme linked immunosorbent assay; PDAC, pancreatic ductal adenocarcinomas; AUC, area under curve; RPPA, reverse phase protein array; LC-MS/MS, liquid chromatography-tandem mass spectrometry; IP, immunoprecipitation; qRT-PCR, quantitative reverse transcription polymerase chain reaction; iTRAQ; isobaric tags for relative and absolute quantification; SID-MRM-MS, stable isotope dilution coupled with multiple reactions monitoring mass spectrometry; IHC, immunohistochemistry; MRM-MS, multiple reaction monitoring mass spectrometry; NanoLC-MS/MS, Nanoscale liquid chromatography coupled to tandem mass spectrometry; PRM, parallel reaction monitoring; CTCs, circulating tumor cells; HPLC-TOF MS, high performance liquid chromatography-time of flight mass spectrometry; TMT LC-MS/MS; tandem mass tag liquid chromatography-tandem mass spectrometry; PC, pancreatic cancer; HC, healthy controls; BC, pancreatic benign controls; 2D LC-MS/MS, two-dimensional liquid chromatography-tandem mass spectrometry. | | | | | |

| **Supplementary Table 5.** Some prognosis protein biomarkers in pancreatic cancer identified with proteomics approaches. | | | | | |
| --- | --- | --- | --- | --- | --- |
| **Biomarkers** | **Sample types** | **Proteomic quantification methods** | **Validation techniques** | **Significance** | **Ref.** |
| Calreticulin (CRT) | Cell lines | 2-D electrophoresis and MS/MS | Flow cytometry, IHC and IF | CRT overexpression is associated with poor survival of patients with pancreatic cancer. | [^75^](#_ENREF_75) |
| Cytokeratin-19 | Serum | ELISA | None | CYFRA 21-1 levels were also significantly higher in progressive patients than in patients with disease control (*p* = 0.01). | [^76^](#_ENREF_76) |
| RARRES1 | Blood and cell lines | SILAC-LC-MS/MS | IHC and WB | RARRES1-positive patients with high CTCs counts after curative operation during follow-up had a worse prognosis (*p* = 0.001). | [^77^](#_ENREF_77) |
| FLT3 and PCBP3 | Tissue | LC-MS | None | In PDAC patients, the expression of FLT3 is associated with better survival rates, while the expression of PCBP3 is related to tumor staging and lymph node infiltration. | [^78^](#_ENREF_78) |
| S100A4 | Mouse organoid | HPLC-TOF MS | None | Prognosis (predicting EMT state, disease progression, and survival) | [^79^](#_ENREF_79) |
| PLG, COPS5, FYN, IRF3, ITGB3 and SPTA1 | Tissues | LC-MS/MS | None | Patients with higher COPS5 expression exhibited shorter overall survival (OS) and recurrence-free survival. Additionally, patients with higher expression of PLG, ITGB3, and SPTA1, as well as lower expression of FYN and IRF3, also showed shorter OS. | [^80^](#_ENREF_80) |
| ITGA2 | Tissues and cell lines | NanoLC-MS/MS | IHC and RT-qPCR | High level of ITGA2 expression correlated with shorter progression-free and overall survival of PDAC. | [^81^](#_ENREF_81) |
| 25 proteins | FFPE | NanoLC-MS/MS | PRM-MS | Seven and eighteen proteins were upregulated in patients with "short" and "long" survival, respectively. | [^82^](#_ENREF_82) |
| CLCA1 | Tissue | TMA-IHC | TMA-IHC | Low CLCA1 expression was significantly associated with shorter disease-free survival (11.9 vs. 17.5 months, *p* = 0.042). | [^83^](#_ENREF_83) |
| Yes-associated protein 1 (YAP1) | Tissues | LC–MS/MS (LFQ) | IHC/IF | Compared with the normal control group, YAP1 was significantly upregulated in pancreatic cancer patients, and it was significantly correlated with the decrease in overall survival rate *(p* = 0.001). | [^84^](#_ENREF_84) |
| cofilin-1 | Tissues and serum | 2-D electrophoresis | TMA-IHC | The level of cofilin-1 is elevated in the tissues and sera of patients with PDAC, and it gradually increases during the progression of PDAC (*p* = 0.0034). Patients with a high level of cofilin-1 have a poorer prognosis after surgery (*p* = 0.039). | [^85^](#_ENREF_85) |
| fuco-SERPINA1 | Plasma | iTRAQ | ELISA | Elevated fuco-SERPINA1 levels were associated with higher TNM stage (*p* = 0.024) and poorer prognosis for overall survival (*p* = 0.0083). | [^86^](#_ENREF_86) |
| PTPRM, PTPRB and PSMD11 | Plasma and plasma derived microparticle | SWATH-MS | None | The levels of PTPRM and PTPRB in the plasma of PDAC patients with poor prognosis were significantly decreased, while the level of PSMD11 in the microparticle proteins isolated from the plasma was significantly increased. | [^87^](#_ENREF_87) |
| BASP1 | Pancreas tissues | LC-MS/MS and PRM-MS | TMA-IHC | BASP1 is an independent predictor of prolonged survival in PDAC patients ([HR] 0.468, 95% CI 0.257–0.852, *p* = 0.013) and predicts a favorable response to adjuvant chemotherapy. | [^88^](#_ENREF_88) |
| Fibrinogen | Serum | MALDI-TOF MS | ELISA | The average serum fibrinogen level in PDAC patients was significantly higher than in the control group (*p* < 0.001). Furthermore, PDAC patients with distant metastasis exhibited significantly higher serum fibrinogen levels compared to those without metastasis (*p* = 0.002). Importantly, the median overall survival of patients with low fibrinogen levels (< 1000 ng/mL) was significantly longer than that of patients with high levels (≥ 1000 ng/mL) (*p* < 0.001). | [^89^](#_ENREF_89) |
| H1.3 | Tissues | HPLC-MS | IHC | The histone variant H1.3 was differentially expressed in PDAC and normal pancreatic tissues (*p* = 0.005), and its expression in tumors was associated with lower survival rates. | [^90^](#_ENREF_90) |
| PNMAL1 | Tissues | LC-MS/MS (iTRAQ) | IHC | PNMAL1 is overexpressed in PDAC and is only expressed in trace amounts in adjacent noncancerous tissues. Its positive expression is significantly correlated with well-differentiated tumors and a better outcome for patients. | [^91^](#_ENREF_91) |
| Survivin | Tissues | TMA-IHC | None | The expression of Survivin in PDAC tumors is much higher than in non-tumorous tissues, and it is significantly correlated with poor disease-specific survival rate (*p* = 0.0328). | [^92^](#_ENREF_92) |
| PRELP | Tissues | LC-MS/MS (LFQ) | SRM-MS, IHC and WB | High level of PRELP was associated with shorter postoperative survival in PDAC patients. | [^93^](#_ENREF_93) |
| A panel of 24 proteins | Tissues | SIS-MRM-MS | MRM-MS | Based on the identified 24 key protein biomarkers, patients can be classified into four prognosis-related risk subgroups characterized by protein signatures: stable, exocrine-like, activated, and ECM remodeling, which provides a molecular classification basis for precise treatments (e.g., targeting metabolic/immune pathways). | [^94^](#_ENREF_94) |
| **Abbreviations:** MS/MS, tandem mass spectrometry; IHC, immunohistochemistry; IF, immunofluorescence; ELISA, enzyme linked immunosorbent assay; SILAC-LC-MS/MS, stable isotope labeling by amino acids in cell culture-liquid chromatography-tandem mass spectrometry; WB, western blot; CTCs, circulating tumor cells; LC/MS, liquid chromatography-Mass spectrometry; PDAC, pancreatic ductal adenocarcinomas**;** LC-MS/MS, liquid chromatography-tandem mass spectrometry; HPLC-TOF MS, high performance liquid chromatography-time of flight mass spectrometry; RT-qPCR, quantitative reverse transcription polymerase chain reaction; NanoLC-MS/MS, Nanoscale liquid chromatography coupled to tandem mass spectrometry; FFPE, Formalin-fixed paraffin-embedded tissues; PRM-MS, parallel reaction monitoring mass spectrometry; TMA-IHC, tissue microarrays immunohistochemistry; LFQ, label-free quantitative proteomics; iTRAQ; isobaric tags for relative and absolute quantification; SWATH-MS, sequential window acquisition of all theoretical spectra; MALDI-TOF MS, matrix-assisted laserdesorption/ ionization time of flight mass spectrometry; HPLC-MS, high-throughput high-performance liquid chromatography/mass spectrometry; SRM-MS, selected reaction monitoring mass spectrometry; MRM-MS, multiple reaction monitoring mass spectrometry. | | | | | |

**REFERENCES**

1. Conroy T, Desseigne F, Ychou M, et al. FOLFIRINOX versus gemcitabine for metastatic pancreatic cancer. *N Engl J Med*. May 12 2011;364(19):1817-25. doi:10.1056/NEJMoa1011923

2. Von Hoff DD, Ervin T, Arena FP, et al. Increased survival in pancreatic cancer with nab-paclitaxel plus gemcitabine. *N Engl J Med*. Oct 31 2013;369(18):1691-703. doi:10.1056/NEJMoa1304369

3. Wainberg ZA, Melisi D, Macarulla T, et al. NALIRIFOX versus nab-paclitaxel and gemcitabine in treatment-naive patients with metastatic pancreatic ductal adenocarcinoma (NAPOLI 3): a randomised, open-label, phase 3 trial. *Lancet*. Oct 7 2023;402(10409):1272-1281. doi:10.1016/s0140-6736(23)01366-1

4. Mahalingam D, Shroff RT, Carneiro BA, et al. Preliminary results from the randomized phase 2 study (1801 part 3B) of elraglusib in combination with gemcitabine/nab-paclitaxel (GnP) versus GnP alone in patients (pts) with previously untreated metastatic pancreatic ductal adenocarcinoma (mPDAC). *J Clin Oncol*. 2025;43(16_suppl):4006-4006. doi:10.1200/JCO.2025.43.16_suppl.4006

5. Cui J, Qin S, Zhou Y, et al. Irinotecan hydrochloride liposome HR070803 in combination with 5-fluorouracil and leucovorin in locally advanced or metastatic pancreatic ductal adenocarcinoma following prior gemcitabine-based therapy (PAN-HEROIC-1): a phase 3 trial. *Signal Transduct Target Ther*. Sep 19 2024;9(1):248. doi:10.1038/s41392-024-01948-4

6. De La Fouchardière C, Malka D, Cropet C, et al. Gemcitabine and Paclitaxel Versus Gemcitabine Alone After 5-Fluorouracil, Oxaliplatin, and Irinotecan in Metastatic Pancreatic Adenocarcinoma: A Randomized Phase III PRODIGE 65-UCGI 36-GEMPAX UNICANCER Study. *J Clin Oncol*. Mar 20 2024;42(9):1055-1066. doi:10.1200/jco.23.00795

7. Conroy T, Castan F, Lopez A, et al. Five-Year Outcomes of FOLFIRINOX vs Gemcitabine as Adjuvant Therapy for Pancreatic Cancer: A Randomized Clinical Trial. *JAMA Oncol*. Nov 1 2022;8(11):1571-1578. doi:10.1001/jamaoncol.2022.3829

8. Tempero M, Oh DY, Tabernero J, et al. Ibrutinib in combination with nab-paclitaxel and gemcitabine for first-line treatment of patients with metastatic pancreatic adenocarcinoma: phase III RESOLVE study. *Ann Oncol*. May 2021;32(5):600-608. doi:10.1016/j.annonc.2021.01.070

9. Van Cutsem E, Tempero MA, Sigal D, et al. Randomized Phase III Trial of Pegvorhyaluronidase Alfa With Nab-Paclitaxel Plus Gemcitabine for Patients With Hyaluronan-High Metastatic Pancreatic Adenocarcinoma. *J Clin Oncol*. Sep 20 2020;38(27):3185-3194. doi:10.1200/jco.20.00590

10. Golan T, Hammel P, Reni M, et al. Maintenance Olaparib for Germline BRCA-Mutated Metastatic Pancreatic Cancer. *N Engl J Med*. Jul 25 2019;381(4):317-327. doi:10.1056/NEJMoa1903387

11. Strickler JH, Satake H, George TJ, et al. Sotorasib in KRAS p.G12C-Mutated Advanced Pancreatic Cancer. *N Engl J Med*. Jan 5 2023;388(1):33-43. doi:10.1056/NEJMoa2208470

12. Sha H, Tong F, Ni J, et al. First-line penpulimab (an anti-PD1 antibody) and anlotinib (an angiogenesis inhibitor) with nab-paclitaxel/gemcitabine (PAAG) in metastatic pancreatic cancer: a prospective, multicentre, biomolecular exploratory, phase II trial. *Signal Transduct Target Ther*. Jun 7 2024;9(1):143. doi:10.1038/s41392-024-01857-6

13. Padrón LJ, Maurer DM, O'Hara MH, et al. Sotigalimab and/or nivolumab with chemotherapy in first-line metastatic pancreatic cancer: clinical and immunologic analyses from the randomized phase 2 PRINCE trial. *Nat Med*. Jun 2022;28(6):1167-1177. doi:10.1038/s41591-022-01829-9

14. Philip PA, Sahai V, Bahary N, et al. Devimistat (CPI-613) With Modified Fluorouarcil, Oxaliplatin, Irinotecan, and Leucovorin (FFX) Versus FFX for Patients With Metastatic Adenocarcinoma of the Pancreas: The Phase III AVENGER 500 Study. *J Clin Oncol*. Nov 2024;42(31):3692-3701. doi:10.1200/jco.23.02659

15. Hewitt DB, Nissen N, Hatoum H, et al. A Phase 3 Randomized Clinical Trial of Chemotherapy With or Without Algenpantucel-L (HyperAcute-Pancreas) Immunotherapy in Subjects With Borderline Resectable or Locally Advanced Unresectable Pancreatic Cancer. *Ann Surg*. Jan 1 2022;275(1):45-53. doi:10.1097/sla.0000000000004669

16. Qi C, Gong J, Li J, et al. Claudin18.2-specific CAR T cells in gastrointestinal cancers: phase 1 trial interim results. *Nat Med*. Jun 2022;28(6):1189-1198. doi:10.1038/s41591-022-01800-8

17. Sogawa K, Takano S, Iida F, et al. Identification of a novel serum biomarker for pancreatic cancer, C4b-binding protein α-chain (C4BPA) by quantitative proteomic analysis using tandem mass tags. *Br J Cancer*. Oct 11 2016;115(8):949-956. doi:10.1038/bjc.2016.295

18. Hogendorf P, Durczyński A, Skulimowski A, Kumor A, Poznańska G, Strzelczyk J. Neutrophil Gelatinase-Associated Lipocalin (NGAL) concentration in urine is superior to CA19-9 and Ca 125 in differentiation of pancreatic mass: Preliminary report. *Cancer Biomark*. Mar 11 2016;16(4):537-43. doi:10.3233/cbm-160595

19. Padden J, Ahrens M, Kälsch J, et al. Immunohistochemical Markers Distinguishing Cholangiocellular Carcinoma (CCC) from Pancreatic Ductal Adenocarcinoma (PDAC) Discovered by Proteomic Analysis of Microdissected Cells. *Mol Cell Proteomics*. Mar 2016;15(3):1072-82. doi:10.1074/mcp.M115.054585

20. Melo SA, Luecke LB, Kahlert C, et al. Glypican-1 identifies cancer exosomes and detects early pancreatic cancer. *Nature*. Jul 9 2015;523(7559):177-82. doi:10.1038/nature14581

21. Lewis JM, Vyas AD, Qiu Y, Messer KS, White R, Heller MJ. Integrated Analysis of Exosomal Protein Biomarkers on Alternating Current Electrokinetic Chips Enables Rapid Detection of Pancreatic Cancer in Patient Blood. *ACS Nano*. Apr 24 2018;12(4):3311-3320. doi:10.1021/acsnano.7b08199

22. Goonetilleke KS, Mason JM, Siriwardana P, King NK, France MW, Siriwardena AK. Diagnostic and prognostic value of plasma tumor M2 pyruvate kinase in periampullary cancer: evidence for a novel biological marker of adverse prognosis. *Pancreas*. Apr 2007;34(3):318-24. doi:10.1097/MPA.0b013e31802ee9c7

23. Joergensen MT, Heegaard NH, Schaffalitzky de Muckadell OB. Comparison of plasma Tu-M2-PK and CA19-9 in pancreatic cancer. *Pancreas*. Mar 2010;39(2):243-7. doi:10.1097/MPA.0b013e3181bae8ab

24. Simeone DM, Ji B, Banerjee M, et al. CEACAM1, a novel serum biomarker for pancreatic cancer. *Pancreas*. May 2007;34(4):436-43. doi:10.1097/MPA.0b013e3180333ae3

25. Yoneyama T, Ohtsuki S, Honda K, et al. Identification of IGFBP2 and IGFBP3 As Compensatory Biomarkers for CA19-9 in Early-Stage Pancreatic Cancer Using a Combination of Antibody-Based and LC-MS/MS-Based Proteomics. *PLoS One*. 2016;11(8):e0161009. doi:10.1371/journal.pone.0161009

26. Kendrick ZW, Firpo MA, Repko RC, et al. Serum IGFBP2 and MSLN as diagnostic and prognostic biomarkers for pancreatic cancer. *HPB (Oxford)*. Jul 2014;16(7):670-6. doi:10.1111/hpb.12199

27. Koopmann J, Fedarko NS, Jain A, et al. Evaluation of osteopontin as biomarker for pancreatic adenocarcinoma. *Cancer Epidemiol Biomarkers Prev*. Mar 2004;13(3):487-91.

28. Yang Y, Yan S, Tian H, Bao Y. Macrophage inhibitory cytokine-1 versus carbohydrate antigen 19-9 as a biomarker for diagnosis of pancreatic cancer: A PRISMA-compliant meta-analysis of diagnostic accuracy studies. *Medicine (Baltimore)*. Mar 2018;97(9):e9994. doi:10.1097/md.0000000000009994

29. Guo X, Lv X, Fang C, et al. Dysbindin as a novel biomarker for pancreatic ductal adenocarcinoma identified by proteomic profiling. *Int J Cancer*. Oct 15 2016;139(8):1821-9. doi:10.1002/ijc.30227

30. Zhou Q, Andersson R, Hu D, et al. Alpha-1-acid glycoprotein 1 is upregulated in pancreatic ductal adenocarcinoma and confers a poor prognosis. *Transl Res*. Oct 2019;212:67-79. doi:10.1016/j.trsl.2019.06.003

31. Lee MJ, Na K, Jeong SK, et al. Identification of human complement factor B as a novel biomarker candidate for pancreatic ductal adenocarcinoma. *J Proteome Res*. Nov 7 2014;13(11):4878-88. doi:10.1021/pr5002719

32. Shi Y, Gao W, Lytle NK, et al. Targeting LIF-mediated paracrine interaction for pancreatic cancer therapy and monitoring. *Nature*. May 2019;569(7754):131-135. doi:10.1038/s41586-019-1130-6

33. Ren C, Chen Y, Han C, Fu D, Chen H. Plasma interleukin-11 (IL-11) levels have diagnostic and prognostic roles in patients with pancreatic cancer. *Tumour Biol*. Nov 2014;35(11):11467-72. doi:10.1007/s13277-014-2459-y

34. Wang X, Li Y, Tian H, et al. Macrophage inhibitory cytokine 1 (MIC-1/GDF15) as a novel diagnostic serum biomarker in pancreatic ductal adenocarcinoma. *BMC Cancer*. Aug 8 2014;14:578. doi:10.1186/1471-2407-14-578

35. Chen R, Pan S, Duan X, et al. Elevated level of anterior gradient-2 in pancreatic juice from patients with pre-malignant pancreatic neoplasia. *Mol Cancer*. Jun 15 2010;9:149. doi:10.1186/1476-4598-9-149

36. Terai K, Jiang M, Tokuyama W, et al. Levels of soluble LR11/SorLA are highly increased in the bile of patients with biliary tract and pancreatic cancers. *Clin Chim Acta*. Jun 1 2016;457:130-6. doi:10.1016/j.cca.2016.04.010

37. Zhao J, Guo M, Song Y, et al. Serum exosomal and serum glypican-1 are associated with early recurrence of pancreatic ductal adenocarcinoma. *Front Oncol*. 2022;12:992929. doi:10.3389/fonc.2022.992929

38. Yang KS, Im H, Hong S, et al. Multiparametric plasma EV profiling facilitates diagnosis of pancreatic malignancy. *Sci Transl Med*. May 24 2017;9(391)doi:10.1126/scitranslmed.aal3226

39. Zheng J, Hernandez JM, Doussot A, et al. Extracellular matrix proteins and carcinoembryonic antigen-related cell adhesion molecules characterize pancreatic duct fluid exosomes in patients with pancreatic cancer. *HPB (Oxford)*. Jul 2018;20(7):597-604. doi:10.1016/j.hpb.2017.12.010

40. Yoshioka Y, Shimomura M, Saito K, et al. Circulating cancer-associated extracellular vesicles as early detection and recurrence biomarkers for pancreatic cancer. *Cancer Sci*. Oct 2022;113(10):3498-3509. doi:10.1111/cas.15500

41. Charles Jacob HK, Signorelli R, Charles Richard JL, et al. Identification of novel early pancreatic cancer biomarkers KIF5B and SFRP2 from "first contact" interactions in the tumor microenvironment. *J Exp Clin Cancer Res*. Aug 24 2022;41(1):258. doi:10.1186/s13046-022-02425-y

42. Jin H, Liu P, Wu Y, et al. Exosomal zinc transporter ZIP4 promotes cancer growth and is a novel diagnostic biomarker for pancreatic cancer. *Cancer Sci*. Sep 2018;109(9):2946-2956. doi:10.1111/cas.13737

43. Costa-Silva B, Aiello NM, Ocean AJ, et al. Pancreatic cancer exosomes initiate pre-metastatic niche formation in the liver. *Nat Cell Biol*. Jun 2015;17(6):816-26. doi:10.1038/ncb3169

44. Liang K, Liu F, Fan J, et al. Nanoplasmonic Quantification of Tumor-derived Extracellular Vesicles in Plasma Microsamples for Diagnosis and Treatment Monitoring. *Nat Biomed Eng*. 2017;1doi:10.1038/s41551-016-0021

45. Giampieri R, Piva F, Occhipinti G, et al. Clinical impact of different exosomes' protein expression in pancreatic ductal carcinoma patients treated with standard first line palliative chemotherapy. *PLoS One*. 2019;14(5):e0215990. doi:10.1371/journal.pone.0215990

46. Wei Q, Zhang J, Li Z, Wei L, Ren L. Serum Exo-EphA2 as a Potential Diagnostic Biomarker for Pancreatic Cancer. *Pancreas*. Oct 2020;49(9):1213-1219. doi:10.1097/mpa.0000000000001660

47. Zhang W, Wang L, Li D, et al. Phenotypic profiling of pancreatic ductal adenocarcinoma plasma-derived small extracellular vesicles for cancer diagnosis and cancer stage prediction: a proof-of-concept study. *Anal Methods*. Jun 16 2022;14(23):2255-2265. doi:10.1039/d2ay00536k

48. Lux A, Kahlert C, Grützmann R, Pilarsky C. c-Met and PD-L1 on Circulating Exosomes as Diagnostic and Prognostic Markers for Pancreatic Cancer. *Int J Mol Sci*. Jul 5 2019;20(13)doi:10.3390/ijms20133305

49. Kimura H, Yamamoto H, Harada T, et al. CKAP4, a DKK1 Receptor, Is a Biomarker in Exosomes Derived from Pancreatic Cancer and a Molecular Target for Therapy. *Clin Cancer Res*. Mar 15 2019;25(6):1936-1947. doi:10.1158/1078-0432.Ccr-18-2124

50. Leca J, Martinez S, Lac S, et al. Cancer-associated fibroblast-derived annexin A6+ extracellular vesicles support pancreatic cancer aggressiveness. *J Clin Invest*. Nov 1 2016;126(11):4140-4156. doi:10.1172/jci87734

51. Verel-Yilmaz Y, Fernández JP, Schäfer A, et al. Extracellular Vesicle-Based Detection of Pancreatic Cancer. *Front Cell Dev Biol*. 2021;9:697939. doi:10.3389/fcell.2021.697939

52. Odaka H, Hiemori K, Shimoda A, Akiyoshi K, Tateno H. CD63-positive extracellular vesicles are potential diagnostic biomarkers of pancreatic ductal adenocarcinoma. *BMC Gastroenterol*. Mar 28 2022;22(1):153. doi:10.1186/s12876-022-02228-7

53. Xie Z, Gao Y, Ho C, et al. Exosome-delivered CD44v6/C1QBP complex drives pancreatic cancer liver metastasis by promoting fibrotic liver microenvironment. *Gut*. Mar 2022;71(3):568-579. doi:10.1136/gutjnl-2020-323014

54. Li J, Li Y, Chen S, et al. Highly Sensitive Exosome Detection for Early Diagnosis of Pancreatic Cancer Using Immunoassay Based on Hierarchical Surface-Enhanced Raman Scattering Substrate. *Small Methods*. Jun 2022;6(6):e2200154. doi:10.1002/smtd.202200154

55. Asada T, Nakahata S, Fauzi YR, et al. Integrin α6A (ITGA6A)-type Splice Variant in Extracellular Vesicles Has a Potential as a Novel Marker of the Early Recurrence of Pancreatic Cancer. *Anticancer Res*. Apr 2022;42(4):1763-1775. doi:10.21873/anticanres.15653

56. Marin AM, Batista M, Korte de Azevedo AL, et al. Screening of Exosome-Derived Proteins and Their Potential as Biomarkers in Diagnostic and Prognostic for Pancreatic Cancer. *Int J Mol Sci*. Aug 9 2023;24(16)doi:10.3390/ijms241612604

57. Osteikoetxea X, Benke M, Rodriguez M, et al. Detection and proteomic characterization of extracellular vesicles in human pancreatic juice. *Biochem Biophys Res Commun*. Apr 30 2018;499(1):37-43. doi:10.1016/j.bbrc.2018.03.107

58. Capello M, Bantis LE, Scelo G, et al. Sequential Validation of Blood-Based Protein Biomarker Candidates for Early-Stage Pancreatic Cancer. *J Natl Cancer Inst*. Apr 1 2017;109(4)doi:10.1093/jnci/djw266

59. Liu X, Zheng W, Wang W, et al. A new panel of pancreatic cancer biomarkers discovered using a mass spectrometry-based pipeline. *Br J Cancer*. Mar 20 2018;118(6):e15. doi:10.1038/bjc.2018.5

60. Park J, Lee E, Park KJ, et al. Large-scale clinical validation of biomarkers for pancreatic cancer using a mass spectrometry-based proteomics approach. *Oncotarget*. Jun 27 2017;8(26):42761-42771. doi:10.18632/oncotarget.17463

61. Park J, Choi Y, Namkung J, et al. Diagnostic performance enhancement of pancreatic cancer using proteomic multimarker panel. *Oncotarget*. Nov 3 2017;8(54):93117-93130. doi:10.18632/oncotarget.21861

62. Choi YJ, Yoon W, Lee A, et al. Diagnostic model for pancreatic cancer using a multi-biomarker panel. *Ann Surg Treat Res*. Mar 2021;100(3):144-153. doi:10.4174/astr.2021.100.3.144

63. Lee DH, Yoon W, Lee A, et al. Multi-biomarker panel prediction model for diagnosis of pancreatic cancer. *J Hepatobiliary Pancreat Sci*. Jan 2023;30(1):122-132. doi:10.1002/jhbp.986

64. Ben-Ami R, Wang QL, Zhang J, et al. Protein biomarkers and alternatively methylated cell-free DNA detect early stage pancreatic cancer. *Gut*. Mar 7 2024;73(4):639-648. doi:10.1136/gutjnl-2023-331074

65. Kim J, Bamlet WR, Oberg AL, et al. Detection of early pancreatic ductal adenocarcinoma with thrombospondin-2 and CA19-9 blood markers. *Sci Transl Med*. Jul 12 2017;9(398)doi:10.1126/scitranslmed.aah5583

66. Jenkinson C, Elliott VL, Evans A, et al. Decreased Serum Thrombospondin-1 Levels in Pancreatic Cancer Patients Up to 24 Months Prior to Clinical Diagnosis: Association with Diabetes Mellitus. *Clin Cancer Res*. Apr 1 2016;22(7):1734-1743. doi:10.1158/1078-0432.Ccr-15-0879

67. Le Large TYS, Meijer LL, Paleckyte R, et al. Combined Expression of Plasma Thrombospondin-2 and CA19-9 for Diagnosis of Pancreatic Cancer and Distal Cholangiocarcinoma: A Proteome Approach. *Oncologist*. Apr 2020;25(4):e634-e643. doi:10.1634/theoncologist.2019-0680

68. Kaur S, Smith LM, Patel A, et al. A Combination of MUC5AC and CA19-9 Improves the Diagnosis of Pancreatic Cancer: A Multicenter Study. *Am J Gastroenterol*. Jan 2017;112(1):172-183. doi:10.1038/ajg.2016.482

69. Balasenthil S, Huang Y, Liu S, et al. A Plasma Biomarker Panel to Identify Surgically Resectable Early-Stage Pancreatic Cancer. *J Natl Cancer Inst*. Aug 1 2017;109(8)doi:10.1093/jnci/djw341

70. Wu X, Zhang ZX, Chen XY, et al. A Panel of Three Biomarkers Identified by iTRAQ for the Early Diagnosis of Pancreatic Cancer. *Proteomics Clin Appl*. Sep 2019;13(5):e1800195. doi:10.1002/prca.201800195

71. Nie S, Lo A, Wu J, et al. Glycoprotein biomarker panel for pancreatic cancer discovered by quantitative proteomics analysis. *J Proteome Res*. Apr 4 2014;13(4):1873-84. doi:10.1021/pr400967x

72. Makawita S, Smith C, Batruch I, et al. Integrated proteomic profiling of cell line conditioned media and pancreatic juice for the identification of pancreatic cancer biomarkers. *Mol Cell Proteomics*. Oct 2011;10(10):M111.008599. doi:10.1074/mcp.M111.008599

73. Aronsson L, Andersson R, Bauden M, Andersson B, Bygott T, Ansari D. High-density and targeted glycoproteomic profiling of serum proteins in pancreatic cancer and intraductal papillary mucinous neoplasm. *Scand J Gastroenterol*. Dec 2018;53(12):1597-1603. doi:10.1080/00365521.2018.1532020

74. Jahan R, Ganguly K, Smith LM, et al. Trefoil factor(s) and CA19.9: A promising panel for early detection of pancreatic cancer. *EBioMedicine*. Apr 2019;42:375-385. doi:10.1016/j.ebiom.2019.03.056

75. Matsukuma S, Yoshimura K, Ueno T, et al. Calreticulin is highly expressed in pancreatic cancer stem-like cells. *Cancer Sci*. Nov 2016;107(11):1599-1609. doi:10.1111/cas.13061

76. Boeck S, Wittwer C, Heinemann V, et al. Cytokeratin 19-fragments (CYFRA 21-1) as a novel serum biomarker for response and survival in patients with advanced pancreatic cancer. *Br J Cancer*. Apr 30 2013;108(8):1684-94. doi:10.1038/bjc.2013.158

77. Nitschke C, Markmann B, Tölle M, et al. Characterization of RARRES1 Expression on Circulating Tumor Cells as Unfavorable Prognostic Marker in Resected Pancreatic Ductal Adenocarcinoma Patients. *Cancers (Basel)*. Sep 10 2022;14(18)doi:10.3390/cancers14184405

78. Ger M, Kaupinis A, Petrulionis M, et al. Proteomic Identification of FLT3 and PCBP3 as Potential Prognostic Biomarkers for Pancreatic Cancer. *Anticancer Res*. Oct 2018;38(10):5759-5765. doi:10.21873/anticanres.12914

79. Low RRJ, Fung KY, Gao H, et al. S100 family proteins are linked to organoid morphology and EMT in pancreatic cancer. *Cell Death Differ*. May 2023;30(5):1155-1165. doi:10.1038/s41418-023-01126-z

80. Kou YQ, Yang YP, Pan ZJ, et al. Prognostic-Related Biomarkers in Pancreatic Ductal Adenocarcinoma Correlating with Immune Infiltrates Based on Proteomics. *Med Sci Monit*. Mar 11 2023;29:e938785. doi:10.12659/msm.938785

81. Gregori A, Bergonzini C, Capula M, et al. Prognostic Significance of Integrin Subunit Alpha 2 (ITGA2) and Role of Mechanical Cues in Resistance to Gemcitabine in Pancreatic Ductal Adenocarcinoma (PDAC). *Cancers (Basel)*. Jan 19 2023;15(3)doi:10.3390/cancers15030628

82. Hu D, Ansari D, Pawłowski K, et al. Proteomic analyses identify prognostic biomarkers for pancreatic ductal adenocarcinoma. *Oncotarget*. Feb 9 2018;9(11):9789-9807. doi:10.18632/oncotarget.23929

83. Hu D, Ansari D, Zhou Q, et al. Calcium-activated chloride channel regulator 1 as a prognostic biomarker in pancreatic ductal adenocarcinoma. *BMC Cancer*. Nov 12 2018;18(1):1096. doi:10.1186/s12885-018-5013-2

84. Zhou Q, Bauden M, Andersson R, et al. YAP1 is an independent prognostic marker in pancreatic cancer and associated with extracellular matrix remodeling. *J Transl Med*. Feb 13 2020;18(1):77. doi:10.1186/s12967-020-02254-7

85. Satoh M, Takano S, Sogawa K, et al. Immune-complex level of cofilin-1 in sera is associated with cancer progression and poor prognosis in pancreatic cancer. *Cancer Sci*. Apr 2017;108(4):795-803. doi:10.1111/cas.13181

86. Wu CC, Lu YT, Yeh TS, Chan YH, Dash S, Yu JS. Identification of Fucosylated SERPINA1 as a Novel Plasma Marker for Pancreatic Cancer Using Lectin Affinity Capture Coupled with iTRAQ-Based Quantitative Glycoproteomics. *Int J Mol Sci*. Jun 4 2021;22(11)doi:10.3390/ijms22116079

87. Sahni S, Krisp C, Molloy MP, et al. PSMD11, PTPRM and PTPRB as novel biomarkers of pancreatic cancer progression. *Biochim Biophys Acta Gen Subj*. Nov 2020;1864(11):129682. doi:10.1016/j.bbagen.2020.129682

88. Zhou Q, Andersson R, Hu D, et al. Quantitative proteomics identifies brain acid soluble protein 1 (BASP1) as a prognostic biomarker candidate in pancreatic cancer tissue. *EBioMedicine*. May 2019;43:282-294. doi:10.1016/j.ebiom.2019.04.008

89. Chung KH, Lee JC, Lee J, et al. Serum fibrinogen as a diagnostic and prognostic biomarker for pancreatic ductal adenocarcinoma. *Pancreatology*. Oct 2020;20(7):1465-1471. doi:10.1016/j.pan.2020.06.010

90. Bauden M, Kristl T, Sasor A, et al. Histone profiling reveals the H1.3 histone variant as a prognostic biomarker for pancreatic ductal adenocarcinoma. *BMC Cancer*. Dec 2 2017;17(1):810. doi:10.1186/s12885-017-3834-z

91. Kuwae Y, Kakehashi A, Wakasa K, et al. Paraneoplastic Ma Antigen-Like 1 as a Potential Prognostic Biomarker in Human Pancreatic Ductal Adenocarcinoma. *Pancreas*. Jan 2015;44(1):106-15. doi:10.1097/mpa.0000000000000220

92. Zhou L, Lu J, Liang ZY, et al. High nuclear Survivin expression as a poor prognostic marker in pancreatic ductal adenocarcinoma. *J Surg Oncol*. Dec 2018;118(7):1115-1121. doi:10.1002/jso.25253

93. Iuga C, Seicean A, Iancu C, et al. Proteomic identification of potential prognostic biomarkers in resectable pancreatic ductal adenocarcinoma. *Proteomics*. Apr 2014;14(7-8):945-55. doi:10.1002/pmic.201300402

94. Son M, Kim H, Han D, et al. A Clinically Applicable 24-Protein Model for Classifying Risk Subgroups in Pancreatic Ductal Adenocarcinomas using Multiple Reaction Monitoring-Mass Spectrometry. *Clin Cancer Res*. Jun 15 2021;27(12):3370-3382. doi:10.1158/1078-0432.Ccr-20-3513
